# Bioinspired cross-medium wall-climbing robot with high-performance adhesion and contact adaptability

**DOI:** 10.1126/sciadv.aea8014

**Published:** 2026-01-07

**Authors:** Haoran Liu, Hongmiao Tian, Zexi Zheng, Huiming Liu, Hechuan Ma, Zhihao Deng, Duorui Wang, Jinyu Zhang, Xiangming Li, Xiaoliang Chen, Chunhui Wang, Xiaoming Chen, Qiguang He, Jinyou Shao

**Affiliations:** ^1^ Micro- and Nano-Technology Research Center, State Key Laboratory for Manufacturing Systems Engineering, Xi’an Jiaotong University, Xi’an, Shaanxi 710049, China.; ^2^ Department of Mechanical and Automation Engineering, The Chinese University of Hong Kong, Hong Kong 999077, China.; ^3^ Frontier Institute of Science and Technology (FIST), Xi’an Jiaotong University, Xi’an, Shaanxi 710049, China.

## Abstract

Wall-climbing robots (WCRs) using numerous attachment/grasping mechanisms replace humans in executing repetitive or challenging tasks in space-confined, high-risk, or radioactive environments, garnering substantial research interest. Nevertheless, their application remains limited to environment- and surface-specific scenarios. To this end, we present a contact-adaptable, peeling-resistant, and cross-medium WCR integrated with gecko- and octopus-inspired self-adaptive rigid-soft hybrid tracks. The hollow mushroom-shaped adhesive microstructures (HMSAMSs) on the robot tracks simultaneously couple the adhesive structural morphologies of gecko and octopus as well as inherit their functions. These microstructures demonstrate superior normal and tangential adhesion forces and adhesion-to-preload ratios in dry and underwater environments, endowing the WCR with stable cross-medium performance. Furthermore, we construct discrete HMSAMS patches and rigid-soft components in robot track, respectively mimicking biological adhesion’s mechanical decoupling and bone-muscle functions, which effectively prevent interface crack propagation, improve peeling threshold, and enhance contact adaptability. The WCR with aforementioned advantages substantially adapts to diverse material surfaces in complex multimedia environments, accelerating its universal application.

## Teaser

A cross-medium wall-climbing robot expeditiously crawls on uneven vertical and inverted surfaces in multimedia environments.

## INTRODUCTION

**Fig. 1.**
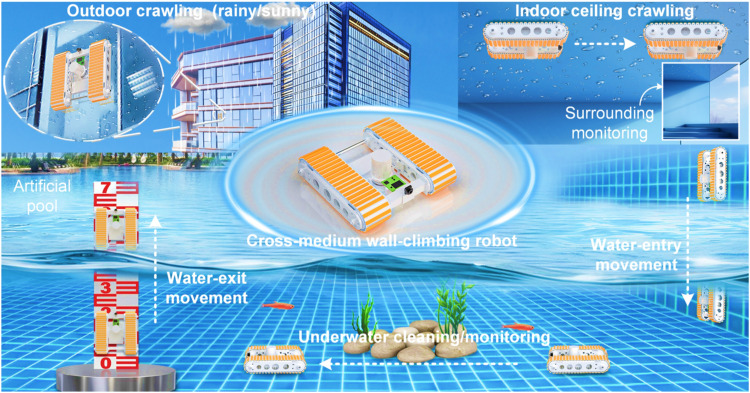
Depiction of the mission profile and macrostructure of cross-medium WCR. The cross-medium WCR assisted by all-in-one rigid-soft hybrid robot tracks exhibits excellent amphibious wall-climbing ability on varying object surfaces (e.g., metal, glass, and ceramic tiles) under various conditions (e.g. dry, moist, or underwater). Specifically, persistent surveillance from building vantage points under all weather conditions, underwater concealment (to avoid detection) with on-demand surfacing, or hazardous environment operations can be readily realized by the cross-medium WCR. Software credit: Cinema 4D.

Wall-climbing robots (WCRs) with high-performance attachment/grasping structures can readily substitute humans to handle various repetitive or challenging tasks, e.g., on-site detection or monitoring, nuclear power pipeline inspection, salvage and disaster relief, in space-confined, high-risk, or harsh surroundings (*1*–*5*). Nevertheless, recently developed WCRs are more inclined to move flexibly in specific scenarios (e.g., climbing on certain surfaces of given materials under dry or wet conditions, not to mention crossing the water-air interface), severely restricting the widespread application of WCRs. Ideally, amphibious WCRs with versatile dry/wet adhesive systems would crawl fluidly among terrestrial, underwater, and humid zones—including air-water transitions—while maintaining high-performance adhesion on diverse material surfaces and adaptive contact with uneven topographies. Such advantageous characteristics would help WCRs acclimatize to complex multimedia environments and expand real-world application scenarios more efficiently (e.g., monitoring surroundings on and under water surface via water-entry and water-exit locomotion in the pond, or long-time surveillance from indoor/outdoor vantage points in Fig. 1). Unfortunately, few existing WCRs have such cross-medium capacity and successfully navigate such hypothetical scenarios (*6*, *7*).

Recently, numerous off-the-shelf WCRs with different types of adhesive/grasping mechanisms, such as the mechanical (hook or claw) (*8*–*11*), magnetic (permanent magnets or electromagnets) (*4*, *5*, *12*), pneumatic (suction cups or propeller) (*13*–*17*), electrostatic (*3*, *18*–*20*), and biomimetic (gecko-, octopus-, or remora-inspired structures) (*21*–*25*), have stably crawled on specific surfaces (e.g., ferromagnetic or extremely coarse surfaces) in single media (i.e., either in the air or underwater). Actually, foremost among the aforementioned mechanisms is their adhesive/grasping performance, which may drop abruptly or vanish entirely due to environmental or surface-related changes, severely restricting WCRs’ movement across multimaterial surfaces in multi- or cross-medium environments. In particular, mechanical and magnetic mechanisms may slip on smooth surfaces with water beads or nonferromagnetic surfaces, whereas pneumatic and electrostatic ones may lose efficacy due to the disappearance of tangential forces on underwater smooth surfaces. Gecko-inspired dry adhesives excel only on dry surfaces, while octopus- and remora-inspired structures mainly exhibit excellent adhesive performance underwater. Meanwhile, due to the complex and dynamic force conditions during water-exit processes, current WCRs are more likely to overturn or slip on uneven or undulating vertical surfaces. Considering that, the lack of high-strength cross-medium adhesion in multimedia environments, the preference for specific crawling surface materials, and unfriendly contact adaptability to different surface morphologies are now the key hurdles severely hindering WCRs’ application in multi- and trans-medium environments.

**Fig. 2.**
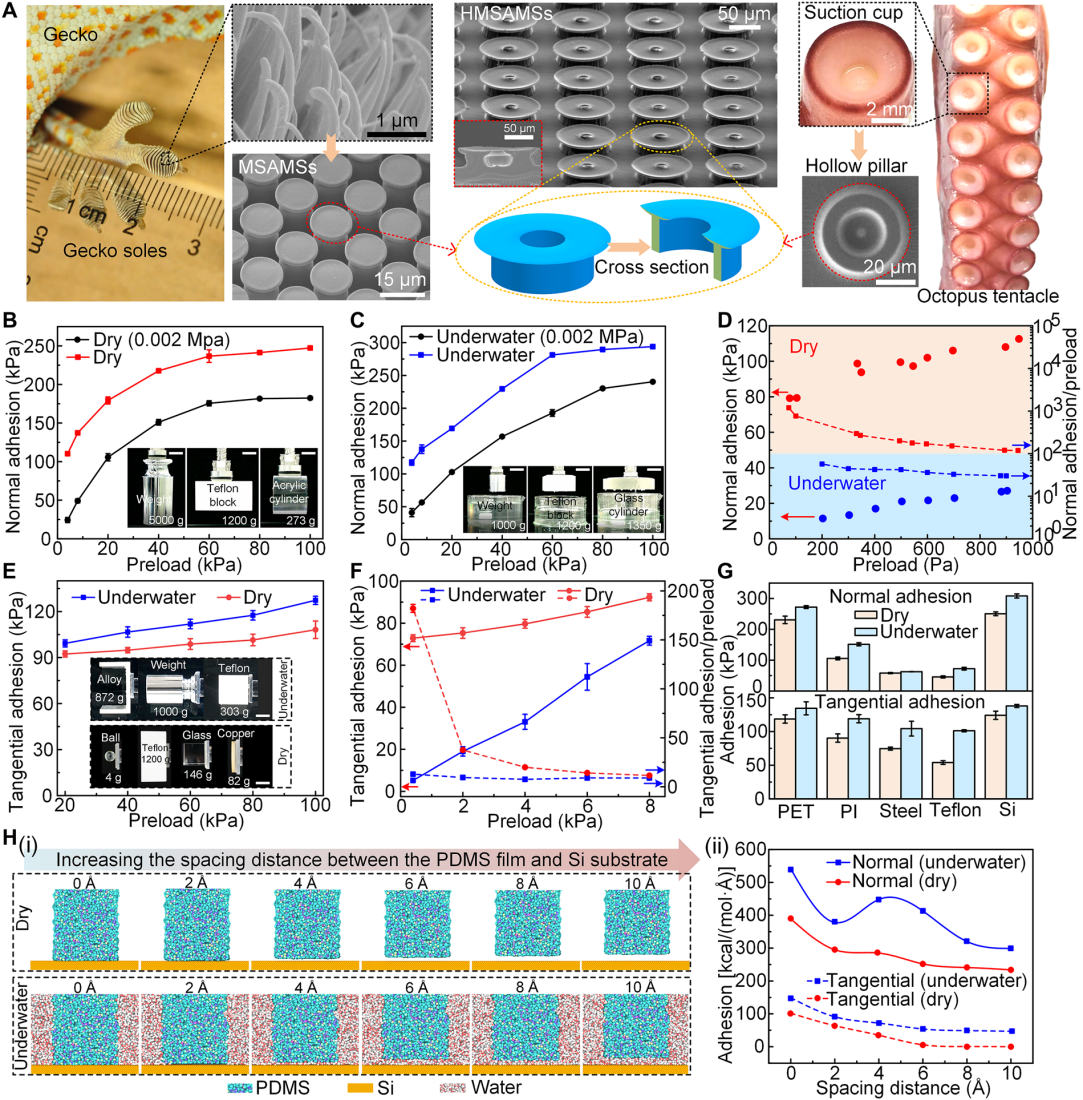
Micromorphological features and adhesion performance of bioinspired HMSAMSs. (**A**) The inspiration source and micromorphology of the proposed HMSAMSs. The gecko-inspired MSAMSs exhibit outstanding adhesion performance under dry condition due to van der Waals forces. The octopus-inspired hollow micropillars, using the suction cup effect, show superior underwater adhesive ability. Hence, HMSAMSs simultaneously having mushroom-like morphology and hollow cavities are proposed. (**B**) Normal adhesion strength of HMSAMS patch under different preloads in dry (standard air pressure, 0.101 MPa) and dry (0.002 MPa) environments. The inset depicts the adhesion performance of the HMSAMS patch on various objects under dry conditions: weight (5000 g), Teflon plate (1200 g), and cylindrical PMMA (273 g). Scale bars, 5 cm. (**C**) Preload-dependent normal adhesion strength of the HMSAMS patch underwater (standard air pressure) and underwater (0.002 MPa). The inset demonstrates the underwater adhesion ability of the HMSAMS patch on different objects: weight (2000 g), Teflon plate (1200 g), and glass cylinder (1350 g). Scale bars, 5 cm. (**D**) Normal adhesion strength and normal adhesion-to-preload ratio of the HMSAMS patch under small preload in dry and underwater environments. (**E**) Tangential adhesion strength of the HMSAMS patch under various preloads (20 to 100 kPa) in dry and underwater environments. The inset shows the tangential adhesion performance under dry and underwater conditions. Scale bars, 5 cm. (**F**) Tangential adhesion strength and tangential adhesion-to-preload ratio of the HMSAMS patch under small preload in dry and underwater environments. (**G**) Maximum adhesion of the HMSAMS patch on surfaces of different materials. Preload, 80 kPa. (**H**) (i) Molecular dynamics (MD) simulation revealing the (ii) effect of spacing distance between a thin PDMS film and an Si substrate on adhesion. Thereinto, the spacing distance of models (i) was set as 0, 2, 4, 6, 8, and 10 Å, respectively.

Fortunately, nature offers researchers multiple combinable and referable inspirations from reptiles to address the aforementioned WCRs’ challenges. For instance, geckos, relying mainly on van der Waals forces (insensible to materials), agilely crawl on vertical walls or ceilings using the multilayered spatula-shaped, nanometer-sized structures on their soles (*26*). Octopuses leverage negative pressure effect (without material preference) of discrete hollow structures on their tentacles to rapidly transition between positive/negative and vertical surfaces underwater (Fig. 2A) (*27*). These discrete adhesive structures on gecko soles and octopus tentacles effectively suppress crack propagation of adhesive structures on different contact surfaces. High-module bones or cartilages in these soles or tentacles optimize stress distribution of adhesive structures, enhancing their attachment strength. Moreover, soft muscles in these soles and tentacles significantly enhance conformal contact stability between adhesive structures and target surfaces. Ideally, by coupling gecko-inspired mushroom-shaped dry adhesive microstructures with octopus-inspired hollow pillars, as well as inheriting their individual functions, a single adhesive structure would achieve high-performance normal and tangential adhesion on surfaces of different materials in both dry and underwater environments. Meanwhile, if the adhesive patches (i.e., soft shell) incorporating aforementioned coupled adhesive microstructures could also embed rigid foreign matter internally (i.e., rigid core) to form core-shell adhesive patches and discretely attach to a well-designed soft substrate (i.e., forming an all-in-one soft-rigid hybrid structure—robot track), then they could vividly imitate: suppression of interface cracks, high-strength adhesion, and conformal contact adaptability. Such a robot track, the core component of WCRs, would enable WCRs to achieve dynamic multi- and trans-medium motion on nonparallel or undulating surfaces regardless of materials. However, few studies hitherto have focused on this approach.

To this end, inspired by the aforementioned concept, this work proposes a cross-medium contact-adaptive and peeling-resistant WCR assisted by all-in-one soft-rigid hybrid tracks. The proposed WCR can dynamically crawl on dry and underwater surfaces of different materials, cross the water-air boundary at different inclination angles, move against torrential currents, and traverse obstacles or gaps to perform detection and surveillance tasks. Thereinto, the developed hollow mushroom-shaped adhesive microstructures (HMSAMSs) on robot tracks combine gecko-inspired mushroom-shaped morphology with octopus-inspired hollow cavities, simultaneously achieving normal and tangential adhesion strength in both dry and underwater conditions through combined intermolecular forces and negative pressure effects. The core-shell HMSAMS patches with embedded rigid cores are discretely distributed on the top layer of the robot tracks, respectively mimicking the discrete behavior of adhesive structures and the function of high-modulus bones or cartilages in gecko soles and octopus tentacles, whose configuration markedly suppresses interface crack propagation, optimizes interface stress distribution, and improves the peeling strength of the robot tracks. Furthermore, discrete pillars in the robot track substrate replicate the low effective elastic modulus of soft muscles in biological soles and tentacles, endowing the proposed robot with superior contact adaptability on uneven surfaces. Meanwhile, the high adhesion-to-preload ratio of HMSAMSs effectively prevents slipping and overturning of the robot during low-preload attachment. Such hierarchical architecture of robot tracks with aforementioned advantages—high-performance multimedia adhesion, enhanced peeling resistance, and conformal contact adaptability—empowers the WCRs with reliable cross-medium locomotion capability on vertical surfaces, substantially expanding WCRs’ application on diverse material surfaces in multimedia environments.

## RESULTS

### Microstructure and adhesion performance of HMSAMS

MSAMSs, inspired by spatula-terminated setae on gecko toes, demonstrate durable, repeatable, and reversible adhesion through van der Waals force in dry environments (*28*). Under wet or underwater conditions, octopus-inspired hollow micropillars using suction cup effect can firmly and repeatably attach to the surfaces of numerous objects without nonchemical contamination (*25*, *29*). By combining the morphology of MSAMSs and hollow micropillars, as well as inheriting their advantageous properties, HMSAMSs were developed (Fig. 2A and fig. S1). These featured cap brims, bidirectionally extending from hollow pillar tops (inset of Fig. 2A), were slightly curled up like the edges of suction cups, which were more inclined to realize intimate contact with object surfaces under low preload. Compared to MSAMSs, hollow micropillars, and micropillars, the HMSAMSs (outer diameter, 50 μm; inner diameter, 30 μm, and center-to-center spacing, 80 μm) exhibited highest normal adhesion in dry and underwater environments, which was roughly 1.57 (dry) and 1.7 (underwater) times the sum of normal adhesion of MSAMSs and the hollow micropillars (fig. S2 and text S1). The enhanced adhesion performance enabled single-environment (dry or wet) applications of HMSAMSs transitioning to multi- or cross-medium ones. The influence of structural parameters on the adhesion performance of HMSAMS patch was quantitatively studied in text S2 and fig. S3.

To systematically characterize the dry and underwater adhesion performance of HMSAMSs, experiments under different conditions were performed by adjusting the surrounding air pressure (Fig. 2, B and C). In particular, adhesion measurements were performed under standard atmospheric condition (0.101 MPa) if no specific instructions were given. The normal adhesion of HMSAMSs respectively increased from 100.2 ± 2.52 to 236.73 ± 8.12 kPa in dry environment and from 24.3 ± 3.27 to 175.53 ± 3.25 kPa in 0.002-MPa dry condition (the vacuum chamber pressure was 0.002 MPa), when the preload was <60 kPa. Meanwhile, when the preload reached 100 kPa, it respectively remained at ~240 kPa in dry condition and ~180 kPa in 0.002-MPa dry condition, vividly revealing the possible application in ambient pressure and vacuum environments. Under identical preload (range, 4 to 100 kPa), the normal adhesion in the 0.101-MPa dry environment was always higher than that in the 0.002-MPa dry environment (Fig. 2B), which was attributed to the change in air pressure. Notably, the excellent normal adhesion at 0.002 MPa was primarily ascribed to the interaction forces between molecules (van der Waals forces). Underwater, the variation trend of preload-dependent normal adhesion was similar to that under dry conditions (Fig. 2C), being stable at ~290 kPa (0.101 MPa) and ~230 kPa (0.002 MPa). The approximate 60-kPa difference in underwater normal adhesion between 0.101- and 0.002-MPa conditions was primarily caused by air pressure effects. At 0.002 MPa, normal adhesion might also have been generated by intermolecular forces (including water molecule interactions) (Fig. 2C). Given the excellent normal adhesion performance of the HMSAMS patch, higher than previous polydimethylsiloxane (PDMS)–based microstructures (*24*, *25*, *29*–*39*) (table S1), the insets in Fig. 2 (B and C) demonstrate its grasping ability on different objects (materials and shapes) in dry [weight (5000 g), Teflon plate (1200 g), and cylindrical polymethyl methacrylate) (PMMA; 273 g)] and underwater [weight (2000 g), Teflon plate (1200 g), and glass cylinder (1350 g)] environments (figs. S4 and S5 and movies S1 and S2). Moreover, in both environments, no attenuation of the normal adhesion of HMSAMS patch under larger preloads (120 to 300 kPa) occurred (fig. S6), illustrating the robust adhesion performance and compression resistance of the proposed microstructures.

In addition to high preload conditions, ultralow preload also triggered high-performance normal adhesion (Fig. 2D). More specifically, in dry environment, normal adhesion reached 79.2 kPa under a preload of 100 Pa, with the corresponding normal adhesion-to-preload ratio increasing to 792. Moreover, the normal adhesion and normal adhesion-to-preload ratio under dry condition increased to 112.6 kPa and decreased to 112.6, respectively, when the preload increased from 100 Pa to 1 kPa. Similarly, the variation trends of normal adhesion and normal adhesion-to-preload ratio underwater, which were both governed by the preload, were similar to those observed in the dry environment. Thereinto, the normal adhesion maintained 11.4 kPa even when the preload was reduced to 200 Pa (normal adhesion-to-preload ratio, 57). Notably, the aforementioned normal adhesion-to-preload ratios of HMSAMS patch in dry and underwater environments were highest compared to previous reported ratios of microstructures (table S2) (*24*, *25*, *29*–*39*). The inward- and outward-extending thin cap brims of HMSAMS could better seal its hollow cavity (i.e., enhancing the negative pressure contribution) and provide an additional contact area compared to the conventional MSAMS with one cap brim during detachment (i.e., increasing the number of interacting molecules), which might jointly contribute to the improvement of the adhesion performance and adhesion-to-preload ratio in both dry and underwater environments.

Normal HMSAMS adhesion exceeds 100 kPa at 0.002-MPa atmospheric pressure in both dry and underwater environments, suggesting that factors beyond suction cup forces, such as intermolecular van der Waals forces and friction, likely contribute significantly to adhesion. In other words, these direction-independent factors may endow HMSAMSs with desirable tangential adhesion capabilities. Therefore, the tangential adhesion of the HMSAMS patch in dry and underwater environments was measured. Tangential adhesion increased steadily to its maximum value (dry, 108.13 ± 5.68 kPa; and underwater, 127.3 ± 2.68 kPa) under high preload (20 to 100 kPa; Fig. 2E) and sharply to peak (dry, 92.3 ± 1.68 kPa; and underwater, 71.65 ± 2.11 kPa) under low preload (0.4 to 8 kPa; Fig. 2F). Under identical high preload, the underwater tangential adhesion was higher than that in dry condition (Fig. 2E); however, the situation was opposite under low preload (Fig. 2F). This indicated that reducing the preload-controlled water film between HMSAMS cap brims and the substrate could significantly enhance tangential adhesion (detailed explanation is presented in the following text). In addition, under a preload of 0.4 kPa, the tangential adhesion-to-preload ratio in underwater and dry environments remained at 12.9 ± 0.75 and 182 ± 4.1, respectively; when the preload increased to 8 from 0.4 kPa, the ratios started to decrease (Fig. 2F). Nevertheless, the minimum tangential adhesion-to-preload ratio still exceeded 8, which was attributed to the joint function of preload-induced deformation of the hollow cavities and the intimate contact of the HMSAMS double cap brims with the substrate during the attachment-detachment process. Up to now, few microstructures have exhibited such high-performance tangential adhesion and superior tangential adhesion-to-preload ratios underwater (tables S1 and 2) (*24*, *25*, *29*–*38*). Due to the advantages of the HMSAMS patch, different objects could be conveniently grasped under both dry [glass ball (4 g), Teflon block (1200 g), glass block (146 g), and copper plate (82 g) in Fig. 2E and fig. S7; movie S3] and underwater [alloy plate (872 g), weight (1000 g), and Teflon block (303 g) in Fig. 2E and fig. S8; movie S4] conditions (text S3).

According to the experimental results on different materials (Fig. 2G), normal and tangential adhesion in dry and underwater environments on Si and plastic surfaces [polyethylene terephthalate (PET) and polyimide (PI)] was above 100 kPa. On steel or even super-hydrophobic surfaces (Teflon), their values were always >50 kPa, revealing to a certain extent that intermolecular forces and negative pressure effect could universally enhance HMSAMS adhesion to various materials.

Ultimately, the effects of interfacial water molecules between PDMS film and Si substrate were investigated through molecular dynamics (MD) simulations (Fig. 2H), with methodological details provided in Materials and Methods. When the spacing distance between PDMS film and Si substrate increased from 0 to 10 Å at an interval of 2 Å [Fig. 2H (i)], normal and tangential adhesion generally decreased in both dry and underwater environments on account of the decrease in intermolecular attraction [Fig. 2H (ii)]. Notably, because the diameter of water molecule was larger than the spacing distance, the normal adhesion underwater at 2 Å presented an anomalous singularity (i.e., the value suddenly decreased). However, under the same spacing, the adhesion in the normal and tangential directions underwater was higher than that in dry condition [Fig. 2H (ii)], again verifying that thin water molecule film could improve adhesion. Actually, the relative humidity was not exceeding 90% when the thickness of water layers was thinner than 20 Å (*40*). Under this condition, the total adhesion force in simulations could also be calculated by following theoretical formula (*41*)

$$F=\left[\frac{\left(1-\rho \right)A_{\text{dry}}}{6\pi D_{0}^{3}}+\frac{\rho A_{\text{wet}}}{6\pi D_{0}^{3}}+\frac{\rho A}{6\pi h^{3}}\right]b_{1}b_{2}$$ (1)

where ρ is the relative humidity, $A_{\text{wet}}$ is the Hamaker constant with intervening water layers, $A_{\text{dry}}$ is Hamaker constant without water layer between the two solid surfaces, *A* is Hamaker constant between water molecules and the solid substrate, $D_{0}$ is surface separation (i.e., the thickness of water layers *h*), $b_{1}$ is the length of PDMS film, and $b_{2}$ is width of PDMS film. In above Eq. 1, van der Waals force between the PDMS film and Si substrate seems to be weakening by the intermediate water layer. However, the strong attractive force between the water molecules and solid surface may compensate the reduced interaction and enhance the adhesion of two solid surfaces (*41*).

Contrary to the conventional phenomena where water film weakened adhesion, the proposed HMSAMS patch demonstrated an anomalous water-film–enhanced adhesion effect under both normal (preload, >4 kPa) and tangential (preload, >10 kPa) loading conditions, consistent with the above theoretical predictions and simulations. Meanwhile, a detailed experimental comparison was performed to investigate the water-film–enhanced adhesion effect via analyzing the traction-separation curves of the HMSAMS patch measured under vacuum conditions (fig. S9A). Thereinto, the layer between the PDMS film and the Si substrate was ideally considered as a cohesive layer, and the slope of the traction-separation curve (*k*_1_ and *k*_2_) represented its interface stiffness. Under identical preload conditions (preload, 100 kPa), the slope of the traction-separation curve in the dry condition (*k*_2_) was significantly lower than that observed underwater (*k*_1_), suggesting that the presence of water film notably enhanced the cohesive layer and increased its interface stiffness, respectively. Consequently, the underwater adhesion force, represented by the peak value of the traction-separation curve, exhibited a marked increase compared to its dry counterpart. Meanwhile, underwater conditions exhibited obviously higher fracture energy compared to dry environments under equivalent preloads, as evidenced by the increased area under the traction-separation curve. This phenomenon indicated that the presence of water molecules between the PDMS and Si surfaces substantially enhanced the energy dissipation during detachment. Moreover, the water-film–enhanced interface stiffness, adhesion force, and fracture energy under different preloads were also proved (fig. S9, B and C, and text S4). Therefore, the structural morphology of HMSAMS and preload might jointly facilitate the water film to enhance adhesion.

### Peeling-resistant and contact-adaptable performance of robot track

**Fig. 3.**
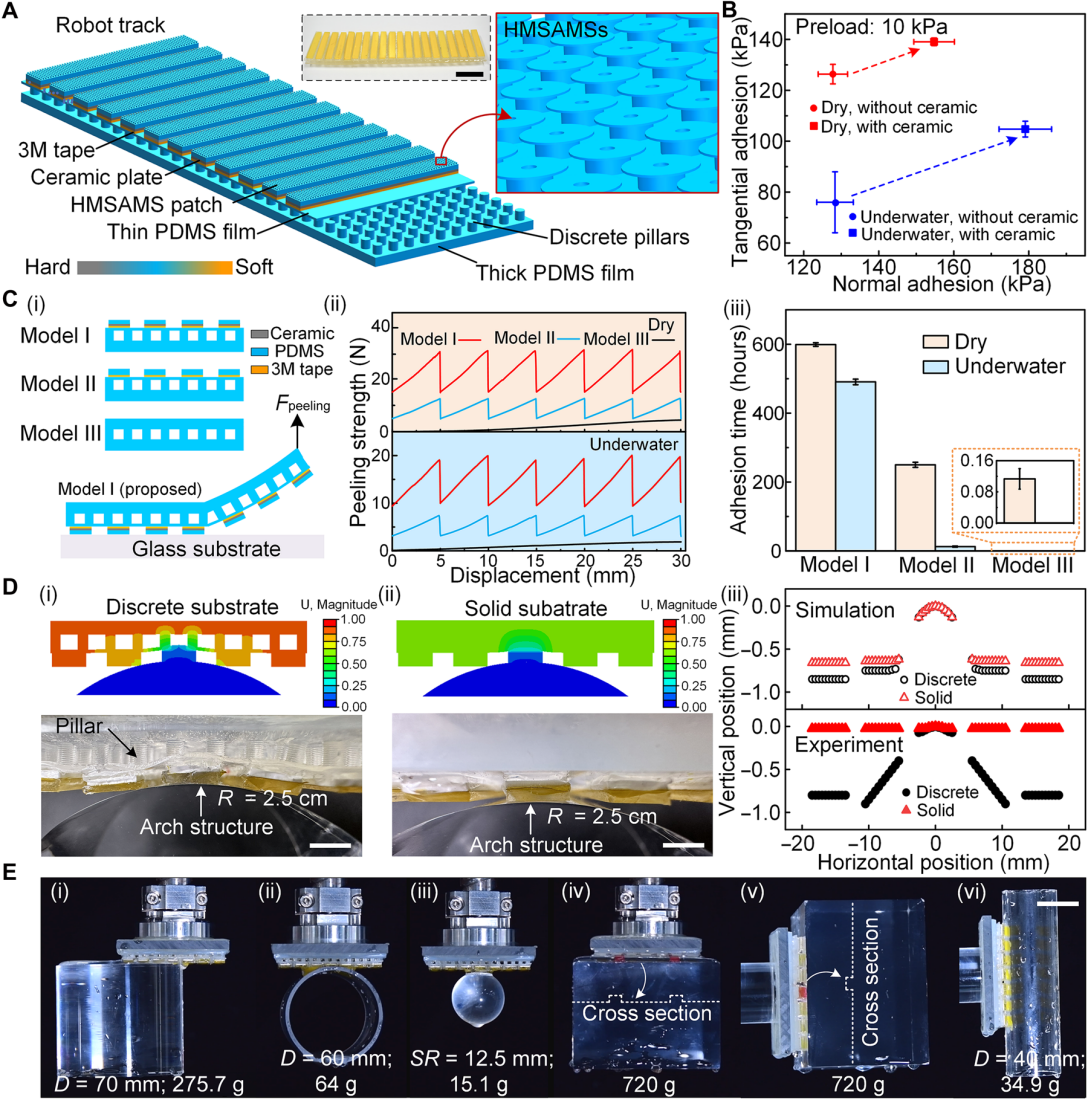
Macroscopic hierarchical structure, adhesion performance, and contact adaptability of robot track. (**A**) Macrostructure of the proposed all-in-one robot track. The flexible substrate consists of thick and thin PDMS films separated by discrete pillars. On the top of substrate, a discrete core-shell HMSAMS patch is formed by the adhesive layer, ceramic plate, and 3M tape. Scale bar, 3 cm. (**B**) Comparison of tangential and normal adhesion strength of HMSAMS patch with and without ceramic plate in dry and underwater environments (preload, 10 kPa). (**C**) Comparison of (ii) peeling strength and (iii) adhesion time of (i) three different robot track types (model I, model II, and model III) in dry and underwater environments. Thereinto, the three models (i) were horizontally pressed on the glass substrate under the preload of 7.8 kPa, and a vertical peeling force (*F*_peeling_) was applied to one free end of the models. Meanwhile, in durability testing (iii), one end of robot track was horizontally attached to negative surface of glass plate, while a 0.5-kg weight was suspended vertically from the other end. (**D**) Contact adaptability of robot track with and without discrete pillars against a semicylindrical surface (radius, 2.5 cm) at a given preload (simulation, 1.5 N; and experiment, 5 N). Scale bars, 4 cm. (**E**) Demonstration of normal and tangential adhesion performance of robot track when grasping different objects from water. Scale bar, 2 cm.

Beyond the high-strengthen adhesion of HMSAMS patch, excellent peeling resistance is critical for low-power, long-term attachment of WCR especially under loaded conditions, which poses a tremendous challenge for soft materials due to their inherent stress relaxation and interface stress concentration. Additionally, achieving universal adaptability to unstructured terrains (i.e., overcoming the limitation of crawling only on specific surfaces) can significantly reduce WCR’s constraints on crawling surface topography. Hence, respectively mimicking the discrete behavior of adhesive structures and high-module property of bones or cartilages on gecko soles and octopus tentacles, mechanically isolated HMSAMS patches embedded with rigid cores were arranged in parallel on the substrate surface of robot tracks at given intervals to suppress interface crack propagation and enhance adhesion strength (or improve peeling threshold). Meanwhile, replicating the low-module function of muscle on their soles and tentacles to improve conformal contact on crawling surfaces, discrete pillars were incorporated into the track substrate to reduce the effective elastic modulus and improve contact adaptability to target surfaces. Thus, peeling-resistant and contact-adaptable robot tracks were engineered (Fig. 3A), endowing the WCRs with stable movement on both smooth and uneven vertical surfaces and long-time attachment in multimedia environments.

Quantitative comparison of the normal and tangential adhesion forces of HMSAMS patches with and without rigid cores (i.e., ceramic plate) was first conducted under a 10-kPa preload in dry and underwater environments (Fig. 3B). Thereinto, the normal adhesion of the HMSAMS patch with embedded rigid core increased significantly from 128 ± 4.5 to 155 ± 6 kPa in dry environment and from 128 ± 5 to 179 ± 7 kPa underwater, while tangential adhesion increased from 126 ± 4 to 138.5 ± 2 kPa in dry environment and from 76 ± 12 to 104.5 ± 3 kPa underwater. The significant improvement in adhesion strength under both dry (normal, 21%; and tangential, 9.5%) and underwater (normal, 39.8%; and tangential, 36.8%) conditions was attributed to the rigid cores that reshaped interface stress distribution and suppressed crack initiation and propagation. The simulation results comparing rigid-core–enhanced adhesion and corresponding detachment process are presented in fig. S10 and text S5.

The peeling strength of the proposed robot track (model I) and control samples (model II, without rigid core; and model III, with a continuous adhesive layer) was investigated in depth by vertically detaching them from horizontal glass surface [Fig. 3C (i)]. During peeling, the peeling force of model I oscillated in both dry and underwater environments as it passed each discrete core-shell HMSAMS patch, whose peak values were respectively maintained at ~31 N (dry) and ~20 N (underwater) [Fig. 3C (ii)]. A similar variation trend of peeling force was exhibited by model II, with the peak force maintained at around 13 N (dry) and 8 N (underwater) [Fig. 3C (ii)]. Because of the existence of rigid cores in the HMSAMS patches, the peak force of model I compared to model II evidently improved by 138 and 150% in dry and underwater environments, respectively. On the contrary, the peeling force of model III increased progressively to 5.2 N (dry) and 3 N (underwater) [Fig. 3C (ii)], which were significantly lower than the values of model I and model II. During vertical peeling process (i.e., the process of crack propagation accompanied by energy release), model III could not effectively restrict crack propagation due to the absence of rigid core and heterogeneous design in adhesive layer, jointly leading to the slow increase of its peeling force to smallest peak. In contrast to model III, models II and I dispersed interfacial stress across multiple discrete structural interfaces, and the discrete structures effectively prevented catastrophic crack propagation triggered by the interfacial stress concentration and enhanced adhesion properties (*42*, *43*). Meanwhile, the discrete high-modulus ceramic plates in HMSAMS patches not only improved the bending stiffness of model I during peeling but also realized crack-arresting function (*44*, *45*), which brought about significantly enhanced peeling force compared with the stead-state peeling force of model III. The aforementioned arrangements jointly enhanced resistance to peeling, further improving the peeling force of model I. Notably, the peeling operation stalled when the applied force fell below the peak force, demonstrating that discrete, core-enhanced adhesive layers (versus continuous ones) effectively increase the peeling threshold. Due to the high peeling threshold, the proposed robot track (model I) maintained attachment over 500 hours in both dry and underwater environments by hanging a 500-g weight, i.e., substantially longer than models II and III [Fig. 3C (iii)].

Apart from the peeling strength, the contact adaptability of robot track was evaluated through both simulations and experiments (Fig. 3D). A key difference emerged: The proposed robot track featured a discrete substrate (pillar arrays sandwiched between the upper and bottom films), while the control sample harnessed a solid substrate. Specifically, under identical preloads, the proposed robot track [Fig. 3D (i)] exhibited a deeper indentation (simulation, 2.4 mm; and experiment, 0.9 mm) with the semicylindrical surface than the control sample [simulation, 0.56 mm; and experiment, 0.025 mm; Fig. 3D (ii)]. In other words, the indentation depth of the proposed discrete substrate was ~36 times greater than that of the control sample in experiments and 4.3 times greater in simulations, both of which primarily resulted from the significantly reduced effective modulus of the substrate due to the presence of discrete pillars [the selected two-dimensional (2D) simulation model rather than 3D one might be the main reason for the inconsistency between the experimental results and the simulation results]. Simulations further confirmed this excellent contact adaptability across different preloads (figs. S1 and S11 and text S6).

To demonstrate the robust adhesion performance and contact adaptability of the proposed robot track, objects with different shapes, weights, sizes, and surface morphologies were grasped and transferred from water into air (Fig. 3E). For instance, the robot track was able to vertically grasp (normal adhesion ability) the end face of a cylinder [275.7 g; Fig. 3E (i)] with nearly one half of track surface area engaged, as well as cylindrical [64 g; Fig. 3E (ii)] and spherical surfaces [5.1 g; Fig. 3E (iii)] with smaller contact area. It could also conformally attach to objects with convex and concave surfaces [720 g; Fig. 3E (iv)], grabbing them out of the water (fig. S12 and movie S5). Meanwhile, as regards the tangential adhesion performance, the robot track could firmly attach to uneven [720 g; Fig. 3E (v)] and cylindrical surfaces [34.9 g; Fig. 3E (vi)] via conformal or partial contact, respectively, effectively moving them from water to air (fig. S12 and movie S5). Corresponding operations in both tangential and normal directions were also demonstrated under dry condition (fig. S13 and movie S6). Moreover, the insensitivity of adhesion performance to materials was successfully verified by robot tracks under different conditions [normal: figs. S14 (dry) and S15 (underwater); and tangential: figs. S16 (dry) and S17 (underwater), corresponding movies S7 to S10 and text S7].

### Cross-medium WCR and its multi- and cross-medium adhesion performance

**Fig. 4.**
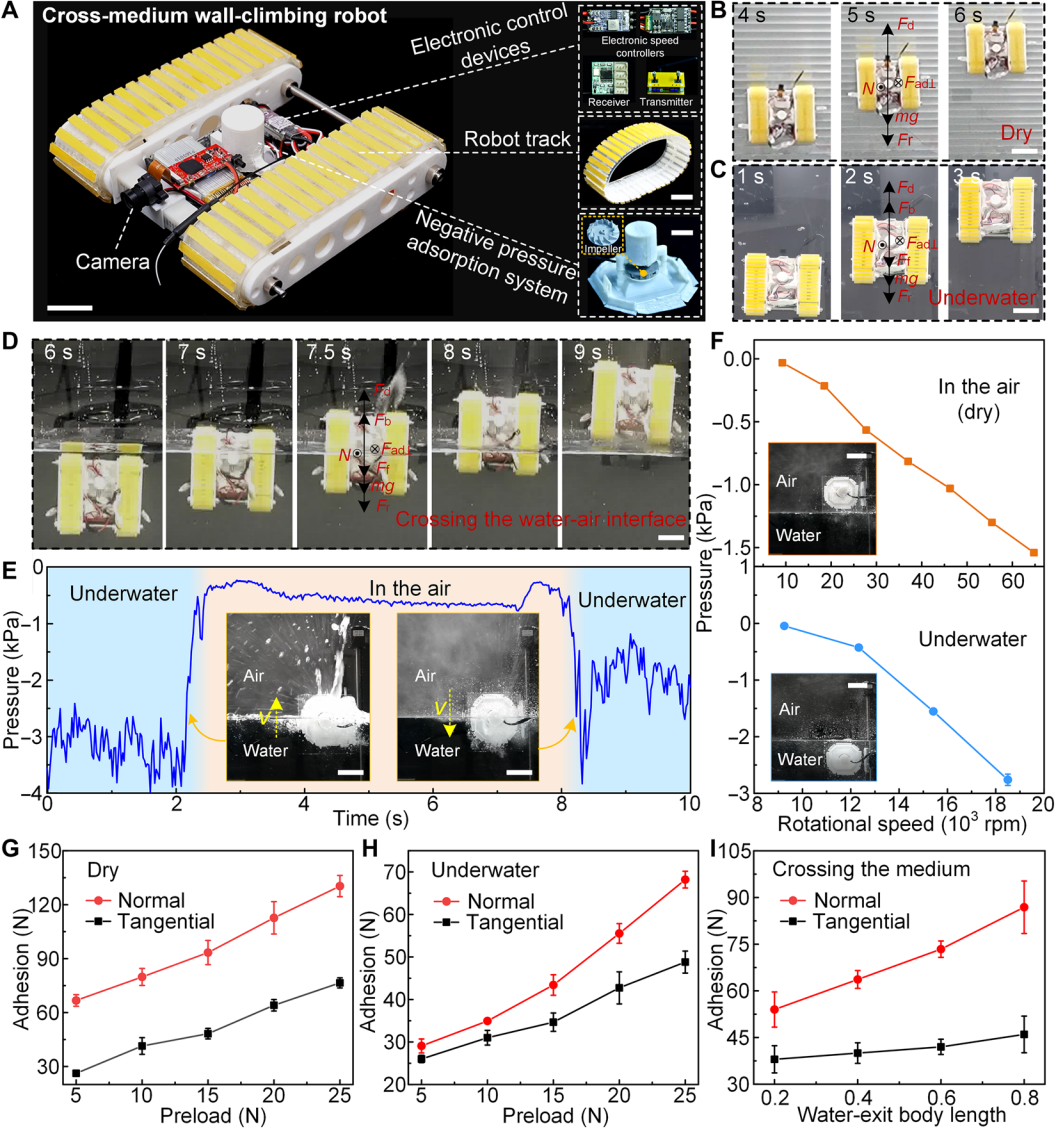
Multi- and cross-medium adhesion performance of the cross-medium WCR. (**A**) Photograph depicting the structure and major components of the cross-medium WCR. Scale bars, 2 cm. (**B** and **C**) WCR crawling on a vertical surface in (B) dry and (C) underwater environments at different time points, where $F_{d}$ , $F_{b}$ , $F_{f}$ , N , m , g , $F_{\text{ad}⊥}$ , and $F_{r}$ represent the driving force, buoyancy force, fluid resistance, support force, mass, gravitational acceleration, normal adhesion forces, and rolling resistance of the WCR, respectively. Scale bars, 5 cm. (**D**) Snapshots of the WCR crossing the water-air interface. Scale bar, 5 cm. (**E**) Representative pressure-time profile of the NPAS when crossing the water-air interface at a given speed. The insets demonstrate the NPAS crossing the water-to-air and air-to-water interfaces, respectively. Scale bars, 3 cm. (**F**) Pressure of the NPAS at different rotational speeds in the air and underwater. The insets show the dry and underwater experimental environments. Scale bars, 3 cm. (**G** and **H**) Normal and tangential adhesion forces of WCR under different preloads in (G) dry and (H) underwater environments. (**I**) Normal and tangential adhesion force of WCR at different water-exit body lengths under a preload of 15 N. Water-exit body length of a robot is defined as the ratio of the actual water-exit length to the total length.

Leveraging the dry/water-tolerant nature of the proposed HMSAMSs, as well as the superior peeling resistance and contact adaptability of the robot tracks, a custom-made amphibious cross-medium WCR was constructed [Fig. 4A and fig. S18 (after waterproof processing); overall weight, 485 g], which mainly comprised several electronic control devices (electronic speed controllers, receiver/transmitter, and control handle), a negative pressure adsorption system (NPAS; impeller and water-proof motor), and various 3D printed components (fig. S19 and text S8). Thereinto, the NPAS, installed at the center of the robot’s body that were constructed from 3D printed components, dynamically adjusted the motor speed to provide on-demand preload to the robot’s tracks; simple electronic control devices enabled WCR’s untethered movements (e.g., straight-line motion and turning). A detailed frame of the robot control hardware is illustrated in fig. S20. Assisted by the cross-medium HMSAMS array on the robot tracks, the proposed WCR was capable of robust crawling ability on vertical surfaces in dry environment (Fig. 4B and movie S11) and underwater (Fig. 4C and movie S12), as well as smoothly crossing the water-air interface (Fig. 4D and movie S13). In single medium, the proposed robot realized stable and smooth motion on vertical walls at a given speed, due to that the WCR force balance was well maintained in dry environment ( $N=F_{\text{ad}⊥}$ , $F_{d}=\mathrm{mg}+F_{r}$ ) and underwater ( $N=F_{\text{ad}⊥}$ , $F_{d}+F_{b}=F_{f}+\mathrm{mg}+F_{r}$ ), where $F_{d}$ , $F_{b}$ , $F_{f}$ , N , m , g , $F_{\text{ad}⊥}$ , and $F_{r}$ represent the driving force, buoyancy force, fluid resistance, support force, mass, gravitational acceleration, normal adhesion forces, and rolling resistance of the WCR (ideally, NPAS pressure was entirely applied to the robot's tracks).

Nevertheless, complex cross-medium transition conditions (e.g., the abrupt or continuous changes in fluid density, interface humidity/dryness, and bearing forces) tended to trigger crawling speed fluctuation and water-exit failure. For instance, the decreasing buoyancy force ( $F_{b}$ ) and speed-dependent fluid resistance ( $F_{f}$ ) were more inclined to disrupt the force balance during water-exit process. Therefore, at this stage, maintaining relative balance of forces was prerequisite for the smooth cross-medium movement of WCR, whose corresponding force balance equations can be written as $N=F_{\text{ad}⊥}$ and $F_{d}+F_{b}-F_{f}-\mathrm{mg}-F_{r}=\mathrm{ma}$ , where *m* and *a* represent robot mass and acceleration, respectively (the added mass force, surface tension, and capillary forces were ignored). According to the equations, improving the normal adhesion force can effectively prevent robot detachment from a surface, while increasing the maximum static friction force, i.e., enhancing the tangential adhesion force, can strongly prevent slipping. On the contrary, without an HMSAMS array on the robot tracks (i.e., no adequate normal and tangential adhesion forces), water-exit failure occurred—slipping (fig. S21 and movie S14).

Because the adhesion force magnitude of the robot tracks was predominantly controlled by NPAS preload, the corresponding time-dependent NPAS pressure curve during water-exit and water-entry processes (fig. S22 and movie S15) was recorded (Fig. 4E). The NPAS pressure underwater was maintained at ~−3 kPa and instantly increased to ~−0.5 kPa during water-to-air transition process, which was mainly attributed to the significantly lower density of air compared to that of water. While suspended in the air, the pressure decreased slightly to ~−0.8 kPa, probably due to increased motor rotational speed after the impeller shed water droplets. Subsequently, the pressure increased first abruptly to ~−0.25 kPa (on account of water resistance on the motor) and then decreased sharply to −3.8 kPa before reaching stability at ~−2.5 kPa as the NPAS entered water at constant speed. Meanwhile, NPAS pressure was quantitatively characterized under different rotational speeds in dry and underwater environments (Fig. 4F). In dry condition, the pressure in the NPAS cavity decreased almost linearly from −0.32 ± 0.004 to −1.54 ± 0.016 kPa with increasing rotational speed (from 9000 to 65,000 rpm); underwater, it decreased from −0.045 ± 0.004 to −2.76 ± 0.1 kPa when the rotational speed increased from 9000 to 18,500 rpm. The pressure exhibited significant differences between the dry and underwater environments, necessitating a broad adjustment range for the motor rotational speed to output optimal preload during the cross-medium transitions. Notably, the maximum negative pressures achieved by the NPAS under dry (1.54 kPa) and underwater (2.76 kPa) conditions were ~1/156th and 1/105th, respectively, of the maximum normal adhesion force of the HMSAMS patch under corresponding conditions, which were substantially lower than those observed in traditional negative-pressure adsorption WCRs (*46*). Furthermore, the NPAS-generated maximum preload (dry, 2.65 N; and underwater, 4.75 N) fell below the weight of the cross-medium WCR (4.85 N), underscoring the critical role of high-performance cross-medium adhesion in enabling vertical water-exit movements for the WCR.

Therefore, the normal and tangential adhesion forces of WCR in dry and underwater environments under different preloads were quantitatively measured (Fig. 4, G and H). In dry condition (Fig. 4G), the adhesion force increased almost linearly in the normal (from 66.7 ± 3.2 to 130.3 ± 5.9 N) and tangential (from 26.2 ± 1.4 to 76.5 ± 2.8 N) directions, when the preload was increased from 5 to 25 N with 5-N increments. Underwater (Fig. 4H), the adhesion force varied between 29.1 ± 1.6 and 68.2 ± 1.98 N in the normal direction and between 26 ± 0.86 and 48.8 ± 2.6 N in the tangential direction with increasing preload. The WCR’s dynamic and adjustable adhesion force not only maintained optimal force balance at the water-air interface but also enhanced crawling efficiency while minimizing energy consumption. Based on the robot tracks’ strong dry and underwater adhesion, the cross-medium adhesion performance of WCR under different water-exit body lengths was quantitatively measured (Fig. 4I). With the increase of the water-exit body length from 0.2 to 0.8, the adhesion force increased from 54 ± 5.6 to 86.8 ± 8.4 N in the normal direction and from 38 ± 4.4 to 46 ± 5.9 N in the tangential direction. According to the variation trends, adhesion force was relatively weak when the robot first emerged from water. Nevertheless, the robot track’s high-performance adhesion, far exceeding the robot’s weight, effectively mitigated slipping and overturning risks caused by continuously decreasing buoyancy and increasing fluid resistance during the transitions. Consequently, a simple open-loop control strategy (fig. S19), rather than a complex closed-loop system adopted in previous studies (*47*), was implemented in the WCR to ensure smooth water-to-air transitions, which also brought about tremendous advantages for proposed WCR: radiation-hardened operation by removing vulnerable sensors, cost-effective deployment in large-scale radioactive scenarios, and reliable water-to-air transitions through predictable actuation sequences.

### Wall-climbing performance of WCR in multi- and cross-medium environments

**Fig. 5.**
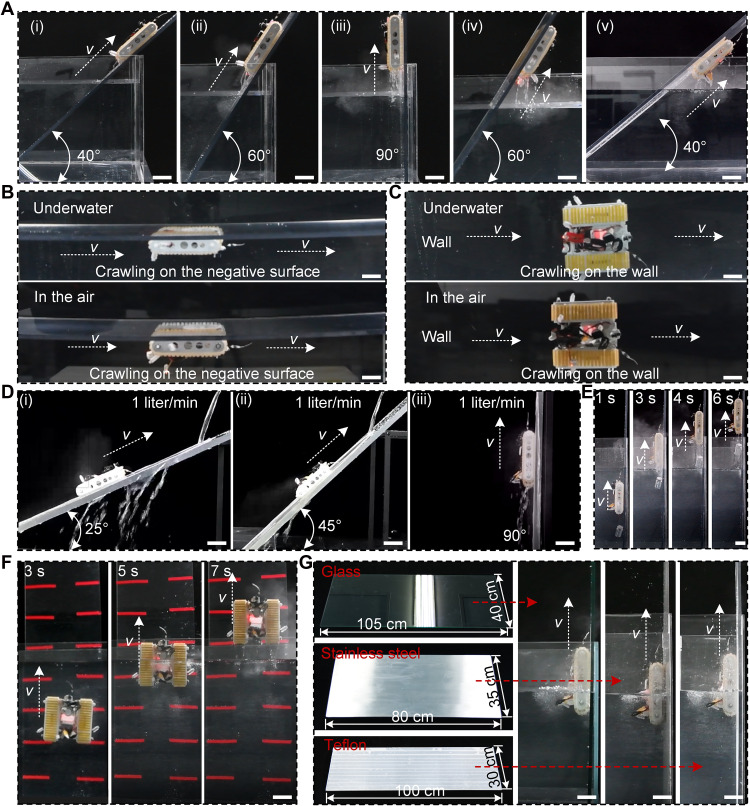
Multi- and trans-medium wall-climbing performance of the cross-medium WCR. (**A**) Water-exit ability of WCR on positive and negative surfaces at varying inclination angles (40°, 60°, and 90°). (**B** and **C**) Wall-climbing performance of WCR on (B) negative surfaces and (C) side walls in the air and underwater. (**D**) Wall-climbing ability of WCR on inclined surfaces [(i) 25°, (ii) 45°, and (iii) 90°] against the water at a given flow rate (1 liter/min). (**E**) Load-bearing capacity of WCR during water-exit process (weight, 300 g). (**F**) Contact adaptability of WCR on uneven surfaces (3M tape thickness, 0.5 mm). (**G**) Wall-climbing ability of WCR on different material surfaces (glass, stainless steel, and Teflon). Scale bars, 5 cm.

The outstanding adhesion performance and contact adaptability of the robot tracks in multi- and cross-medium environments endow the WCR with stable crawling ability under complex conditions. In particular, the WCR rapidly traversed the water-air interface from the positive and negative surfaces at different inclination angles (40°, 60°, and 90°) without slipping and tipping (Fig. 5A and movie S16), demonstrating robust adaptability to inclined surfaces. Furthermore, the proposed WCR also achieved trans-medium water-entry movement on both positive and negative surfaces (fig. S23, movie S17, and text S9). In the aforementioned scenarios, the support force of the WCR, determined by the combination of the vertical component of gravity, buoyancy force, and normal adhesion (fig. S24 and text S10), was larger than zero (i.e., N≥0 ). Particularly, when the WCR was positioned on a negatively inclined surface, stronger normal adhesion was required to counteract the gravitational force component, ensuring that the supporting force remained positive. Otherwise, slipping or overturning was more likely to occur. Therefore, the antislipping and antioverturning conditions of the WCR on negative surface (fig. S25) were calculated by following equations (ideally, each core-shell HMSAMS patch unit would exhibit identical normal adhesion, and the robot track would transmit no torque) (*48*)

$$F_{\text{normal adhesion}}\geq \frac{G\text{cosθ}}{2n}+\frac{G\text{sinθ}}{2n\mu }\;\left(\text{antislipping}\right)$$ (2)

$$F_{\text{normal adhesion}}\geq \frac{G\left(l_{c}\text{cosθ}+H\text{sinθ}\right)}{2L}\;\left(\text{antioverturning}\right)$$ (3)

where G represents gravitational force, $F_{\text{normal adhesion}}$ is the normal adhesion of a single core-shell HMSAMS patch, H is the distance between the robot center of gravity and substrate surface, *n* is the number of core-shell HMSAMS patches in contact with the substrate surface, $l_{c}$ is the distance between the overturning point and the WCR gravity center parallel to the substrate plate, μ is the friction coefficient, *L* is the contact length of robot track with substrate surface, and θ is the angle between substrate and horizontal plane (texts S11 and S12). According to Eqs. 2 and 3, sufficient normal adhesion and a high static friction coefficient in the core-shell HMSAMS patches could guarantee stable attachment of the WCR to negative surfaces. Notably, during the 4-hour durability testing (WCR statically attached to a 45° negative surface), no slipping and overturning occurred.

Due to the strong dynamic and static adhesion of the robot tracks, the WCR successfully climbed on negative surfaces (Fig. 5B and movie S18) in both dry and underwater environments, which vividly demonstrated that the weight of the WCR was wholly offset by normal adhesion in air and by the combined buoyancy and normal adhesion underwater (fig. S24 and text S10). Notably, the WCR could also crawl horizontally along sidewalls without slipping in dry and underwater conditions, as its tangential adhesion in the vertical direction fully or partially counteracted its own weight (Fig. 5C, fig. S24, movie S19, and text S10). The aforementioned phenomena can be principally attributed to the high adhesion strength of the HMSAMS patches in different media. In other words, despite extremely low pressure provided by NPAS, the robot tracks act as function amplifiers, significantly strengthening the normal and tangential adhesion performance of the WCR across all conditions.

Crawling against currents and carrying heavy objects on vertical surfaces also pose formidable challenges to the crawling ability of WCR in wet environments. However, the proposed WCR still demonstrated robust movement against a water flow of 1 liter/min on inclined PMMA plates {inclination angle: 25° [Fig. 5D (i)], 45° [Fig. 5D (ii)], and 90° [Fig. 5D (iii)]; corresponding movie S20} and superior climbing performance while carrying a 300-g weight across the water-air interface (Fig. 5E and movie S21), whose obstacle-surmounting ability might supercharge its application in complex and hostile environments. The differential drive motors endowed the WCR with a flexible steering function (fig. S26 and movie S22), enabling it to flexibly adjust its posture in real time during mission operations.

The self-adaptive capability of the robot tracks to uneven terrains facilitated WCR’s seamless water-to-air transitions on highly irregular surface (PMMA plate with discrete 0.5-mm-thick 3M tape stripes; Fig. 5F and movie S23). Even on pebble-covered rough surfaces, the WCR, like some traditional track-based robots, could maintain stable crawling ability harnessing its discrete configuration on robot tracks (fig. S27 and movie S24). Furthermore, wall-climbing ability remained robust regardless of the material types (glass, stainless steel, and Teflon), because the universality of HMSAMS’s adhesion to materials allowed WCR’s smooth water-to-air transitions (Fig. 5G and movie S25). Stable and seamless cross-medium crawling of the proposed WCR on different material surfaces and terrain profiles, which had not yet been realized or reported before this, mainly ascribed to the hierarchically structural design of robot tracks and its high-performance cross-medium adhesion.

### Field applications of cross-medium WCR

**Fig. 6.**
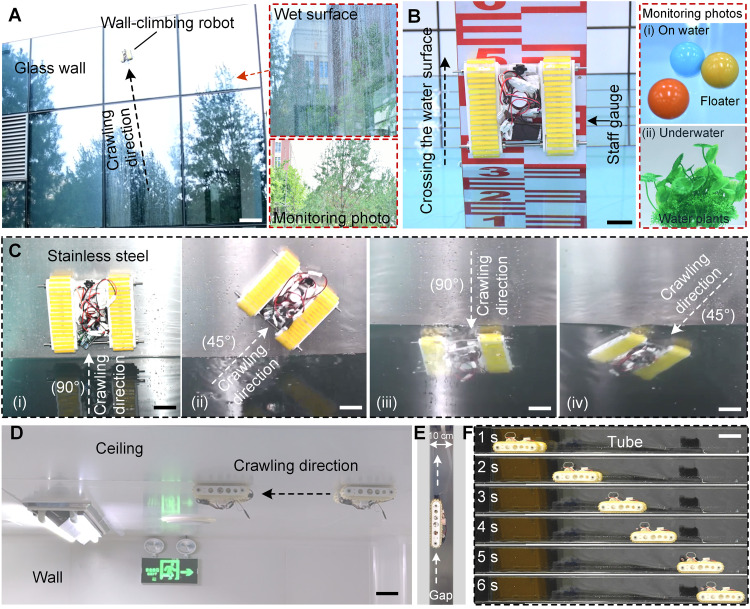
Field applications of the cross-medium WCR. (**A**) Stable crawling of WCR on a droplet-attached surface of an outdoor building. Scale bar, 20 cm. (**B**) Cross-medium crawling of WCR on the surface of a staff gauge. Scale bar, 4 cm. (**C**) WCR’s water-exit (i and ii) and water-entry (iii and iv) movements in different directions (90° and 45°) in a stainless steel pool. Scale bars, 4 cm. (**D**) Demonstration of WCR crawling on an indoor ceiling. Scale bar, 5 cm. (**E**) Vertically crawling of WCR in a narrow gap (width, 10 cm). (**F**) Horizontally crawling of WCR in a slender tube. Scale bar, 10 cm.

Because of the cross-medium, peeling-resistant and contact-adaptable performances of the proposed WCR, multi- and trans-medium wall-climbing movements (especially on both negative and vertical surfaces of different materials and terrain profiles) were also expeditiously executed by WCR in its potential application scenarios. For instance, disregarding the difference between the water and air media, the proposed robot nimbly climbed a vertical glass wall covered with remnant water droplets from bottom to top by adjusting preload while recording the surroundings with its onboard camera (Fig. 6A and movie S26). Notably, on account of the sustained zero-power wet-surface adhesion capacity of robot tracks, the robot could lastingly monitor the surrounding environment from building vantage points in all weather conditions without on-board energy consumption. Meanwhile, in certain ponds, rivers, or lakes, the WCR could hide underwater (especially in opaque water) to avoid detection and then emerge along staff gauges or vertical walls when needed to observe surface conditions (Fig. 6B and movie S27), which significantly reduced detection risk. Objects floating on the water surface and underwater aquatic plants could be monitored by the miniature camera carried by the WCR. Furthermore, on certain nonmagnetic smooth vertical walls, e.g., stainless steel sink filled with heavy water at nuclear power plants, the WCR could reversibly cross the water-air interface from different directions (45° and 90°) with ease (Fig. 6C and movie S28). Hence, it could potentially substitute humans in performing hazardous tasks in radioactive environments. In addition, during humid weather [e.g., “Hui Nan Tian,” a phenomenon caused by warm and moist southern airflows in spring (in China)] or prolonged rainfall, the WCR could firmly climb moist ceilings while monitoring indoor areas from concealed vantage points (Fig. 6D and movie S29). Most critically, in certain areas of nuclear power plants or in slender tubes within chemical equipment, physical constraints prevent human entry, making robots the only straightforward and effective solution for task execution. The proposed untethered WCR, with its relatively small dimensions (18 cm by 16 cm by 5 cm), successfully navigated narrow vertical gaps (width, 10 cm; Fig. 6E and movie S30) and slender tubes (height, 10 cm; Fig. 6F and movie S31), providing an alternative for performing space-confined tasks. Despite the notable differences in crawling material [e.g., glass (Fig. 6A), paint (Fig. 6B), stainless steel (Fig. 6C), iron (Fig. 6, D and E), and plastic (Fig. 6F)], environmental condition [wet (Fig. 6A), underwater (Fig. 6, B and C), moist (Fig. 6D), and dry (Fig. 6, E and F)], and surface roughness across aforementioned scenarios, the cross-medium WCR simultaneously traversed such vertical and inverted scenes, an insurmountable challenge for conventional crawling robots, exhibiting superior versatility and adaptability.

## DISCUSSION

Inspired by the morphology of adhesive structures and their macroscopical discrete behavior on gecko toes and octopus tentacles, as well as the functional roles of bones (or cartilage) and muscles, cross-medium WCR integrating dry/underwater-tolerated HMSAMSs on mechanically discrete, rigid-soft hybrid hierarchical robot tracks have been proposed, simultaneously achieving high-performance adhesion and contact adaptability on diverse material surfaces in multimedia environments. Thereinto, HMSAMSs combine the structural morphology of gecko-inspired mushroom-shaped dry adhesive microstructures and the hollow cavities of octopus suction cups, realizing excellent normal and tangential adhesion performance in both dry and underwater environments. By adding mechanically discrete core-shell HMSAMS patches on the top layer of robot tracks (i.e., simulate the discrete feature of adhesive structures on gecko toes and octopus tentacles), interface crack propagation is effectively suppressed. Meanwhile, mimicking the high-modulus function of bones in gecko toes and cartilage in octopus tentacles, the rigid cores adopted in discrete HMSAMS patches significantly improve the peeling threshold. Furthermore, the discrete pillar arrays at the bottom layer of the robot tracks, corresponding to the soft muscle function, significantly reduce the effective modulus and enhance contact adaptability. On account of the aforementioned ingenious all-in-one structures and their advantageous characteristics, the cross-medium WCR can expeditiously crawl on positive and inverted surfaces of varying inclination angles in both dry and underwater environments or even cross the air-water interface effortlessly on even/uneven or undulating surfaces of different materials.

Primarily benefitting from the intermolecular forces between the double cap brims and the contact surface, as well as the negative-pressure effect of the hollow cavities, the proposed HMSAMSs exhibit excellent normal (dry, 240 kPa; and underwater, 290 kPa) and tangential (dry, 108.13 kPa; and underwater, 127.3 kPa) adhesion performance, far superior than previously reported PDMS-based adhesive structures (table S1) (*24*, *25*, *29*–*39*). Although some adhesion performance indicators (e.g., normal or tangential adhesion in dry environment) of the adhesive structures based on hydrogels (*49*, *50*), shape-memory polymers (*51*, *52*), copolymer (*53*–*55*), coacervate (*56*), and polyurethane (*57*, *58*) are better than those of the PDMS-based HMSAMS patch (tables S3 and S4), the time required for generating high adhesion performance under external stimulation is generally longer or the preload is relatively huge. On the contrary, the adhesion performance of HMSAMS patch has always persisted without external stimulation, and it did not experience performance regression in 18 months (fig. S28). Meanwhile, the slightly angled ultrathin double cap brims of HMSAMSs enable intimate contact with object surfaces under minimal preload while forming self-sealing cavities to prevent detachment (*58*–*60*). This significantly improves the adhesion-to-preload ratio in both dry (normal, 792; and tangential, 182) and underwater (normal, 57; and tangential, 12.9) environments, surpassing most values reported in previous studies (table S2) (*24*, *25*, *29*–*39*), which brings about friendly contact ability under low preload especially for fragile surfaces, excellent dynamic attaching stability on object surfaces, and low-energy operation for untethered robots (fig. S29, movie S32, and text S13). The deformation of the HMSAMS cap brims in the self-sealing cavity during the detachment process could be directly observed in the simulation results (dry, fig. S30; and underwater, fig. S31; text S14), and the variation trend of adhesion strength with preload (atmospheric pressure, 0.101 MPa) observed in the simulation is also consistent with the experimental results (figs. S32 and S33 and text S15). The underwater adhesion strength exceeds that in air under identical preload, indicating that the water film between HMSAMS cap brims and contact surface can effectively enhance adhesion strength (fig. S8). This phenomenon also agrees with previous experimental (*58*), theoretical (*41*), and MD simulation results (Fig. 2H). Furthermore, strong insensitivity of adhesion to diverse materials (figs. S14 to S17), scalable adhesive mechanism (i.e., proportional relationship of adhesion force to measured area) (fig. S34), and adjustable adhesion performance (figs. S3 and S6) of HMSAMSs were also confirmed according to adhesion strength measurements or demonstrations. Although the adhesion strength of HMSAMS on the highly soft (sponge substrate, fig. S35), rough (Ra, 0.8 to 1.6 μm; figs. S36 and S37), and dusty (<0.01 g/ml, fig. S38) surfaces could decrease with different degrees (17 to 77%), the residual normal and tangential adhesion strength in dry and underwater environment still maintained above 20 kPa, exhibiting excellent energy dissipation capability, surface adaptability, and antipollution properties.

Unlike tunable stiffness (require external field adjustment) (*60*, *61*) and high-stiffness substrates (uncomfortably adapt to undulating surfaces via self-deformation) of adhesives, as well as soft backing (achieve passive intimate contact via discrete or porous structure yet sacrifice adhesion strength) (*43*, *62*), the hierarchical architecture of the proposed all-in-one soft-rigid hybrid robot tracks, now successfully realized, enables macroscopic conformal contact with undulating surfaces via buckling deformation of its discrete pillar array (without external fields) and markedly balances interface stress, suppresses interface crack propagation (*44*, *63*), and boosts detachment thresholds (*61*, *64*) by using rigid core (fig. S10) and discrete configuration. Thereinto, the integration of a rigid core substantially enhances the adhesion performance of the HMSAMS patch, yielding 21 and 9.5% improvements in normal and tangential adhesion strength under dry condition and 39.8 and 36.8% improvements underwater. Meanwhile, the rigid core augments the robot track’s peeling strength by 138% (dry) and 150% (underwater) relative to its coreless counterpart, significantly improving long-time attachment capability. Furthermore, the discrete pillar array structure obviously enhances substrate indentation depth, achieving ~36-fold greater penetration compared to the solid substrate. Thanks to this contact adaptability on uneven surface, the robot track can reliably handle objects of different sizes, surface curvatures, materials, and weights in dry and underwater environments, even with shifted center of gravity [Fig. 3E (i)].

Differing from the electromagnetic WCRs (limited to magnetic surfaces) (*12*, *65*), dry-adhesive ones (restricted to dry surfaces) (*21*, *66*), hydrogel-based one (only on conducive surfaces) (*50*), or negative-pressure ones (requiring nonslippery surfaces) (*14*, *15*), the track-type untethered WCR simultaneously lowers the preference for specific material, topography, humidity, and inclination. By dynamically adjusting preload-governed adhesion strength, the cross-medium WCR overcomes the motion instability caused by the difference in medium density and becomes, to our knowledge, the first reported prototype to demonstrate seamless water-to-air transitions from different water-exit angles or directions while maintaining robust wall-climbing capability across diverse media, materials, and terrains. Compared to the WCRs without HMSAMSs, the pressure of NPAS applied on the proposed cross-medium WCR with HMSAMSs can be directly reduced by 61 to 97% (text 16 and fig. S39). A significant reduction in negative pressure substantially lowers the overall energy consumption of the WCR, especially when compared to its counterparts using negative pressure (*15*, *67*, *68*) or electromagnetic (*69*) adsorption. Although the WCRs based on permanent magnets (*70*, *71*) may have lowest energy consumption (i.e., highest overall efficiency) because no energy is consumed in the adsorption and preload operations, its unadjustable preload will increase extra energy consumption during the movement process, and application scenarios are only limited on magnetic surface. Although the synergistic multimechanism adsorption, such as combining hook-and-claw with negative pressure adsorption (*72*) or integrating negative pressure with dry adhesion (*30*, *73*), enhances surface adaptation and adhesion stability for WCRs on unstructured terrains to some extent, such WCRs are still insufficient to realize cross-medium mobility on various vertical or inverted surfaces. Meanwhile, the proposed cross-medium WCR has a wide operational range relative to tethered alternatives (*74*) and simultaneously exhibits a faster crawling speed than multi-foot or legged WCRs (*50*) and longer zero-power attachment time compared to conventional wheel-type WCRs (*67*). Specifically, the cross-medium WCR with rigid-core–enhanced peeling threshold significantly improves flow-impact resistance underwater, prevents overturning/slipping on vertical and inverted surfaces, and maintains prolonged attachment for long-term low-power monitoring operations [e.g., ground-level trees (Fig. 6A) and floaters and underwater plants (Fig. 6B)]. Moreover, the cross-medium robot is better suited for precise position and posture control in confined multimedia spaces, thereby effectively complementing the disadvantage of cross-medium drones only operating in open environments (*24*).

Actually, the proposed cross-medium WCR is a proof-of-concept prototype, with further optimization potential in both dimensions and functionalities (e.g., ground-wall transition and intelligent adaptive control). Notably, the HMSAMS fabrication uses double exposure-filling technique, as opposed to two-photon polymerization method (*75*), offering distinct advantages in cost-effectiveness and production yield that facilitate scalable manufacturing of such cross-medium WCR.

## MATERIALS AND METHODS

### HMSAMS fabrication

The HMSAMSs were fabricated by the double exposure-filling technique (*33*). More specifically, the photoresist (AZ 4620) was spin coated on glass (coated with 10-nm-thick chrome) at 1000 rpm, followed by soft baking at 95°C for 20 min. Subsequently, the photoresist was exposed to G-line ultraviolet light at a power of 10 mJ cm^−2^ for 35 s in the presence of a mask, and, then, maskless exposure was performed from the bottom side of the glass for 5 s. A hollow mushroom-shaped negative mold was formed by developing the exposed photoresist in sodium hydroxide solution (0.5 wt %) for about 4 min. Next, the as-prepared well-mixed uncured PDMS polymer (mass ratio of base to cross-linker of 10:1) was coated onto the hollow mushroom-shaped negative mold, and its thickness was controlled at 1 mm. After vacuum defoaming for ~10 min and heating at 85°C for 4 hours, an adhesive film with an HMSAMS array was obtained. The morphology and fabrication process of HMSAMSs are illustrated in fig. S40.

### Normal adhesion measurement of HMSAMS patch

For normal adhesion testing in both dry and underwater environments, the HMSAMS patch was first fixed horizontally in a petri dish. Subsequently, the petri dish was fixed on the base of a vertical uniaxial testing machine (PERFECT Instrument Company, Dongguan, China), and the HMSAMS patch was repeatedly pressed by a glass slide (length, 3.3 mm; and width, 3 mm) at a speed of 2 mm/min (fig. S41). The corresponding adhesion force was recorded by the integrated load cell (PT-2KG or PT-1172OZK) of the uniaxial testing machine. Thereinto, in dry condition, the air pressure in cavity of uniaxial testing machine was respectively sustaining 0.002 and 0.101 MPa, and the petri dish was kept dry; in underwater condition, the petri dish was filled with water, and the air pressure in cavity of uniaxial testing machine was kept 0.002 and 0.101 MPa, respectively. Notably, the cavities of HMSAMSs were filled with water in underwater testing.

### Tangential adhesion measurement of HMSAMS patch

For tangential adhesion testing in dry and underwater environments, a glass slide (contact area, 25 mm by 20 mm) was placed on the HMSAMS film and a weight was placed on the glass slide to obtain a given preload. Then, the free ends of the HMSAMS film and glass slide were fixed on the vertical uniaxial testing machine (PERFECT Instrument Company, Dongguan, China) (fig. S41). The corresponding tangential adhesion force was recorded by the integrated load cell (PT-5KG) by vertically moving the sensor upward at 2 mm/min. In particular, the HMSAMS patch was respectively kept dry in dry environment testing, and immersed in water in underwater testing (the cavity of HMSAMSs were filled with water).

### Detachment model used in MD simulations

In the models depicted in Fig. 2H-i, a silicon substrate, with its (100) crystal plane used as the top surface, was placed at the bottom of the simulation box. A PDMS matrix (density, 0.9 g/cm^3^), composed of PDMS molecular chains with a polymerization degree of 20, was layered atop the silicon substrate. In addition, 72,000 water molecules were distributed around the PDMS matrix. The final dimensions of the MD model were 150 Å by 110 Å by 300 Å, with the silicon substrate and PDMS matrix containing 50,850 and 52,416 atoms, respectively. Periodic boundary conditions (PBCs) were applied along the three orthogonal directions of the simulation box. To eliminate PBC-induced artifacts, a vacuum layer with a thickness of 100 Å was introduced above the PDMS matrix. All interactions were modeled using the polymer consistent force field, i.e., an ab initio–derived force field capable of representing a wide range of polymer functional groups. Throughout the simulation, a cutoff distance of 10 Å was adopted and the silicon substrate was fixed. All MD simulations were conducted within the NVT ensemble (i.e., the number of particles N, the volume V, and the temperature T of the system were kept constant) at a temperature of 300 K, which was maintained by a Nosé-Hoover thermostat. Before the tensile simulations, 10-ns relaxation simulations were performed on models with varying configurations to allow water molecular layers of different thicknesses at the PDMS-silicon substrate interface to reach equilibrium. These equilibrated models were then used as the initial configurations for the tensile simulations.

### Fabrication of robot track

The bottom layer structure (i.e., 1.5-mm-thick PDMS film with stop block) and intermediate layer structure (i.e., 0.5-mm-thick PDMS film was patterned with a periodic array of pillars (diameter, 1.5 mm; and height, 2.5 mm) spaced 4 mm apart (center-to-center) were obtained by 3D printed templates. The top layer was discrete core-shell HMSAMS patch units, one of which was integrated by the as-prepared HMSAMS patch (length, 4 cm; width, 5 mm; and thickness, 1 mm), PI tape (length, 4 cm; width, 5 mm; and thickness, 0.01 mm), ceramic plate (length, 4 cm; width, 5 mm; and thickness, 0.5 mm), and 3M tape (length, 4 cm; width, 5 mm; and thickness, 0.5 mm). Subsequently, the robot track was assembled by bonding the top, intermediate, and bottom layer structures together using uncured PDMS. The detailed fabrication process of robot track is illustrated in fig. S42.

### Peeling strength test of the robot track

First, the robot track (length, 16 cm; and width, 4 cm) was horizontally pressed on the glass plate in dry and underwater environments under a given preload (7.8 kPa). Subsequently, the glass plate (length, 20 cm; and width, 10 cm) was horizontally fixed on the base of the vertical uniaxial testing machine, and the free end of the robot track was attached to the load cell via a string. The peeling strength of robot track in dry and underwater environments was recorded by the load cell as it was vertically moving upward at 5 mm/min.

### Contact adaptability simulation of robot track with and without discrete pillars

The discrete pillar array was adopted in the substrate of the proposed robot tracks [Fig. 3D (i)], while a solid PDMS block served as the substrate for the control sample [Fig. 3D (ii)]. Both the robot track and control sample were pressed against a semicylindrical surface (radius, 5 cm) with a preload of 5 N. In the simulation, the density, Young’s modulus, and Poisson’s ratio were set to 2500 kg m^−3^, 68.4 GPa, and 0.25, respectively, for glass; 7500 kg m^−3^, 400 GPa, and 0.23, respectively, for ceramic plate; 1012 kg m^−3^, 0.4 MPa, and 0.49, respectively, for 0.5-mm-thick 3M tape. The Mooney-Rivlin strain energy potential was adopted to describe the hyperelastic behavior of PDMS, setting the *C*_10_, *C*_01_, and *D*_1_ coefficients at 0.243243, 0.060811, and 0.133333, respectively (*76*). Thereinto, in Fig. 3D (iii), the highest contact point between the sample and the semicylindrical surface was designated as the coordinate origin.

### Fabrication of WCR

The robot mainly comprised 3D printed plastic components, metal parts, motors, a NPAS, and electronic control devices. More specifically, the plastic WCR structures were fabricated via 3D printing device (Bambu Lab, X1-Carbon). Both the negative pressure motor (Shenzhen Qunxi Brushless Motor Factory) and electronic speed controllers (Weifang Ninglang Trading Co., Ltd.) were waterproofed by glue (Kafuter, k946), as demonstrated in fig. S43. The 3D printed plastic components were assembled by screws or glue, and the electronic control devices were fixed on the robot via double-sided sponge tape. Figure S20 presents the system diagram of the onboard electronics and the power supply for the WCR, and fig. S18 depicts the physical features of waterproofed cross-medium WCR.

### NPAS assessment

The pressure of the NPAS was measured via an air pressure sensor (HDP500; range, 0 to ~−50 kPa, Huaibei Huadian Automation Technology Co., Ltd.) in the air and underwater. Motor speed was measured by a high-precision photoelectric speedometer [UT372D, Youlide Technology (China) Co., Ltd.] and adjusted by a servo tester. The different pressures of the NPAS were obtained by varying the motor speed via the servo tester.

### Normal and tangential adhesion testing of WCR under different conditions

For dry and underwater adhesion testing, the WCR was pressed on the vertical glass plate (25 cm by 25 cm) under a given preload in dry environment and underwater, respectively. The corresponding normal and tangential adhesion forces were obtained by vertically and horizontally moving the load cells (PT-5KG or PT-50KG) at 1 mm/min. For the cross-medium testing, the WCR was fixed on the vertical glass plate and a given preload was horizontally applied to the robot. In this case, part of the robot was above the water-air interface and the other below. Subsequently, the load cells (PT-5KG or PT-50KG) pulled the WCR horizontally and vertically at 1 mm/min, and the corresponding normal and tangential adhesion forces of the WCR under different water-exit body lengths were quantitatively measured. Notably, the WCR was connected with the load cell via a string in aforementioned experiments.

### Characterization on WCR

The structural morphology of HMSAMSs was characterized via field-emission scanning electron microscopy (SU8010, HITACHI). A digital camera (Nikon, D7500) was used to capture the macroscopic appearance of the WCR and record its wall-crawling videos. HMSAMS size was measured by a laser scanning confocal microscope (Olympus OLS4000). Contact adaptability simulations of robot track were conducted using the ABAQUS software (v. 2020, Dassault Systèmes Simulia Corp., USA). All MD simulations were performed using the large-scale atomic/molecular massively parallel simulator.
