# Supplementary material for: Bioinspired cross-medium wall-climbing robot with high-performance adhesion and contact adaptability
Source: Sci Adv. 2026 Jan 7;12(2):eaea8014. doi: 10.1126/sciadv.aea8014 (PMC12778059; doi:10.1126/sciadv.aea8014)
Supplement: Supplementary file 1 — Supplementary Text S1 to S16 Tables S1 to S6 Figs. S1 to S43 Legends for movies S1 to S32 [file sciadv.aea8014_sm.pdf]

Science Advances
AAAS

Supplementary Materials for

Bioinspired cross-medium wall-climbing robot with high-performance adhesion and contact adaptability

Haoran Liu *et al.*

Corresponding author: Hongmiao Tian, hmtian@xjtu.edu.cn; Qiguang He, qiguanghe@cuhk.edu.hk; Jinyou Shao, jyshao@xjtu.edu.cn

Sci. Adv. **12**, eaea8014 (2026)
DOI: 10.1126/sciadv.aea8014

The PDF file includes:

Supplementary Text S1 to S16
Tables S1 to S6
Figs. S1 to S43
Legends for movies S1 to S32

Other Supplementary Material for this manuscript includes the following:

Movies S1 to S32

Supplementary Text S1: Normal adhesion performance of different architectures

To identify high-performance adhesive structures, four different structural features were engineered to assess their adhesion performance. Fig. S2-A demonstrates schematic diagrams and scanning electron microscopy (SEM) images of the proposed HMSAMSs, mushroom-shaped adhesive microstructures, hollow micropillars, and micropillars. Normal adhesion measurements were conducted by commercial testing equipment in dry and wet environments at 0.101-MPa atmospheric pressure. Figs. S2-B, C, and D show that the adhesive film comprising HMSAMS arrays exhibited a clear-cut increase in its adhesion forces with increasing preload in dry, underwater, and ethanol environments. This phenomenon clearly indicates that microstructural features significantly affect adhesion performance.

In fact, HMSAMSs are hollow mushroom-shaped adhesive microstructures that depend mainly on the negative pressure effect within the hollow cavity and the intermolecular forces between thin cap brims and substrate. To better elucidate the contribution of hollow micropillars and thin cap brims to normal adhesion, the normal adhesion of the proposed HMSAMSs, mushroom-shaped adhesive microstructures, hollow micropillars, and micropillars was measured under dry and underwater conditions. According to Fig. S2-E, the maximum adhesion force (~241.4 kPa) of the HMSAMS patch in dry condition was 1.57 times that of the mushroom-shaped adhesive microstructures (~114.8 kPa) and the hollow micropillars (~38.9 kPa) together. Underwater, the maximum adhesion force of the HMSAMS patch (~289 kPa) was roughly 1.7 times that of the mushroom-shaped micropillars (~149.5 kPa) and the hollow micropillars (~41.9 kPa) together. Based on the two relationships, it can be inferred that HMSAMSs with inner and outside cap brims likely improve adhesion force more effectively than single-brim mushroom-shaped pillars. In absolute ethyl alcohol, the adhesion of the mushroom-shaped adhesive microstructures, hollow micropillars, and micropillars was very small or even zero while that of HMSAMSs remained at ~80 kPa, proving that the coupling function of hollow cavities and double thin cap brims could significantly enhance normal adhesion. Meanwhile, the micropillars exhibited weak adhesion in both dry and wet environments, verifying the significance of thin cap brims and hollow cavities in adhesion enhancement.

Supplementary Text S2: The influence of structural parameters on the adhesion performance of HMSAMS patch

Due to the excellent adhesion performance of HMSAMSs compared to the other microstructures, HMSAMS was chosen as the ideal microstructure to manufacture adhesive films. In the study, the height of HMSAMSs was about 18 μm. Fig. S6-A reveals the preload-dependent normal adhesion of HMSAMS patch under different surface conditions: underwater (0.101 MPa), underwater (0.002 MPa), dry (0.101 MPa), and dry (0.002 MPa). In the four conditions, normal adhesion exceeded 180 kPa and exhibited no sign of attenuation when the preload increased from 120 kPa to 420 kPa, demonstrating stable adhesion under large preload levels. In other word, the selected height of HMSAMSs could prevent undesirable bending deformation under normal preload while preserving its adhesive performance.

Moreover, cap length, an essential structural parameter, also has a strong effect on normal and tangential adhesion forces. In dry condition, normal adhesion increased from 50 kPa to 250 kPa (preload: 80 kPa) when the cap length changed from 3 μm to 12 μm; underwater, it increased from 75 kPa to 290 kPa (preload: 80 kPa) (Fig. S3-A). Similarly, the tangential adhesion of HMSAMS (preload: 80 kPa) patch increased from 78.8 ± 2.25 kPa to 94.6 ± 2.4 kPa in dry environment and from 80.9 ± 2.4 kPa to 96.4 ± 1.28 kPa under water as the cap length increased from 8.45 μm to 12.8 μm (Fig. S3-B). Therefore, increasing cap length is an effective solution to improve normal and tangential adhesion performance. Importantly, the normal and tangential adhesion of the

adhesive film remained high in both dry and underwater conditions, effectively enhancing cross-medium adhesion performance.

Normal adhesion was evaluated across a range of microstructure dimensions (outer diameter, inner diameter, and center-to-center spacing, in μm): 20-8-35, 50-30-80, 70-50-110, 100-80-150, and 130-100-200. In test, the cap length of HMSAMS was 12 μm. According to the comparison results, the 50-30-80 structure achieved higher normal adhesion than the other structures in both dry and underwater conditions (Figs. S3-C and D), which mainly because the large diameter resulted in a significant reduction in the number of HMSAMSs per unit area. Hence, this structure (50-30-80) was adopted in the following studies.

Supplementary Text S3: Normal and tangential grasping ability of HMSAMS patch on different target surfaces

To demonstrate the normal grasping ability of HMSAMS patch in dry and underwater environments, an HMSAMS patch (4 cm × 4 cm) was mounted on a robotic arm (AH5005, Quotient Kinematics Machine) to vertically grasp objects varying in shape, weight, and surface curvature at a set speed (**Figs. S4** and **5**). The tangential grasping ability of the HMSAMS patch under different conditions was also verified by keeping the objects horizontally in air and water for a while (**Figs. S7** and **8**).

Supplementary Text S4: Adhesion performance comparison and analysis of HMSAMS patch under different conditions

To analyze the effect of water molecules on normal adhesion of HMSAMS patch, experiments were conducted in 0.002-MPa vacuum chamber (excluding the effect of atmospheric pressure). **Fig. S9-A** depicts the traction-separation curve of HMSAMS patch under a preload of 100 kPa in both dry and underwater environments. Thereinto, the layer between the polymer and the substrate is ideally considered as a cohesive layer, and the slope of the traction-separation curve (k_1 and k_2) represents its interface stiffness. The slope of the curve in dry condition (k_2) was smaller than that (k_1) underwater. From the microscopic perspective, stiffness is related to the atoms and interatomic bond energy of the material; higher interface stiffness corresponds to greater cohesive layer bonding energy. Therefore, the higher interface stiffness of the cohesive layer underwater than that in air can be mainly attributed to the contribution of water molecules. Meanwhile, the area under the traction-separation curve represents the energy required for HMSAMS patch detaching from the substrate. The fracture energy underwater is significantly higher than that in air, meaning that more energy is required for detachment when water molecules exist between cap brims and substrate.

On this basis, the slopes of the traction-separation curves under different preloads in dry and underwater conditions (atmospheric pressure: 0.002 MPa) were calculated (**Fig. S9-B**). The underwater slope k_1 was larger than the in-air value k_2 when the preload increased from 8 kPa to 100 kPa. The value of k_2 tended to slightly decrease with increasing preload, while k_1 was still increasing. Their difference (k_1-k_2) also increased with increasing preload and the thickness of water layer between cap brims and substrate became thinner. Once the water layer was sufficiently thin, the attraction between water molecules and PDMS as well as the substrate molecules played a dominant role. At this point, the total adhesion force of the adhesive film underwater improved significantly. Meanwhile, under the atmospheric pressure of 0.101 MPa and identical preload, the values of k_1 and k_2 were extremely close, which was mainly ascribed to the existence of atmospheric pressure.

Fig. S9-C compares the fracture energy in the air (atmospheric pressure: 0.101 MPa). The preload-dependent fracture energy in both dry and underwater conditions significantly increased when the

preload increased from 8 kPa to 100 kPa. However, the underwater fracture energy consistently exceeded the dry-condition value under same preload. When the preload exceeded 60 kPa, the fracture energy difference between underwater and dry conditions increased progressively. This enhancement primarily resulted from thinner water molecules strengthening the adhesion force.

Supplementary Text S5: Effect of rigid core on normal adhesion of HMSAMS patch

To study the effect of rigid core on the normal adhesion of HMSAMS patch, a sandwich-like adhesive sample (i.e., core-shell HMSAMS patch) and a control sample were employed. From top to bottom, the proposed structures were a 1-mm-thick HMSAMS patch, 0.5-mm-thick ceramic plate, and 0.5-mm-thick 3M tape, and the control sample was a 1-mm-thick HMSAMS patch, 0.5-mm-thick PDMS film, and 0.5-mm-thick 3M tape (**Fig. S10-A**). The width of the proposed structure and control sample was 5 mm. Under identical preload, the proposed structure exhibited greater stress concentration compared to the control sample during attachment and detachment (**Fig. S10-B**). However, it demonstrated significantly higher adhesion force than the control sample under the same preload (**Fig. S10-C**), clearly illustrating that the rigid core of substrate could evidently enhance the normal adhesion of HMSAMS patch.

Supplementary Text S6: Contact adaptability of robot track on semi-cylindrical surfaces

The excellent contact adaptability of robot tracks endows the cross-medium WCR with enhanced crawling ability on a wide range of terrains. Indentation depth was adopted in this study as an essential indicator to reflect the contact adaptability of robot tracks. Contact adaptability experiments were conducted by pressing the proposed robot track and control sample (solid substrate) onto a semi-cylindrical surface, and then recording and comparing their values of indentation depth. **Fig. S11-A** presents the indentation depth and contact stress distribution of discrete (proposed structure) and solid (control sample) substrates under a preload of 5 N. Although the proposed track structure exhibited higher stress concentration under 5-N preload, its indentation depth was larger than the control sample. Meanwhile, according to the simulation results in **Fig. S11-B**, the indentation depth of the proposed robot track significantly exceeded that of the control sample under identical preload. Therefore, the discrete substrate is an effective architecture to enhance contact adaptability by providing more deformation space.

Supplementary Text S7: Demonstration of normal and tangential grasping ability of robot track

The normal grasping ability of the robot track in dry and underwater conditions was demonstrated by fixing the robot track on a robot arm (AH5005, Quotient Kinematics Machine). After vertically attaching various target objects of different materials under a given preload in dry (**Fig. S14**) and underwater (**Fig. S15**) environments, the robot track was sustained in the air for a while to verify its stable grasping ability. In addition, its tangential grasping ability was further demonstrated by horizontally maintaining target objects of different materials in dry (**Fig. S16**) and underwater (**Fig. S17**) environments for certain seconds. **Figs. S12** and **13** respectively demonstrate normal and tangential grasping ability of robot track on target objects of different shapes (yet identical material) in underwater and dry environments.

Supplementary Text S8: Implementation of the cross-medium WCR

The main plastic components of the wall-climbing robot (WCR) were manufactured via 3D printing (Bambu Lab, X1-Carbon), and two DC slow-speed waterproof motors (dimensions: 29 mm × 14.5 mm ×12.5 mm; driving voltage: 1.5-9 V) were used as the driving motors. To control the preload on the robot tracks in water and air, a 5000-kV brushless motor was integrated in the negative pressure absorption system (NPAS). The WCR control system mainly comprised brushless electronic speed controller (ESC), brushed ESC, receiver, remote control, and batteries (**Fig. S19**). **Fig. S20** shows the transmission process of the WCR control signal (green arrow) and power (red arrow). A radio frequency of 900 MHz was chosen to support the communication needs of the WCR. The brushless and brushed ESCs received PWM signals by the receiver; and the speeds of the driving motors and negative pressure motor was adjusted by PWM signals. Moreover, the power was supplied by 14.8-V (700 mA·h) and 7.4-V (350 mA·h) batteries for the brushless and brushed ESCs, respectively. The negative pressure motor, batteries, brushless ESC, brushed ESC, and receiver were waterproofed by glue (Kafuter, k946) (**Fig. S43**).

Supplementary Text S9: WCR water-entry ability on acrylic plate at different inclination angles

WCR water-entry experiments were also performed to study its adaptability to different inclination angles (**Fig. S23**). In the experiments, the inclination angles of an acrylic plate were set as 40°, 60°, and 90°, respectively. In this experiment, the WCR could stably dive into water on both positive and negative acrylic plates.

Supplementary Text S10: Simplified force analysis of wall-climbing in different cases

In the force analysis, the tangential direction is parallel to the plate length, while the normal direction is perpendicular to it. For simplicity, NPAS pressure is uniformly applied on the robot tracks. In single medium, the WCR can smoothly crawl on a plate surface, i.e., the crawling speed is constant. However, during cross-medium transitions, the robot struggles to maintain a consistent crawling speed on the surface. In **Fig. S24**, m, v, F_d, F_b, F_f, N, g, $F_{ad\perp}$, and F_r correspond to the mass, speed, driving force, buoyancy force, fluid resistance, support force, gravitational acceleration, normal adhesion force, and rolling resistance of the WCR.

Case 1: The WCR crawls from water into air on a positive surface at a given inclination angle (θ).

Fig. S24-A shows the detailed force analysis of the WCR on a positive surface during the water-exit process. In the tangential direction, the kinetic equation can be written as follows:

$$m\frac{dv}{dt} = F_d + F_b sin\theta - mgsin\theta - F_f - F_r \tag{S1}$$

In the normal direction, the equation is:

$$N + F_b cos\theta = mgcos\theta + F_{ad\perp} \tag{S2}$$

Case 2: The WCR crawls from water into air on a negative surface at a given inclination angle (θ).

The simplified force analysis for the WCR is shown in **Fig. S24-B**. In the tangential direction, the kinetic equation can be written as follows:

$$m\frac{dv}{dt} = F_d + F_b sin\theta - mgsin\theta - F_f - F_r \tag{S3}$$

In the normal direction, it is:

$$F_{ad\perp} + F_b cos\theta = mgcos\theta + N \tag{S4}$$

Case 3: The WCR crawls from air into water on a positive surface at a given inclination angle (θ).

In this case, the simplified force analysis for the WCR is shown in **Fig. S24-C**. In the tangential direction, the kinetic equation can be written as follows:

$$m\frac{dv}{dt} = F_d + mgsin\theta - F_b sin\theta - F_f - F_r \tag{S5}$$

In the normal direction, the equation is:

$$N + F_b cos\theta = mgcos\theta + F_{ad\perp} \tag{S6}$$

Case 4: The WCR crawls from air into water on a negative surface at a given inclination angle (θ).

Fig. S24-D shows the simplified force analysis for the WCR. In the tangential direction, the kinetic equation can be written as follows:

$$m\frac{dv}{dt} = F_d + mgsin\theta - F_b sin\theta - F_f - F_r \tag{S7}$$

In the normal direction, the equation is:

$$F_{ad\perp} + F_b cos\theta = mgcos\theta + N \tag{S8}$$

Case 5: The WCR crawls on a negative surface in the air.

In this case (**Fig. S24-E**), the inclination angle of the plate is 0° and the driving force of the WCR is equal to the rolling resistance of the robot tracks:

$$F_d = F_r \tag{S9}$$

The weight of the WCR is balanced by the normal adhesion force of robot tracks, which is defined as follows:

$$F_{ad\perp} = mg \tag{S10}$$

Case 6: The WCR crawls on a negative surface underwater ($\theta = 0°$).

Underwater (**Fig. S24-F**), the driving force of the WCR in the tangential direction is balanced by the fluid resistance and rolling resistance:

$$F_d = F_r + F_f \tag{S11}$$

In the normal direction, WCR buoyancy force, normal adhesion, and weight are in equilibrium:

$$F_{ad\perp} + F_b = mg \tag{S12}$$

Case 7: The WCR crawls horizontally on a vertical wall in the air ($\theta = 0°$).

In the air (**Fig. S24-G**), forces are applied to the robot in three directions. In the vertical direction (parallel to the wall surface), the vertical adhesion force is equal to weight:

$$F_{ad\parallel} = mg \tag{S13}$$

In the horizontal direction (parallel to the wall surface), the driving force is balanced by rolling resistance:

$$F_r = F_d \tag{S14}$$

Perpendicular to the vertical wall, the normal adhesion and support force are in balance:

$$F_{ad\perp} = N \tag{S15}$$

Case 8: The WCR crawls on a vertical wall underwater ($\theta = 0°$).

Underwater (**Fig. S24-H**), the buoyancy force and fluid resistance are also participated in the force analysis. In the vertical direction (parallel to the wall surface), the vertical adhesion force and buoyancy force are equal to weight:

$$F_{ad\parallel} + F_b = mg \tag{S16}$$

In the horizontal direction (parallel to the wall surface), the driving force is balanced by horizontal adhesion and fluid resistance:

$$F_r + F_f = F_d \tag{S17}$$

Perpendicular to the vertical wall, the support force is equal to normal adhesion:

$$F_{ad\perp} = N \tag{S18}$$

Supplementary Text S11: Anti-slipping conditions of a WCR in a stationary attachment on an inclined negative surface (inclination angle: *θ*)

When the WCR attaches to an inclined negative surface (inclination angle: *θ*), slipping is a common failure mode. Therefore, its static force analysis is conducted in **Fig. S25**. The normal adhesion force of each core-shell HMSAMS patch unit on the robot track in contact with the surface is assumed to be equal. Here, $F_{whole\ friction}$ represents the friction of one robot track, m is the mass of the WCR, $F_{normal\ adhesion}$ is the normal adhesion of a core-shell HMSAMS patch unit, N_1 is the support force of top core-shell HMSAMS patch unit, H is the distance between the center of gravity of the robot and the substrate surface, n is the number of core-shell HMSAMS patch units on the robot track that are attached to the substrate surface, and L is the contact length of the robot track on the substrate surface. When the WCR is static on a negative surface, the friction needs to be larger than the component of gravity:

$$F_{whole\ friction} \geq \frac{G}{2} sin\theta \tag{S19}$$

The friction of a single robot track can also be calculated as:

$$F_{whole\ friction} = \sum_{i=1}^{n} N_i \cdot \mu \tag{S20}$$

where N_i represents the support force of the i-th core-shell HMSAMS patch unit and μ is the friction coefficient. The torque due to the weight of the robot can be calculated as:

$$\sum_{i=1}^{n} F_i l_i = \frac{Gsin\theta \cdot H}{2} \tag{S21}$$

where l_i is the distance between the center of the i-th core-shell HMSAMS patch unit and that of the WCR along the direction parallel to the plate length, and F_i is the torque force applied on the i-th core-shell HMSAMS patch unit.

Hence, the support force of the i-th core-shell HMSAMS patch unit can be calculated by:

$$N_i = F_{normal\ adhesion} - \frac{Gcos\theta}{2n} \pm F_i \tag{S22}$$

Due to the symmetry of the robot structure, the torque magnitudes of the core-shell HMSAMS patch units above and below the center of gravity of the robot are equal but act in opposite directions. At the same time, it is ideally assumed that the normal adhesion of all core-shell HMSAMS patch units is same, and that the gravitational component force of each core-shell adhesive film unit is also identical. Thus, the aforementioned equation can be further simplified:

$$\sum_{i=1}^{n} N_i = n\left(F_{normal\ adhesion} - \frac{Gcos\theta}{2n}\right) \tag{S23}$$

In this case, the friction of a single robot track can be written as:

$$F_{whole\ friction} = \mu \cdot n\left(F_{normal\ adhesion} - \frac{Gcos\theta}{2n}\right) \tag{S24}$$

To prevent the WCR from slipping, the normal adhesion force required for a single core-shell HMSAMS patch unit is:

$$F_{normal\ adhesion} \geq \frac{Gcos\theta}{2n} + \frac{Gsin\theta}{2n\mu} \quad \text{(S25)}$$

Supplementary Text S12: Anti-overturning conditions of a WCR in a stationary attachment on an inclined negative surface (inclination angle: *θ*)

If the load (or weight) of the WCR attached to the underside of an inclined plate is sufficiently large, the robot may pivot around the lowest contact point (*C*) i.e., overturning (Fig. S25). To prevent overturning, the core-shell HMSAMS patch unit at the top of the robot track must remain attached to the substrate surface.

Ideally, the normal adhesion of each core-shell HMSAMS patch unit on the robot track in contact with the substrate surface is identical. Since the robot track does not transmit torque, the normal adhesion force of the top core-shell HMSAMS patch unit provides the anti-overturning torque.

In the normal direction, the normal adhesion and support force of a single robot track are in balance:

$$n \cdot F_{normal\ adhesion} = \sum_{i=1}^{n} N_i \quad \text{(S26)}$$

The equation for the torque at point *C* can be written as follows:

$$\sum M_c = M_1 + M_2 + M_3 = 0 \quad \text{(S27)}$$

where M_1 is the torque of a core-shell adhesive patch unit at the top of the robot track in contact with the substrate surface, M_2 is the torque of *mg* in the normal direction, and M_3 is the torque of *mg* in the tangential direction (*m* is the mass of WCR, and *g* is gravitational acceleration).

The equations for M_1, M_2, and M_3 are presented as follows:

$$M_1 = (F_{normal\ adhesion} - N_1) \cdot L \quad \text{(S28)}$$

$$M_2 = \frac{Gl_c cos\theta}{2} \quad \text{(S29)}$$

$$M_3 = \frac{GHsin\theta}{2} \quad \text{(S30)}$$

where l_c is the distance between point *C* and the center of gravity of the WCR along the direction parallel to the plate length.

When the support force of the core-shell HMSAMSs unit at the top of robot track in contact with the substrate surface is greater than or equal to zero, the core-shell adhesive film unit will remain attached to the substrate surface. At this point, the normal adhesion provided by a single core-shell HMSAMS patch unit can be calculated by:

$$F_{normal\ adhesion} \geq \frac{G(l_c cos\theta + Hsin\theta)}{2L} \quad \text{(S31)}$$

Supplementary Text S13: Application demonstration of HMSAMS patch under different conditions

To demonstrate the potential applications of HMSAMS patch, multiple demonstrations in both dry and wet environments were conducted. As depicted in **Fig. S29-A**, by fixing an HMSAMS patch on the back of a toy drone, the toy drone could attach to dry and wet glass surfaces at high speed, which provided an effective and straightforward solution for using the drone to monitor targets for long periods of time and eliminated its dependence on its on-board batteries. Moreover, harnessing

the adhesive function of the HMSAMS patch in dry and wet conditions, miniature surveillance equipment could also attach to moving objects for motion tracking or data collection. Examples include a parasitic device securely attached to a flying toy drone for aerial surveillance, to a ground-moving toy for topographical information collection, or to a toy boat for underwater data acquisition (**Fig. S29-B**).

Furthermore, a HMSAMS patch (10 mm × 30 mm), attaching even to hairy skin surfaces, could easily lift a 2-kg weight, clearly demonstrating its excellent tangential adhesion performance on wet and uneven surfaces (**Fig. S29-C**). Moreover, the HMSAMS patch (20 mm × 50 mm) did not exhibit rapid crack propagation on the back of a hand (**Fig. S29-D**) under unilateral external force, verifying its exceptional adhesive performance.

Supplementary Text S14: Stress distribution and deformation of a single HMSAMS during attachment/detachment in different environments

Cap brim deformation of a single HMSAMS during the attachment and detachment processes was directly observed via a finite element analysis software (ABAQUS 2020). The fracture energy, and separation stiffness were adjusted to simulate the actual deformation of the HMSAMS in different conditions (dry and underwater). To this end, the bilinear cohesive zone model was employed to describe the fracture behavior of the HMSAMS. **Table S5** shows the separation stiffness, damage initiation stress, and fracture energy for different conditions. The Mooney-Rivlin strain energy potential was adopted to describe the hyperelastic behavior of PDMS, with the C_{10}, C_{01}, and D_1 coefficients being 0.243243, 0.060811, and 0.133333, respectively (**Table S6**).

In dry condition (atmospheric pressure: 0.101 MPa), the hollow cavity was in a positive-pressure state when pressing on the substrate. Subsequently, during detachment, the inner cap brim of the HMSAMS detached first from the substrate due to the presence of negative pressure in the hollow cavity (**Fig. S30-A**). Thereinto, the preload was 22.6 kPa, and the adhesion force was about 81 kPa.

Underwater (atmospheric pressure: 0.101 MPa), the hollow cavity was in a positive-pressure state due to the compressive deformation of the HMSAMS during attachment. The inner cap brim exhibited clear upturned deformation during detachment due to pressure difference (**Fig. S31-A**). Thereinto, the preload was 26.376 kPa, and the adhesion force was about 101.1 kPa.

In all conditions, stress concentration in the HMSAMS mainly occurred at the cap brim-hollow micropillar joint during attachment and detachment.

Supplementary Text S15: Simulation of HMSAMS

The suction cup force of a HMSAMS mainly originates from the pressure difference between the external atmospheric pressure and the internal pressure within the suction cavity, which can be expressed as:

$$\Delta p = p_b - p_a \tag{S32}$$

where p_a denotes the pressure of cavity before deformation (i.e., atmospheric pressure, approximately 101.3 kPa) and p_b represents the internal pressure of the suction cavity of HMSAMS after deformation. Since the p_a can generally be considered constant before the deformation of HMSAMS, the key task is to analyze the internal pressure changes (i.e., p_b) during deformation.

To simplify the analysis, the fluid inside the suction cavity is assumed to be an ideal fluid, satisfying the law:

$$p_b V_b = N_b R_b T \tag{S33}$$

where V_b represents the volume of fluid inside the suction cavity, N_b represents the number of fluid molecules, R_b is the ideal fluid constant, and T is the temperature of the fluid. Since the temperature variation during attachment and detachment is relatively small, T can be regarded as constant. This equation indicates that as the cavity volume V_b increases, the internal pressure p_b decreases accordingly.

When the cavity of HMSAMS deforms, the relationship between the internal fluid volume and its geometric parameters can be expressed as:

$$V_b = V_a(\frac{r_b}{r_a})^2 \frac{H_b}{H_a} \quad \text{(S34)}$$

where V_a is the volume of cavity before deformation, r_a and r_b are the radius of cavity before and after deformation, H_a and H_b are the height of cavity before and after deformation. This equation illustrates that the volume change of the cavity is relied on the variation of radius and height of cavity. Here, we ideally think that the radius variation is relatively small during deformation.

Based on the above equation (S34), we can determine the size of the cavity volume at different stages. when the preload is vertically applied on the top of HMSAMS (**Fig. S32-i**), the V_b decreases due to the decrease of height. At this time, p_b and Δp increase (i.e., $\Delta p > 0$). However, during the vertical lifting process of the HMSAMS (stage II, **Fig. S32-ii**), the cavity pressure difference (i.e., Δp) transitions from positive to negative (i.e., $\Delta p < 0$) as H_b increases with deformation. At stage III (**Fig. S32-iii**), the pressure difference (i.e., Δp) is also need to applied on the outer cap brim.

Based on the above qualitative analysis, we provide the pressure differences (Δp) within the cavity under different preload conditions in simulation. Underwater (atmospheric pressure: 0.101 kPa), we set the preload to 6.78 kPa, 26.376 kPa, 45.1 kPa, 68.45 kPa, and 89.17 kPa respectively, and corresponding pressure difference inside the cavity of HMSAMS is 10 kPa, 20 kPa, 30 kPa, 40 kPa, and 50 kPa. Meanwhile, underwater (atmospheric pressure: 0 MPa), pressure difference inside the cavity of HMSAMS under different preloads is set to 0 kPa.

Besides underwater simulation, the simulation under dry condition is also conducted. In dry environment (atmospheric pressure: 0.101 MPa), the preload is set to 3.33 kPa, 22.6 kPa, 42 kPa, 66.57 kPa, and 86.35 kPa, and corresponding pressure difference inside the cavity of HMSAMS is 5 kPa, 15 kPa, 25 kPa, 35 kPa, and 45 kPa. Meanwhile, in dry environment (atmospheric pressure: 0 MPa), pressure difference inside the cavity of HMSAMS under different preloads is set to 0 kPa.

The cohesive zone model is used to describe the adhesive behavior between the cap brims of the HMSAMS and substrate. Interface stiffness, maximum nominal fracture stress and fracture energy of a single HMSAMS in simulation can be seen in **Table S5**.

As shown in **Fig. S33-A** (underwater, 0 MPa), the normal adhesion in experiment increased with the preload because larger preload was conducive to realize intimate contact between HMSAMS and the substrate. However, the normal adhesion in simulation nearly sustained ~85 kPa under different preloads because the contact between HMSAMS and the substrate was ideal. In **Fig. S33-B** (underwater, 0.101 MPa), the normal adhesion in both experiment and simulation increased with the preload. By comparing the simulation results in Figs. S33-A and B, we believe that the negative pressure inside the cavity can increase the normal adhesion of HMSAMS underwater.

In **Fig. S33-C** (dry, 0 MPa), the normal adhesion increased with preload in experiment, and nearly kept same in simulation. However, the normal adhesion in **Fig. S33-D** (dry, 0.101 MPa) increased with the preload in experiment and simulation. From Figs. S33-C and D, preload-triggered negative pressure can directly increase normal adhesion of HMSAMS.

In experiments, the cavity of HMSAMS can generate sufficient negative pressure to lower the spacing between the cap brims and substrate and realize their intimate contact, thereby enhancing

the intermolecular forces. This enhanced intermolecular forces effectively seal the cavity of the HMSAMS to prevent air leakage. Hence, intermolecular forces and negative pressure act in concert to enhance the adhesion strength. In the simulations, the observed improvement in normal adhesion arises from the superposition of negative pressure and intermolecular forces, rather than from a synergistic enhancement between the two. This might be the reason for the differences between experiments and simulations.

Supplementary Text S16: The evaluation of pressure and energy consumptions for WCR

For the cross-medium WCR based on preload-dependent HMSAMS patches, its total energy consumption mainly comes from the following parts:

(1) Mobility energy consumption. This is the main energy consumption for robot to overcome gravity and movement resistance.

(2) Adsorption energy consumption. For the HMSAMS patches, the energy consumption is theoretically zero. This is one of the core advantages of this method.

(3) Energy consumption of the auxiliary system. This energy is mainly consumed by the control units, sensors, communication modules, etc.

(4) The energy consumption of preload. Although the HMSAMS patch does not directly consume energy, maintaining preload needs to consume energy.

In order to evaluate the contribution of HMSAMSs to the wall-climbing robot, the pressure provided by NPAS is denoted as P_1, and the area of NPAS and robot tracks contacting with substrate is respectively denoted as S_1 and S_2. Thus, the preload of robot track provided by NPAS is P_1S_1/S_2. In the study, the values of S_1 and S_2 are 37.75 cm^2 and 56 cm^2, respectively. Hence, the preload applied on WCR is $0.67P_1$.

Given that the pressure provided by NPAS is relatively small, the linear equations based on the measured adhesion forces under small preload are fitted, which can be seen in **Fig. S39**.

Case 1: WCR crawls on negative surface in dry environment.

The normal adhesion of WCR provided by HMSAMS patches in dry environment is $23.87P_1$ +79.65 kPa (Fig. S39). If the pressure that meets the WCR to climb on negative surface in dry environment is P, the pressure provided by the NPAS is (P-79.65)/23.87 kPa. If the WCR does not have HMSAMSs on robot tracks, the pressure provided by the NPAS is 1.49P (i.e., $0.67P_1=P$). Thus, total reduction of pressure in NPAS is 1.45P+3.33 kPa, and the corresponding percentage of decrease is >97%.

Case 2: WCR crawls on negative surface under water.

The normal adhesion of WCR provided by HMSAMS patches under water is $14.71P_1$ +7.78 kPa (Fig. S39). If the pressure that meets the WCR to climb on negative surface in dry environment is P, the pressure provided by the NPAS is (P-7.78)/14.71 kPa. If the WCR does not have HMSAMSs on robot tracks, the pressure provided by the NPAS is 1.49P (i.e., $0.67P_1=P$). Thus, total reduction of pressure in NPAS is 1.42P+0.53 kPa, and the corresponding percentage of decrease is >95%.

Case 3: WCR crawls on vertical surface in dry environment.

The tangential adhesion of WCR provided by HMSAMS patches in dry environment is $1.72P_1$ +70.97 kPa (Fig. S39). If the pressure that meets the WCR to climb on vertical surface is P, the pressure provided by the NPAS is (P-70.97)/1.72 kPa. If the WCR does not have HMSAMSs on robot tracks, the pressure provided by the NPAS is 1.49P (i.e., $0.67P_1=P$). Thus, total reduction of pressure in NPAS is 0.91P+41.26 kPa, and the corresponding percentage of decrease is >61%.

Case 4: WCR crawls on vertical surface under water.

The tangential adhesion of WCR provided by HMSAMS patches under water is $5.82P_1$ +1.68 kPa (Fig. S39). If the pressure that meets the WCR to climb on vertical surface is P, the pressure provided by the NPAS is (P-1.68)/5.82 kPa. Thus, total reduction of pressure in NPAS is 1.32P+0.29 kPa, and the corresponding percentage of decrease is >89%.

Under the assumption that the negative pressure system dominates the energy consumption in traditional pneumatic wall-climbing robots, a rough comparison can be made between the energy consumption of our proposed robot and that of traditional ones. Based on the aforementioned discussion, the decrease of energy consumption for preload is >97% for case 1, >95% for case 2, >61% for case 3, and >89% for case 4. Meanwhile, the adjustable preload can also adjust the adhesion strength of HMSAMS patches during the crawling process of WCR, which can also be conducive to lower the crawling resistance and decrease overall energy consumption.

In addition, the proposed WCR can attach to vertical and inverted surfaces without consuming any power.

Table S1. Maximum normal and tangential adhesion forces of microstructures based on PDMS in dry and underwater conditions ("-" represents no results or no data presented in the papers)

Features	Normal (dry)	Normal (underwater)	Tangential (dry)	Tangential (underwater)
**OIAs** (25)	~25 kPa	~42 kPa	-	-
**DIAs** (38)	~60 kPa	~55 kPa	-	-
**Biomimetic disc** (24)	~15 kPa	~52 kPa	~24 kPa	~34 kPa
**Stiffness- gradient adhesive structure** (30)	~220 kPa	-	-	-
**Core-shell dry adhesives** (31)	~112 kPa	-	-	-
**Nano-sucker** (32)	~30 kPa	~26 kPa (wet)	~13 kPa	~12 kPa (wet)
**dry adhesives** (33)	~185 kPa	-	-	-
**d-SCC** (34)	~40 kPa	~60 kPa (wet)	~100 kPa	~150 kPa (wet)
**μ-SCs** (29)	30	120	-	-
**EOMS** (35)	85	63	-	-
**Hierarchical architectures** (36)	67	50	-	-
**Adhesive microstructures** (37)	87.8 kPa	~ 43 kPa	~55 kPa	
**SAS** (39)	40 kPa	-	-	-
**HMSAMSs**	**240 kPa**	**290 kPa**	**108.13 kPa**	**127.3 kPa**

Table S2. Maximum ratio of adhesion force and preload of microstructures based on PDMS in normal and tangential direction in dry and underwater conditions ("-" represents no results or no data presented in the papers)

Features	Normal (dry)	Normal (underwater)	Tangential (dry)	Tangential (underwater)
**OIAs** (25)	~1:1	~1:1	-	-
**DIAs** 38)	~3.5:1	~3.5:1	-	-
**Biomimetic disc** (24)	~15:1	~20:1	-	-
**Stiffness-gradient adhesive structure** (30)	~5:1	-	-	-
**Core-shell dry adhesives** (31)	~1:1	-	-	-
**Nano-sucker** (32)	-	-	-	-
**dry adhesives** (33)	-	-	-	-
**d-SCC** (34)	~8:1	~6:1 (wet)	-	-
**µ-SCs** (29)	-	-	-	-
**EOMS** (35)	~7.5:1	~5.5:1	-	-
**Hierarchical architectures** (36)	-	-	-	-
**Adhesive microstructure** (37)	~47.8:1	-	-	-
**SAS** (39)	-	-	-	-
**HMSAMSs**	**792:1**	**57:1**	**182:1**	**12.9:1**

Table S3. Maximum normal and tangential adhesion forces of adhesive films based on different materials in dry and underwater conditions ("-" represents no results or no data presented in the papers)

Features	Material	Preload	Normal (dry)	Normal (underwater)	Tangential (dry)	Tangential (underwater)	Time
**PSA (51)**	SMP	300 kPa	1500 kPa	600 kPa	-	-	>0 s
**Drum (52)**	SMP	75 kPa	-	1800 kPa	-	-	>0 s
**15°CM (57)**	PU	300 kPa	1000 kPa	1300 kPa	-	-	0 s
**Cup (58)**	PU	44 kPa	160 kPa	1000 kPa	-	-	0 s
**Poly (49)**	Hydrogel	-	-	60 kPa	-	-	10 s
**TBVA (50)**	Hydrogel	-	12 kPa	-	-	-	5 s
**TRGA (53)**	Copolymer	8.5 kPa	-	8.1 kPa	-	-	30 s
**Adhesive (54)**	Copolymer	-	-	3 kPa	-	-	>0 s
**Adhesive (55)**	Copolymer	38 kPa	200 kPa	150 kPa	-	-	0 s
**Adhesive (56)**	Coacervate	22 kPa	230 kPa	100 kPa	-	-	60 s
**HMSAMSs**	**PDMS**	**100 kPa**	**240 kPa**	**290 kPa**	**108.13 kPa**	**127.3 kPa**	**0 s**

Table S4. Maximum ratio of adhesion force and preload of adhesive films based on different materials in dry and underwater conditions ("-" represents no results or no data presented in the papers)

Features	Material	Normal (dry)	Normal (underwater)	Tangential (dry)	Tangential (underwater)	Time
**PSA (51)**	SMP	10:1	3:1	-	-	>0 s
**Drum (52)**	SMP	-	68:1	-	-	>0 s
**15°CM (57)**	PU	2:1	4:1	-	-	0 s
**Cup (58)**	PU	4:1	23:1	-	-	0 s
**Poly (49)**	Hydrogel	-	-	-	-	10 s
**TBVA (50)**	Hydrogel	-	-	-	-	5 s
**TRGA (53)**	Copolymer	-	1:1	-	-	30 s
**Adhesive (54)**	Copolymer	-	-	-	-	>0 s
**Adhesive (55)**	Copolymer	5.2:1	3.9:1	-	-	0 s
**Adhesive (56)**	Coacervate	10.5:1	4.5:1	-	-	60 s
**HMSAMSs**	**PDMS**	**792:1**	**57:1**	**182:1**	**12.9:1**	**0 s**

Table S5. Interface stiffness, maximum nominal fracture stress and fracture energy of a single HMSAMS in simulation.

Description	Interface stiffness (N/m)	Maximum nominal fracture stress (N)	Fracture energy (mJ)
**HMSAMS in air (0 MPa)**	5000	0.05 (normal), 0.1 (shear)	5e-6
**HMSAMS in air (0. 101MPa)**	5000	0.05 (normal), 0.1 (shear)	5e-6
**HMSAMS underwater (0 MPa)**	6000	0.06 (normal), 0.12 (shear)	1e-5
**HMSAMS underwater (0.101 MPa)**	6000	0.06 (normal), 0.12 (shear)	1e-5

Table S6. Parameters of Mooney–Rivlin strain energy potential to describe hyperelastic behavior of PDMS. All the data is calculated in millimeter.

Parameter	Description	Value
C_{10}	Material constant	0.243243
C_{01}	Material constant	0.060811
d	Material incompressible parameter	0.133333

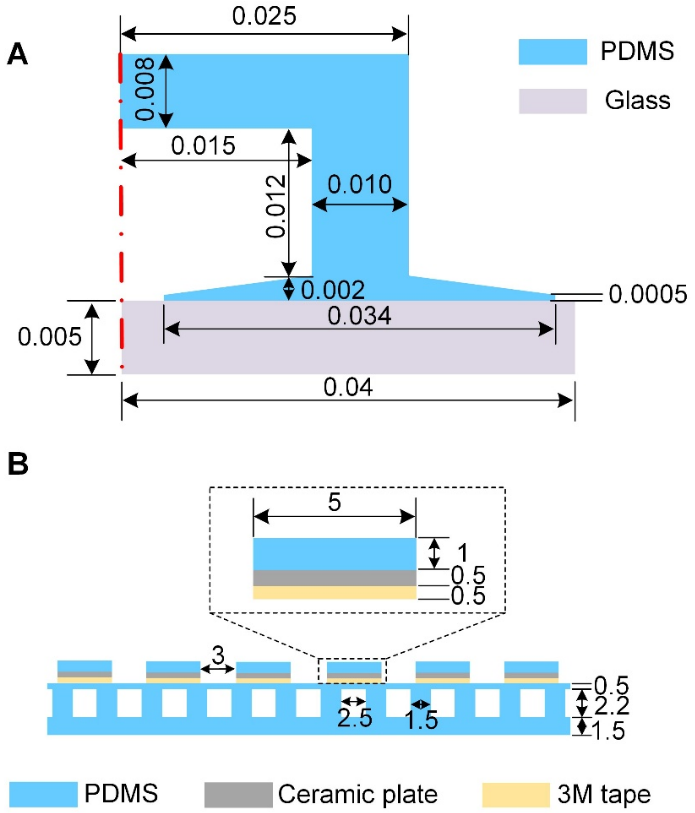

Fig. S1. Geometric parameters of HMSAMS (A) and robot track (B). All dimensions are in mm.

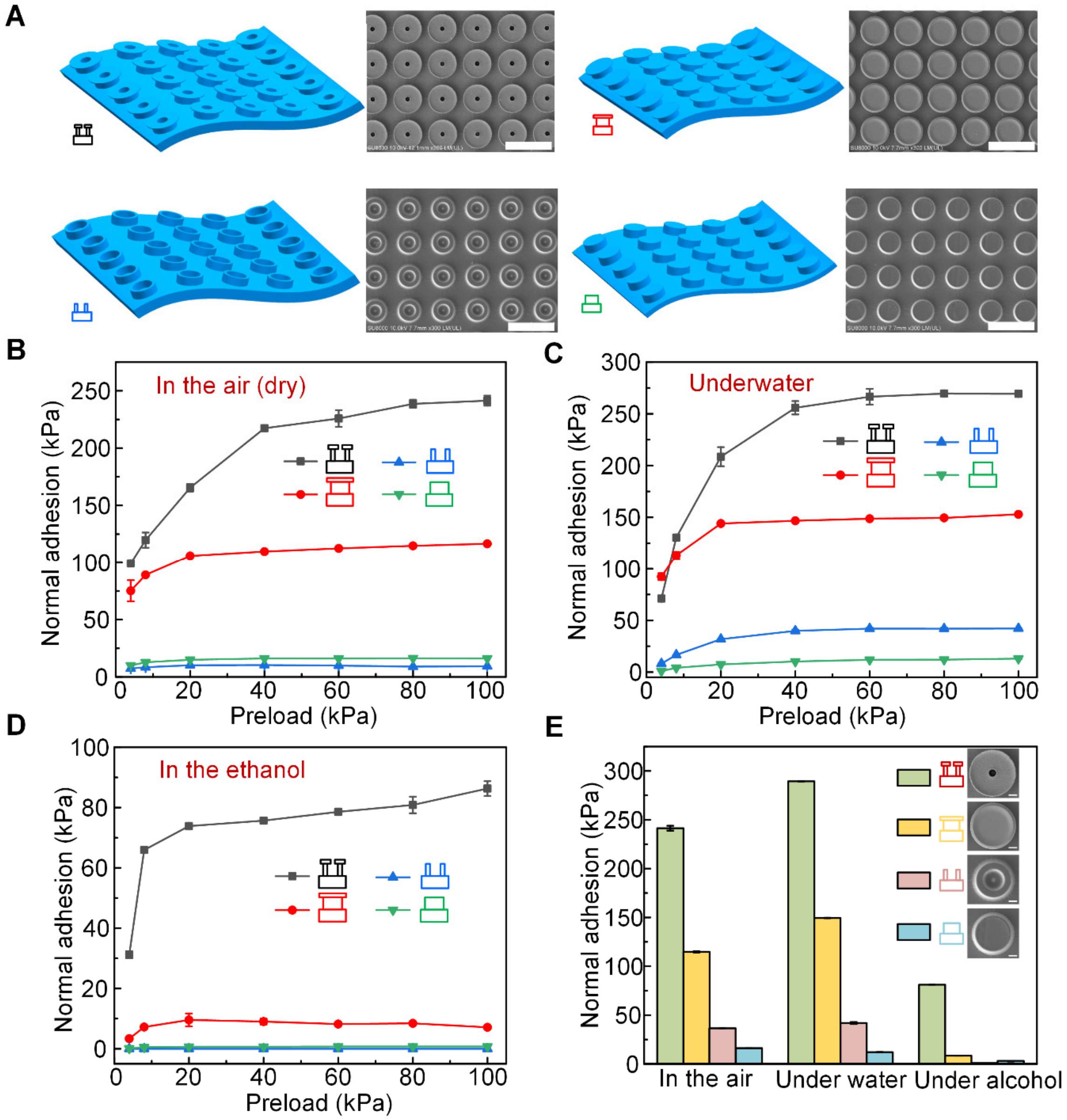

Fig. S2. Normal adhesion measurements of different architectures (the proposed HMSAMSs, mushroom-shaped adhesive microstructures, hollow micropillars, and micropillars) under different conditions (dry, water, and alcohol). (**A**) Diagrams and scanning electron microscope images of four different adhesive architectures: HMSAMSs, mushroom-shaped adhesive microstructures, hollow micropillars, and micropillars. Scale bars: 100 μm. **(B-D)** Preload-dependent normal adhesion curves of the four different adhesive architectures under different surface conditions: dry (B), water (C), and alcohol (D). (**E**) Normal adhesion of four different microstructures (HMSAMSs, mushroom-shaped adhesive microstructures, hollow micropillars, and micropillars) under a preload of 80 kPa in dry, water, and alcohol environments. Scale bars: 10 μm.

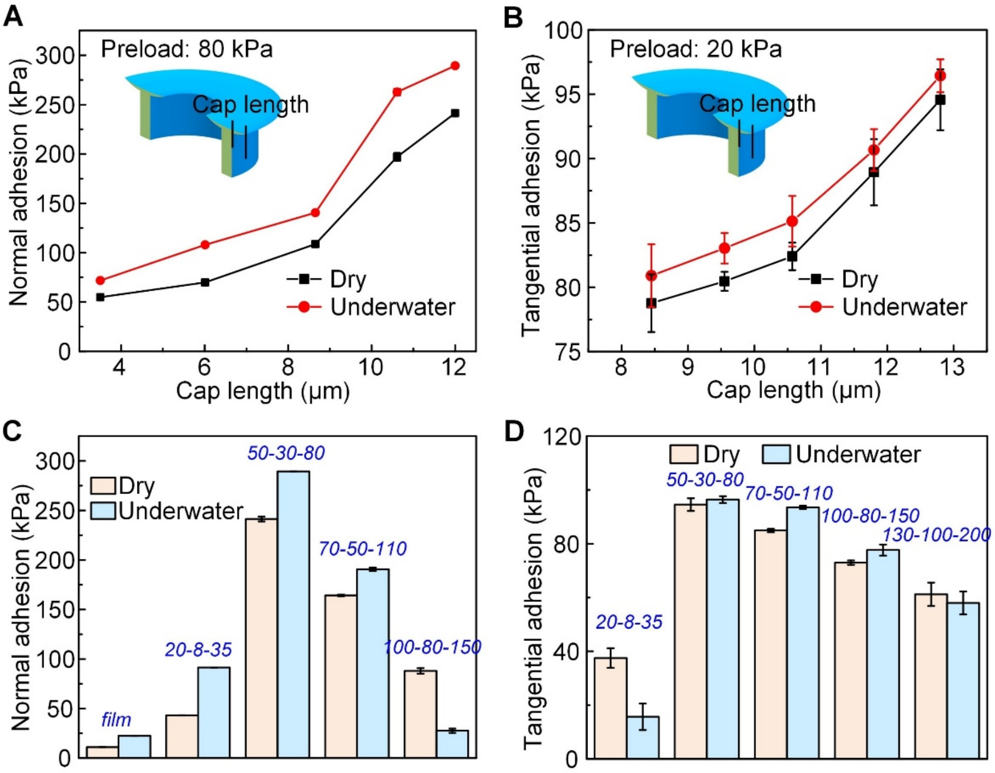

Fig. S3. Influence of structural parameters of HMSAMSs on adhesion performance of HMSAMS patch. (**A**) Influence of cap length of HMSAMSs on normal adhesion of HMSAMS patch in dry and underwater environments. (**B**) Influence of cap length of HMSAMSs on tangential adhesion of HMSAMS patch in dry and underwater environments. (**C**) Normal adhesion of HMSAMS patch with different microstructural dimensions under identical preload (80 kPa) in dry and underwater environments. (**D**) Tangential adhesion of HMSAMS patch with different microstructural dimensions under identical preload (20 kPa) in dry and underwater environments.

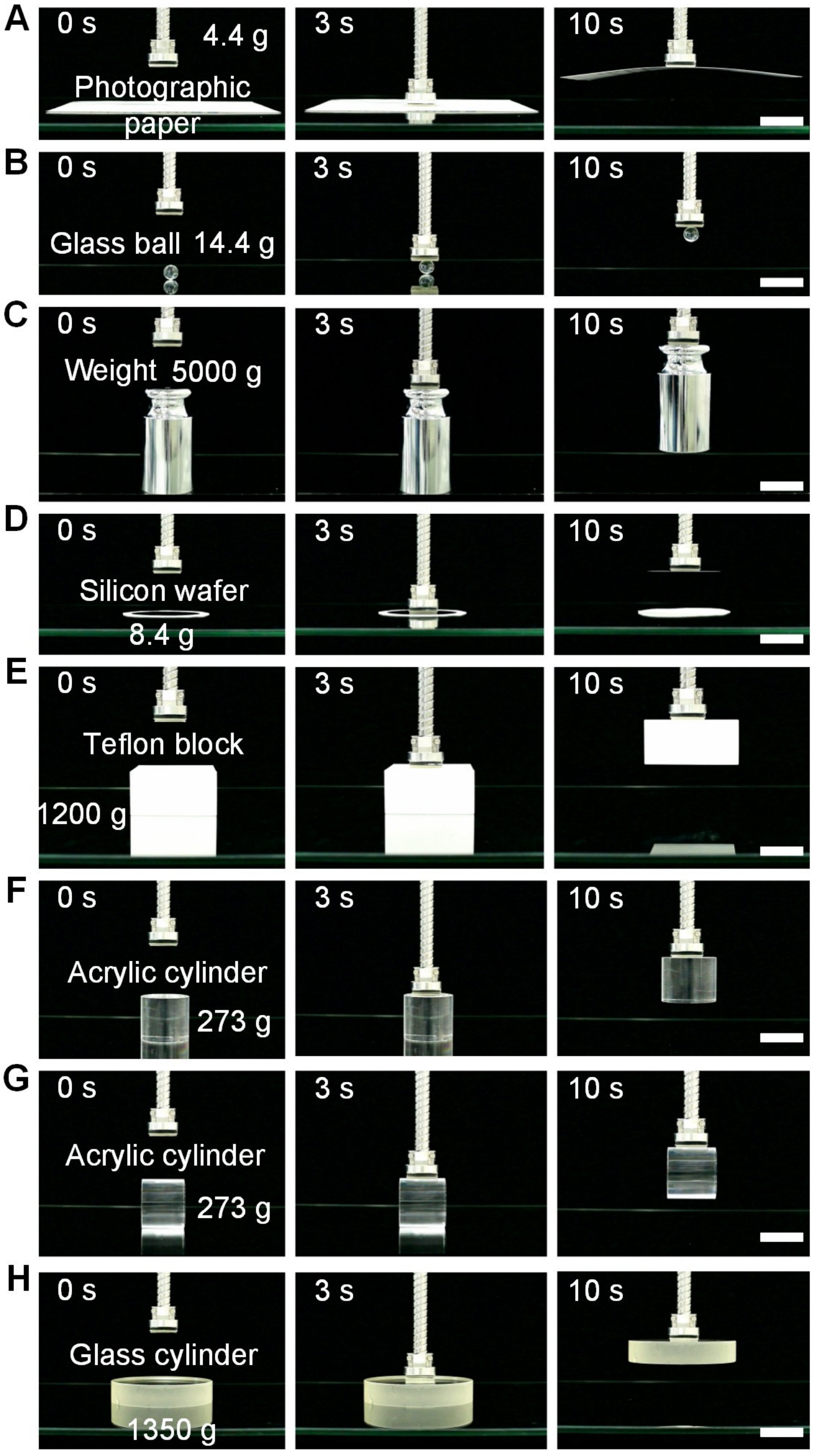

Fig. S4. HMSAMS patch vertically grasping numerous objects under dry condition. The adopted objects, in testing, were various: Photographic paper, 4.4 g (**A**); Glass ball, 14.4 g (**B**); Weight, 5000 g (**C**), Silicon, 8.4 g (**D**); Teflon block, 1200 g (**E**); Acrylic cylinder, 273 g (**F**); Acrylic cylinder, 273 g (**G**); and glass cylinder, 1350 g (**H**). Scale bars: 6 cm.

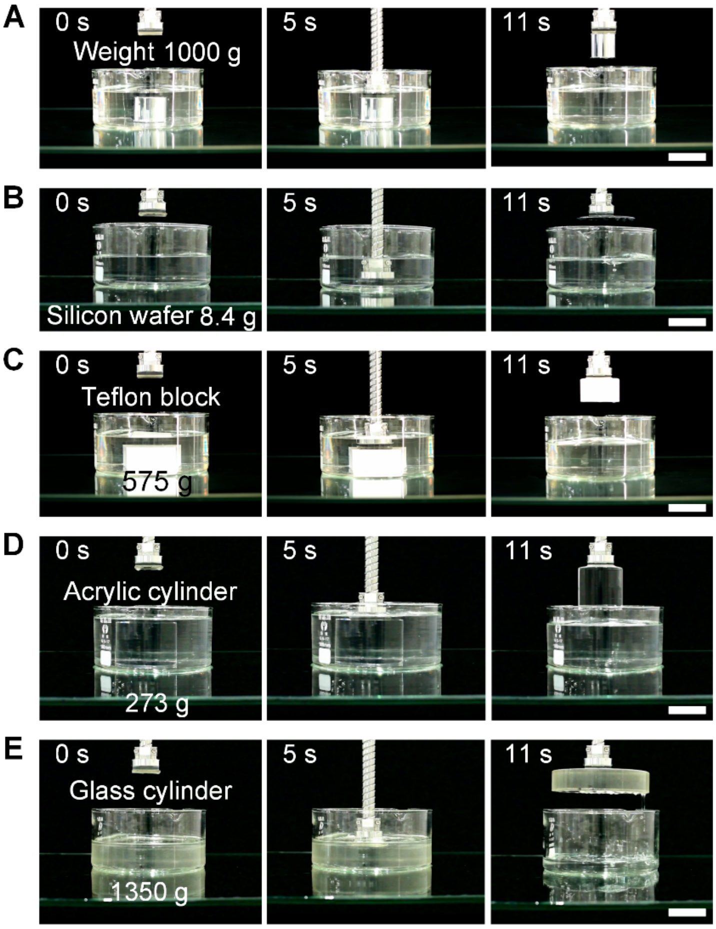

Fig. S5. HMSAMS patch vertically grasping numerous objects underwater. The adopted objects were 1000-g weight (**A**), 8.4-g silicon wafer (**B**), 1200-g Teflon block (**C**), 273-g acrylic cylinder (**D**) and 1350-g glass cylinder (**E**). Scale bars: 6 cm.

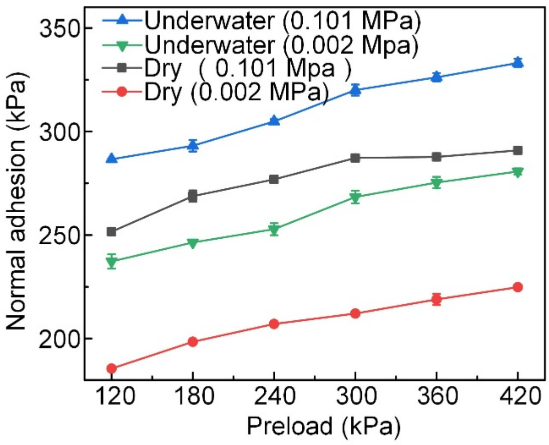

Fig. S6. Preload-dependent normal adhesion curves of HMSAMS patch under different surface conditions: underwater (0.101 MPa), underwater (0.002 MPa), dry (0.101 MPa), and dry (0.002 MPa).

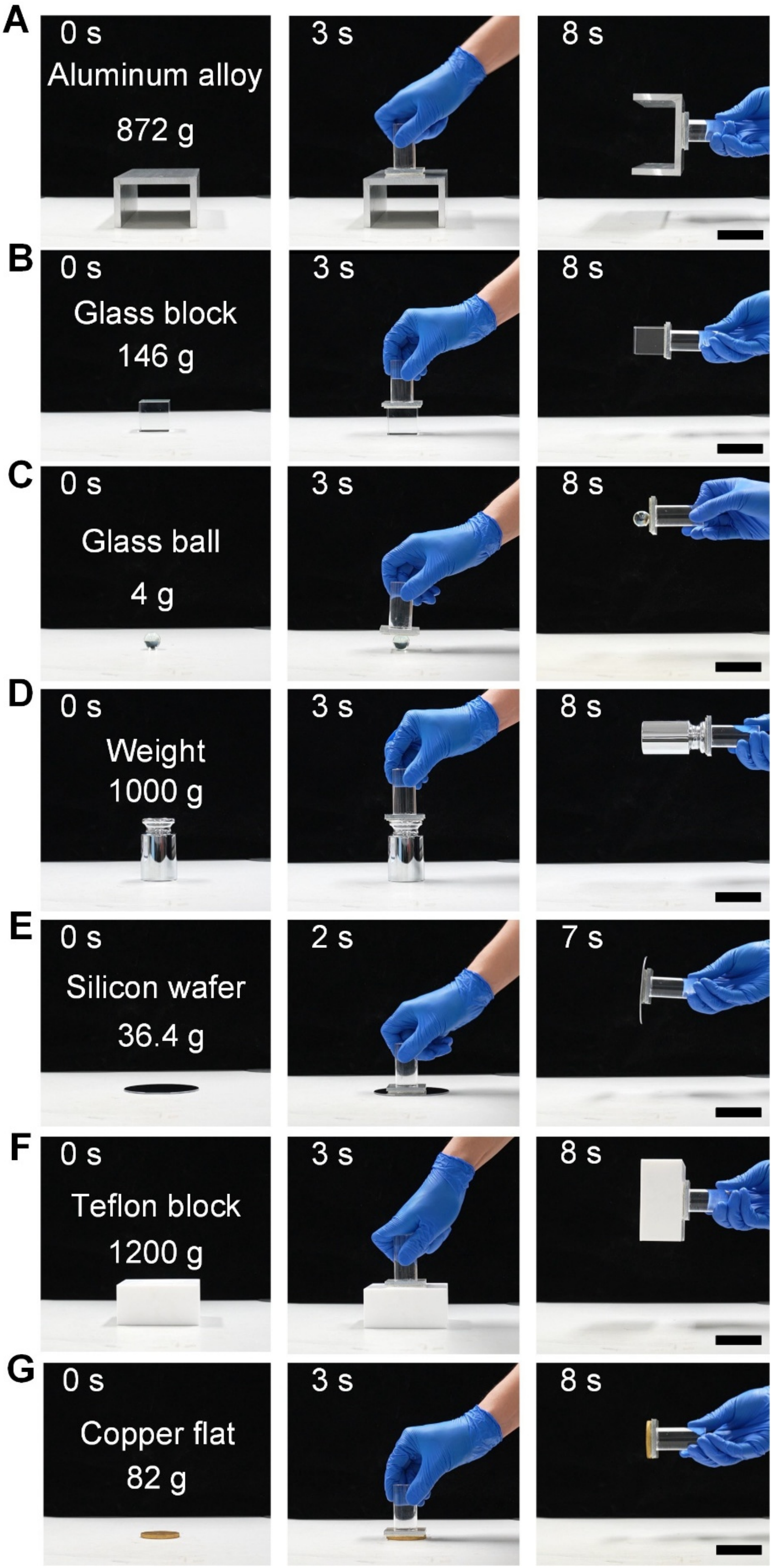

Fig. S7. Tangential adhesion performance demonstration of HMSAMS patch (4 cm × 4 cm) under dry condition. In testing, different objects were attached by HMSAMS patch: Aluminum alloy, 872 g (**A**); Glass block, 146 g (**B**); Glass ball, 4 g (**C**); Weight, 1000 g (**D**); Silicon wafer, 36.4 g (**E**); Teflon, 1200 g (**F**); Copper flat, 82 g (**G**). Scale bars: 6 cm.

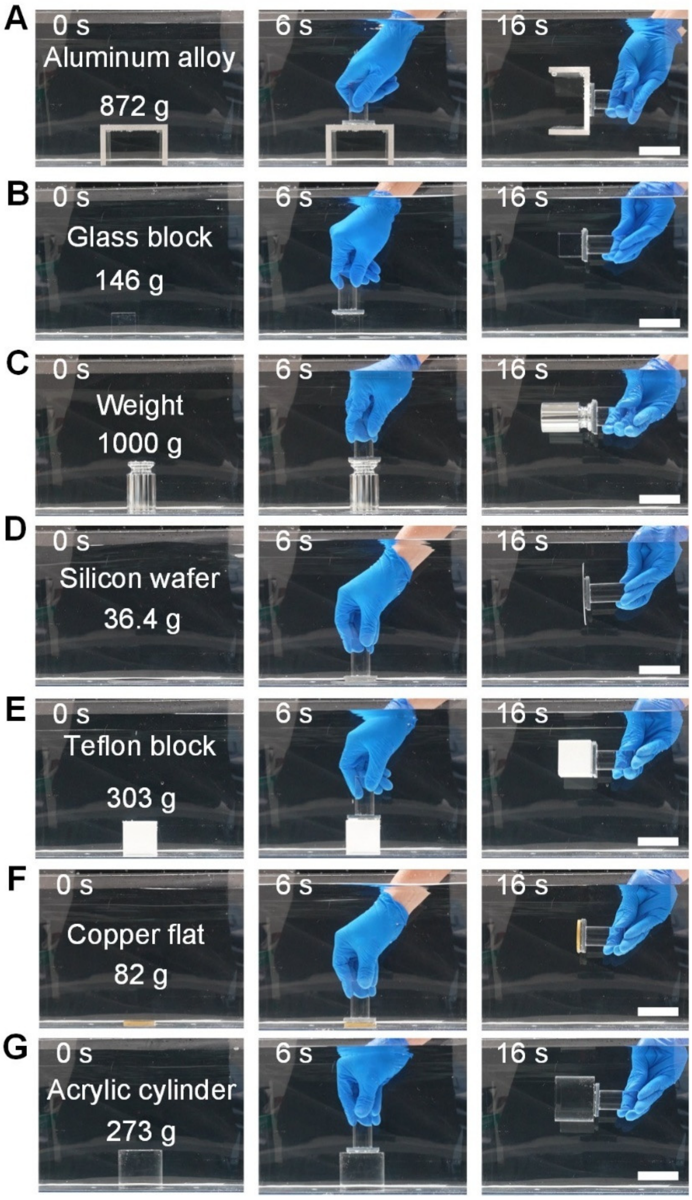

Fig. S8. Tangential adhesion performance demonstration of HMSAMS patch (4 cm × 4 cm) underwater. A photographic time series of HMSAMS patch grasping various objects with different sizes, materials and weights: Aluminum alloy, 872 g (**A**); Glass block, 146 g (**B**); Weight, 1000 g (**C**); Silicon wafer, 36.4 g (**D**); Teflon block, 303 g (**E**); Copper flat, 82 g (**F**); and acrylic cylinder 273 g (**G**). Scale bars: 6 cm.

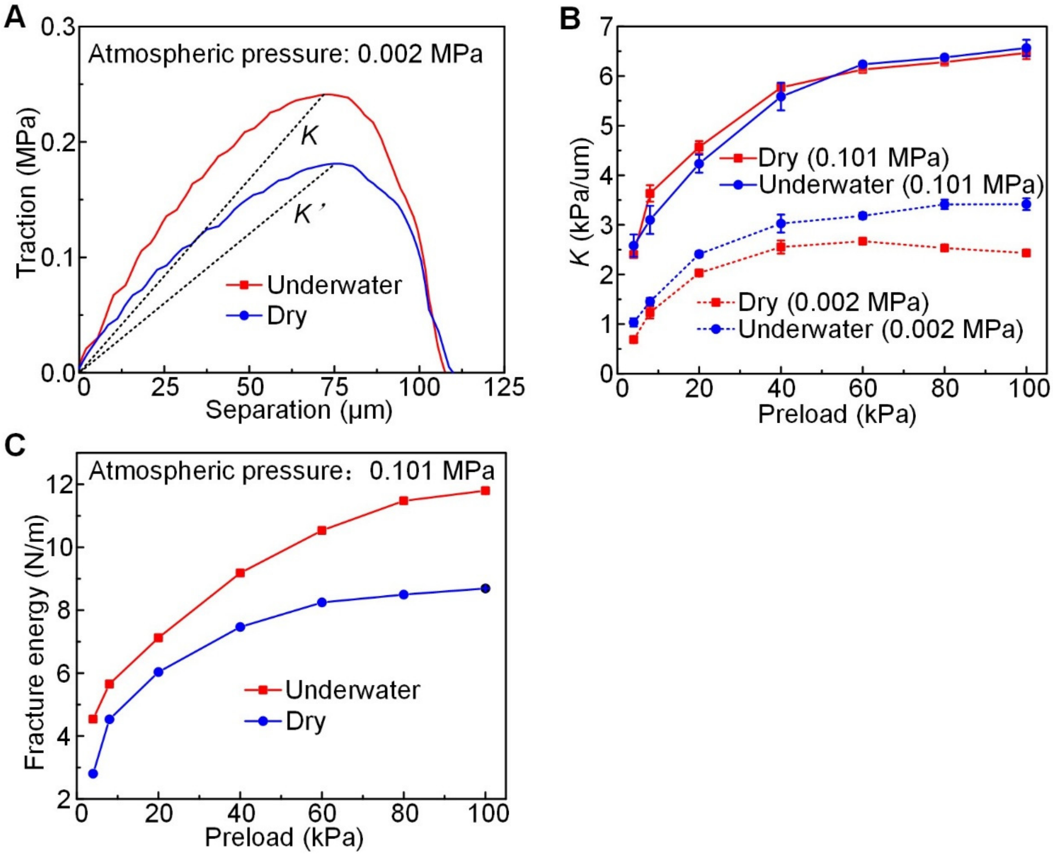

Fig. S9. Comparison of interface stiffness and fracture energy of HMSAMS patch during detachment under dry and underwater conditions. (**A**) Traction-separation curves of HMSAMS patch under a preload of 100 kPa in dry and underwater environments (atmospheric pressure: 0.002 MPa). (**B**) Preload-dependent interface stiffness (*K*) curves of HMSAMS patch in dry and underwater environments under different atmospheric pressures (0.002 MPa and 0.101 MPa). (**C**) Comparison of fracture energy of HMSAMS patch in dry and underwater environments under the normal atmospheric pressure of 0.101 MPa.

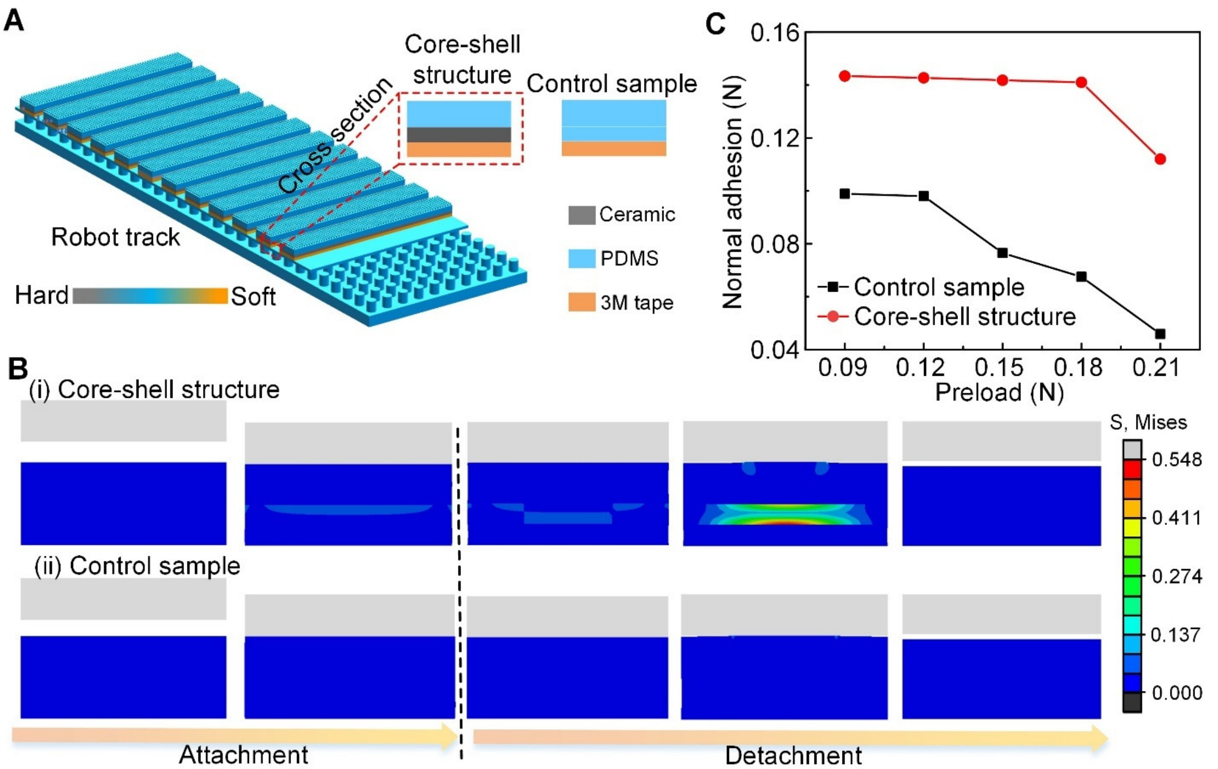

Fig. S10. Influence of rigid core on normal adhesion. (**A**) Schematic diagram of core-shell structure and control sample. (**B**) Stress distribution of core-shell structure and control sample during attachment and detachment processes. (**C**) Preload-dependent normal adhesion curves of core-shell structure and control sample.

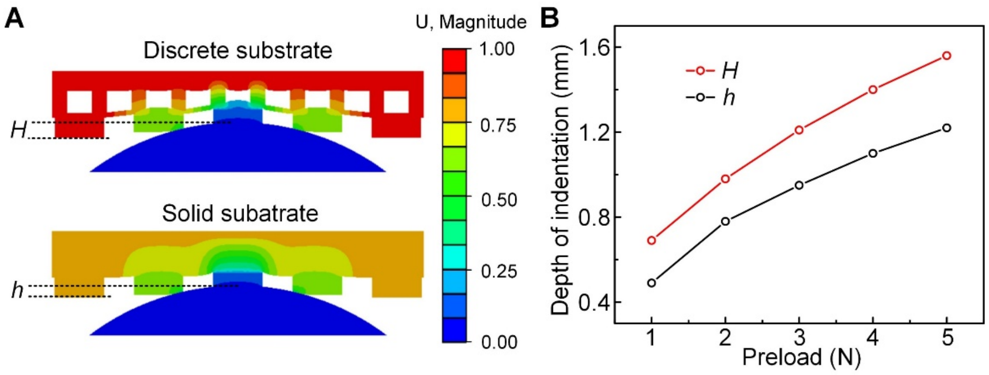

Fig. S11. Contact adaptability of the discrete and solid substrates. (**A**) Indentation depth of the discrete and solid substrates under identical preload (5 N). (**B**) Indentation depth of the discrete and solid substrates under different preloads.

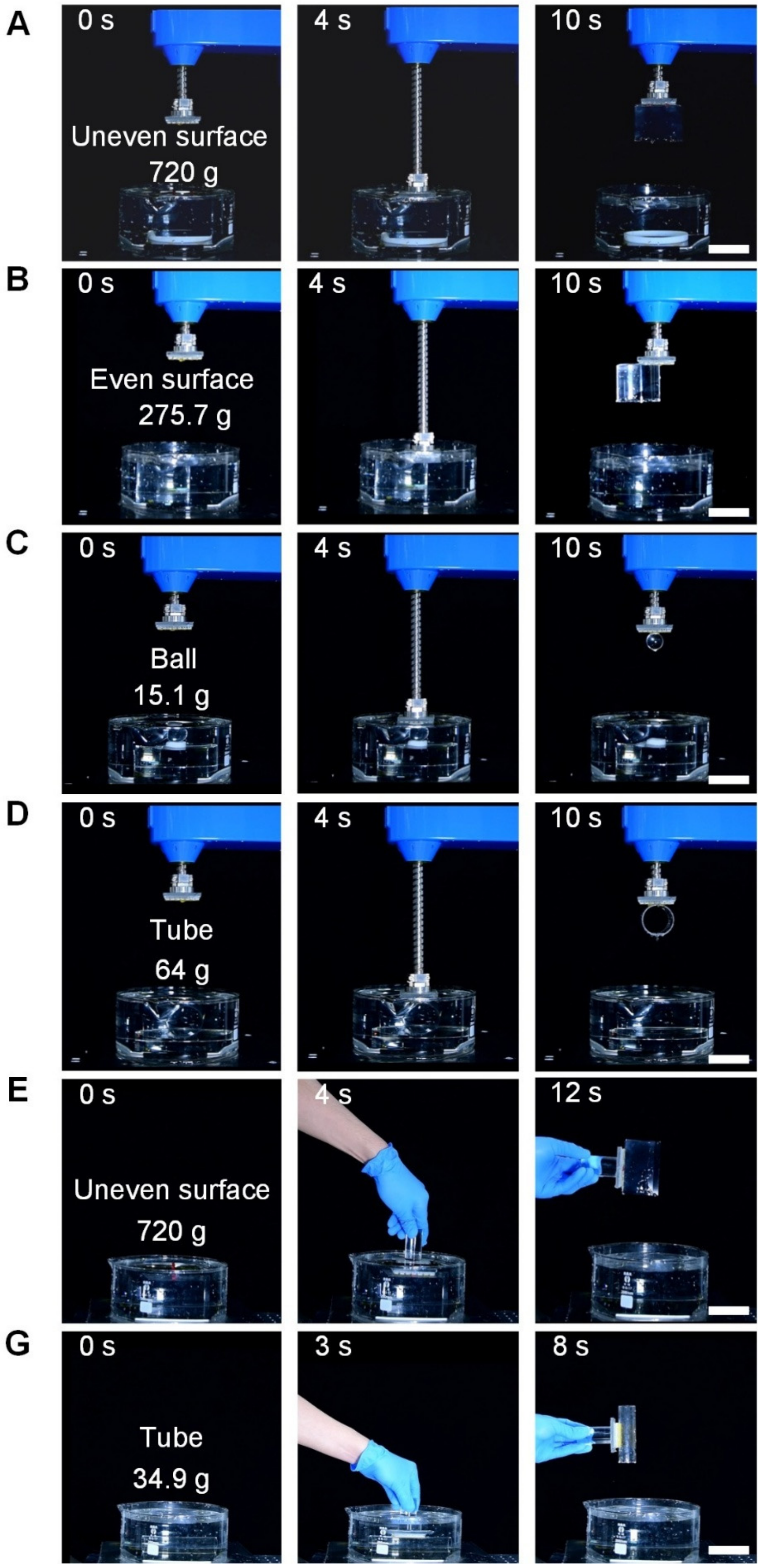

Fig. S12. Normal and tangential adhesion performance demonstration of the proposed robot track by grasping different underwater acrylic objects with various shapes and weights: Uneven surface, 720 g (A); Even surface, 275.7 g (B); Ball, 15.1 g (C); Tube, 64 g (D); Uneven surface, 720 g (E); and tube, 34.9 g (G). Scale bars: 6 cm.

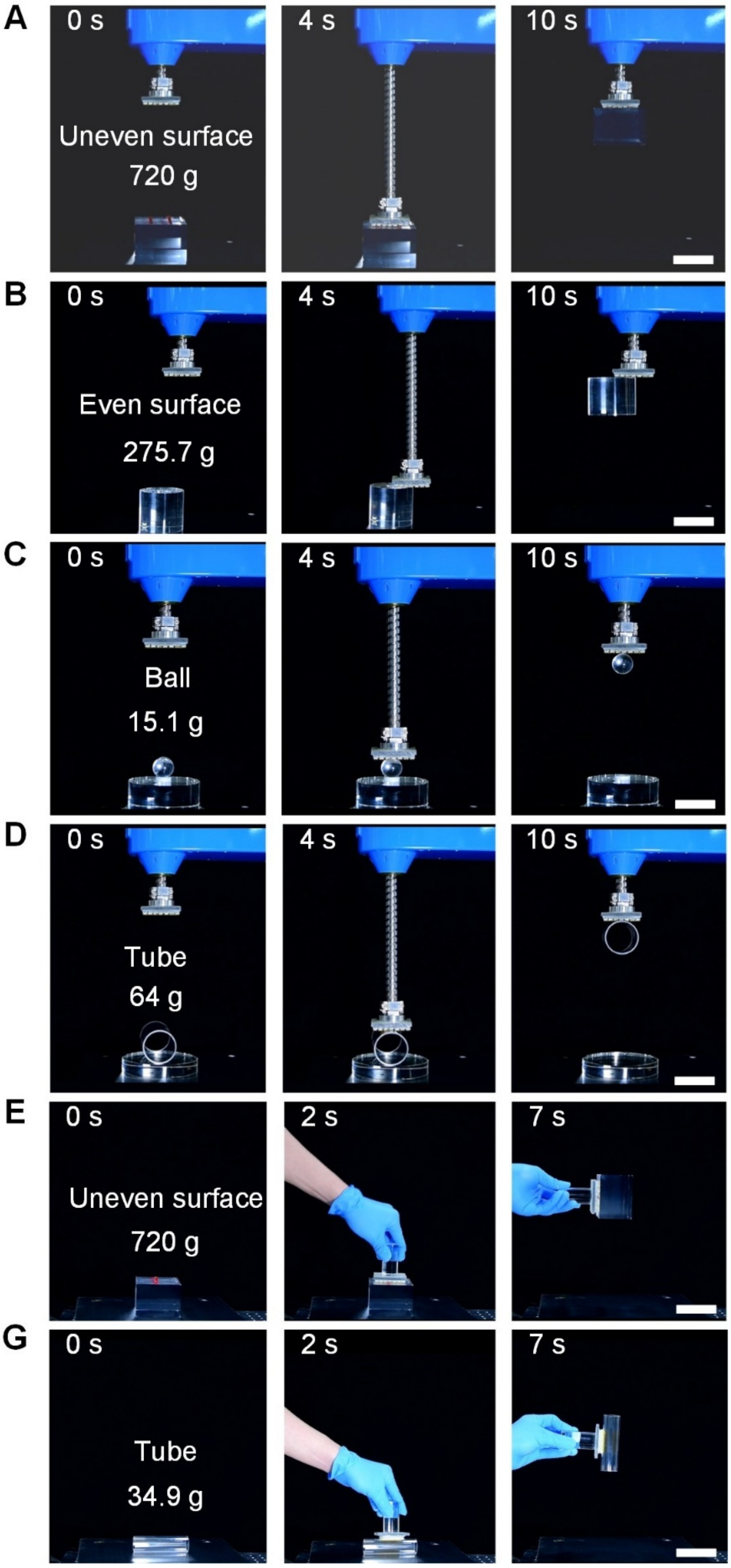

Fig. S13. Normal and tangential adhesion performance demonstration of the proposed robot track by grasping different acrylic objects with various shapes and weights under dry condition: Uneven surface, 720 g (A); Even surface, 275.7 g (B); Ball, 15.1 g (C); Tube, 64 g (D); Uneven surface, 720 g (E); and tube, 34.9 g (G). Scale Bars: 6 cm.

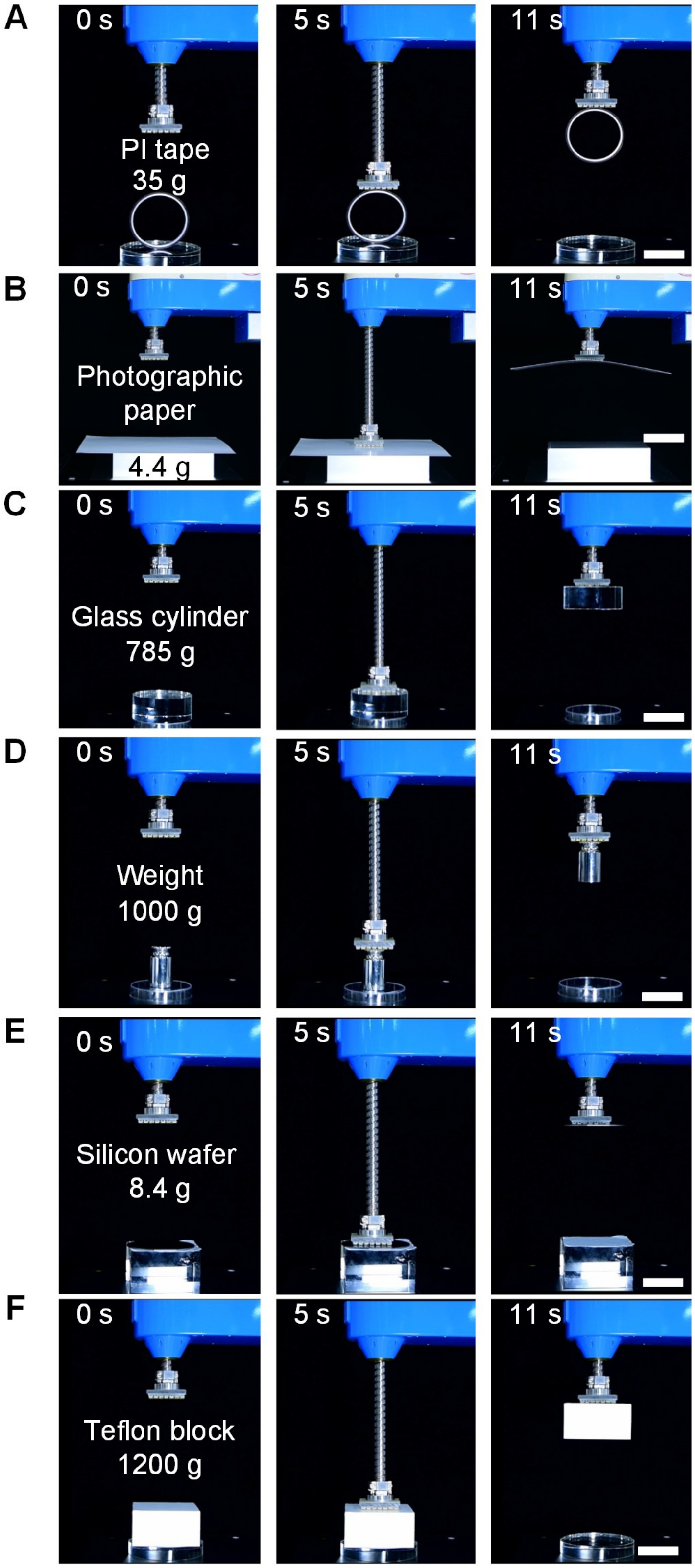

Fig. S14. A photographic time series of robot track vertically grasping different objects with different materials, weights and shapes in dry environment: 35-g PI tape (A), 4.4-g photographic paper (B), 785-g glass cylinder (C), 1000-g weight (D), 8.4-g silicon wafer (E) and 2000-g Teflon block (F). Scale bars: 6 cm.

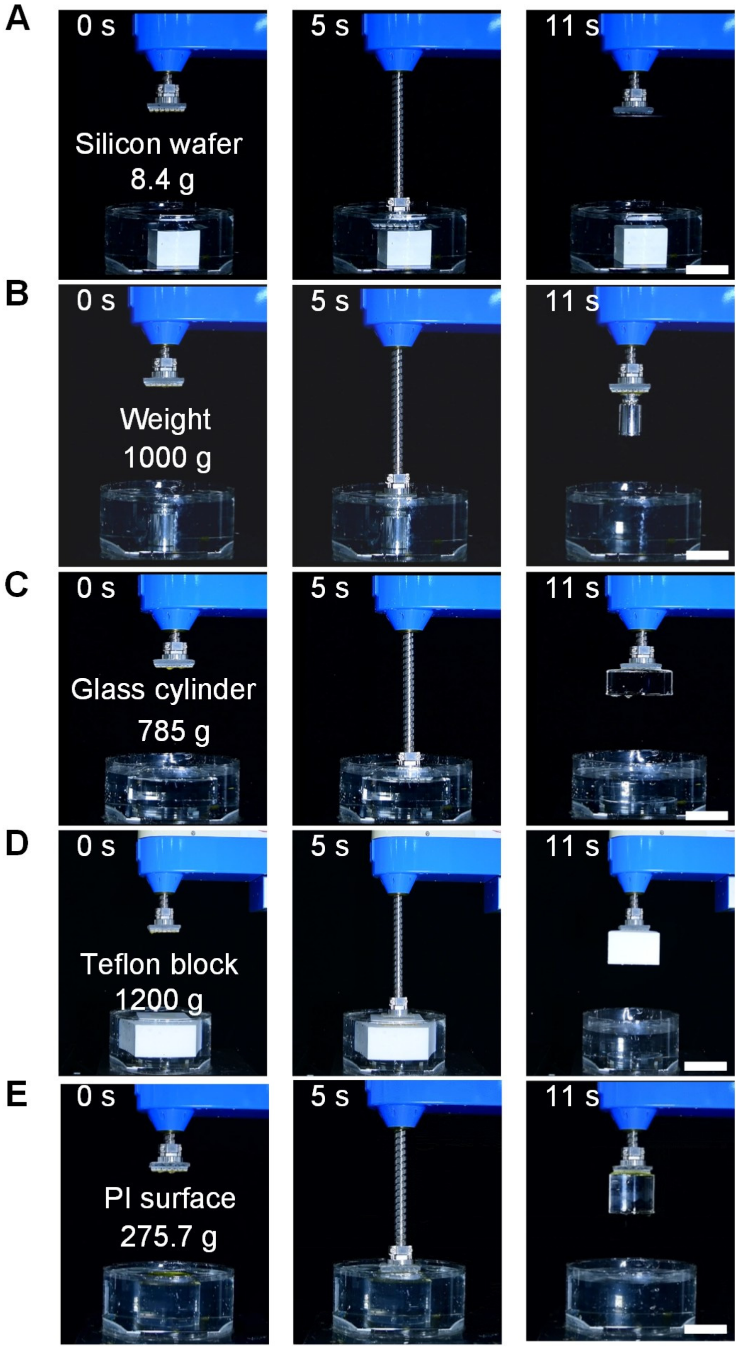

Fig. S15. A photographic time series of robot track vertically grasping different objects with different materials, weights and shapes underwater: 8.4-g silicon wafer (A); 1000-g weight (B); 785-g glass cylinder (C); 1200-g Teflon block (D) and 275.7-g PI (E). Scale bars: 6 cm.

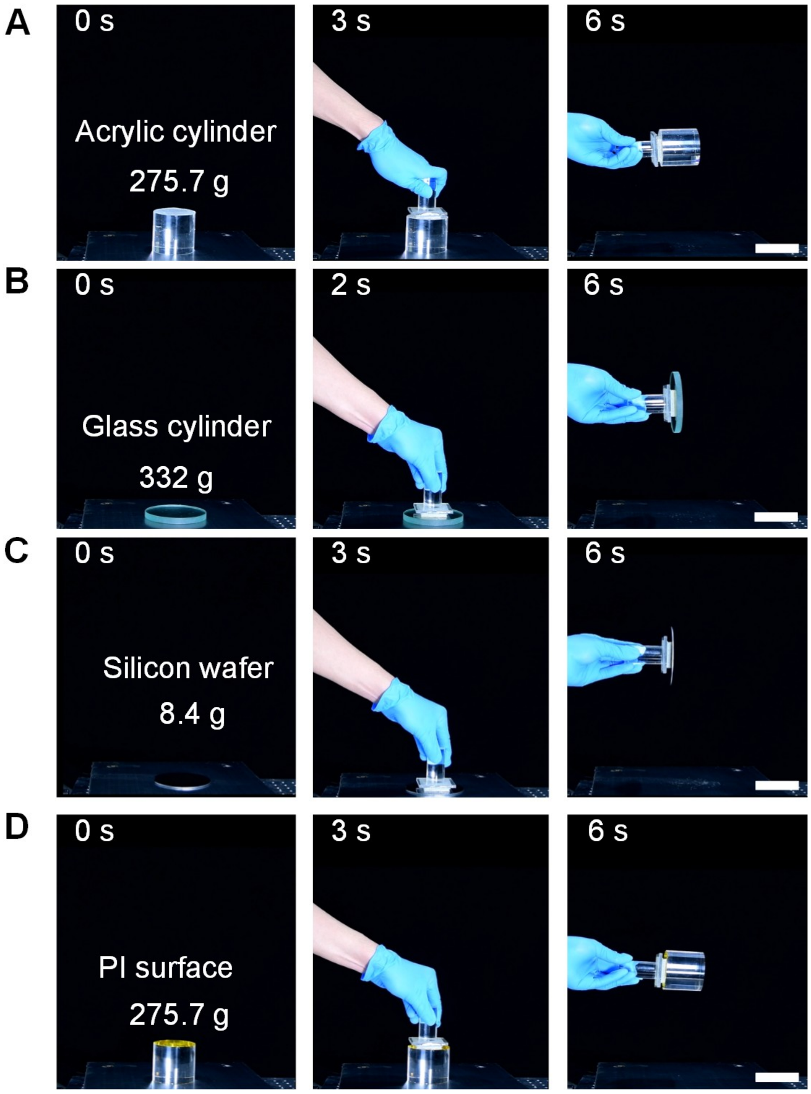

Fig. S16. A photographic time series of robot track tangentially grasping different objects with different materials, weights and shapes in dry environment: 275.7-g acrylic cylinder (A); 332-g glass cylinder (B); 8.4-g silicon wafer (C) and 275.7-g PI (D). Scale bars: 6 cm.

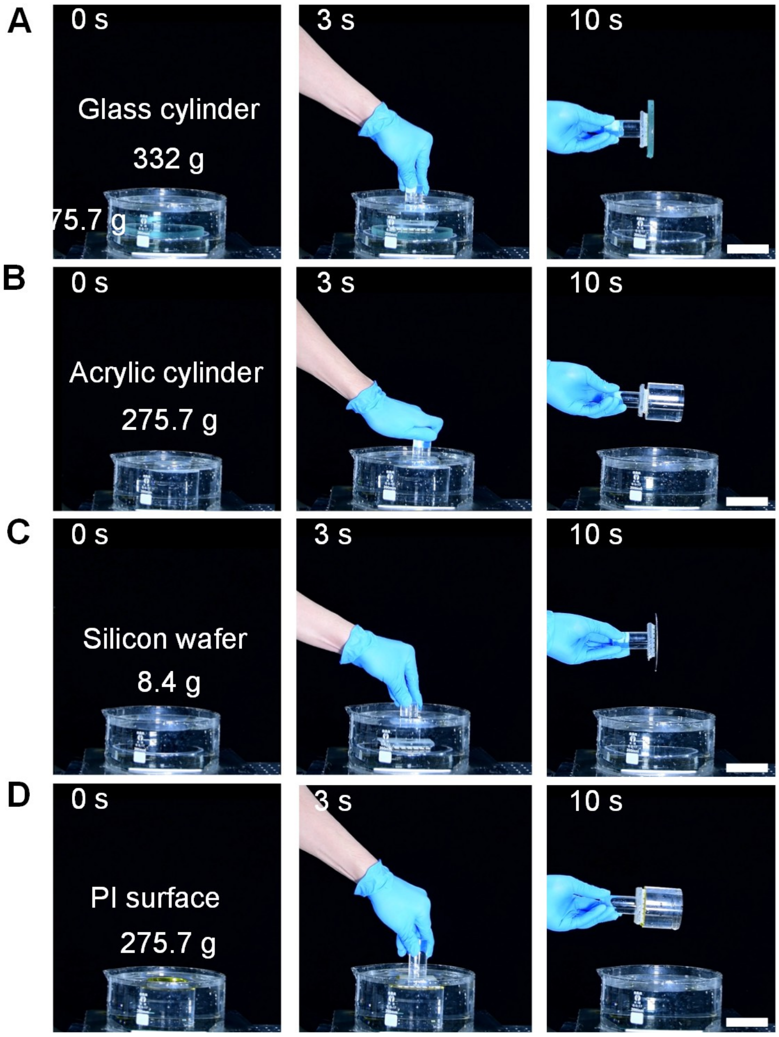

Fig. S17. A photographic time series of robot track tangentially grasping different objects with different materials, weights and shapes underwater: 332-g glass cylinder (A); 275.7-g acrylic cylinder (B); 8.4-g silicon wafer (C) and 275.7-g PI (D). Scale bars: 6 cm.

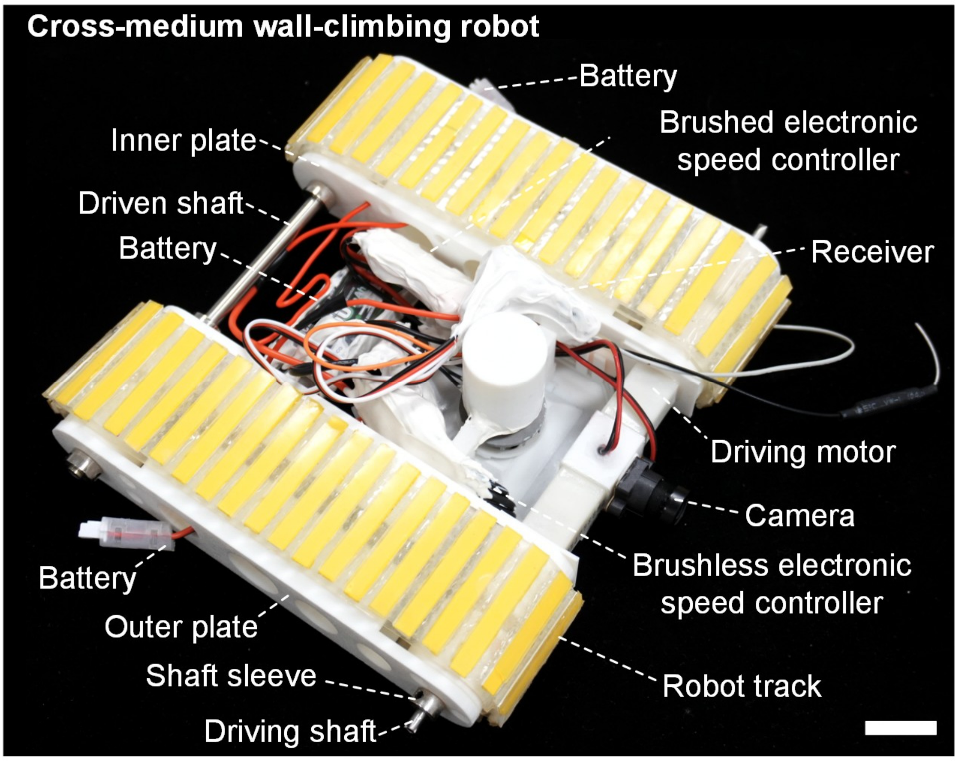

Fig. S18. Photograph of the cross-medium WCR after waterproof processing. Scale bar: 2 cm.

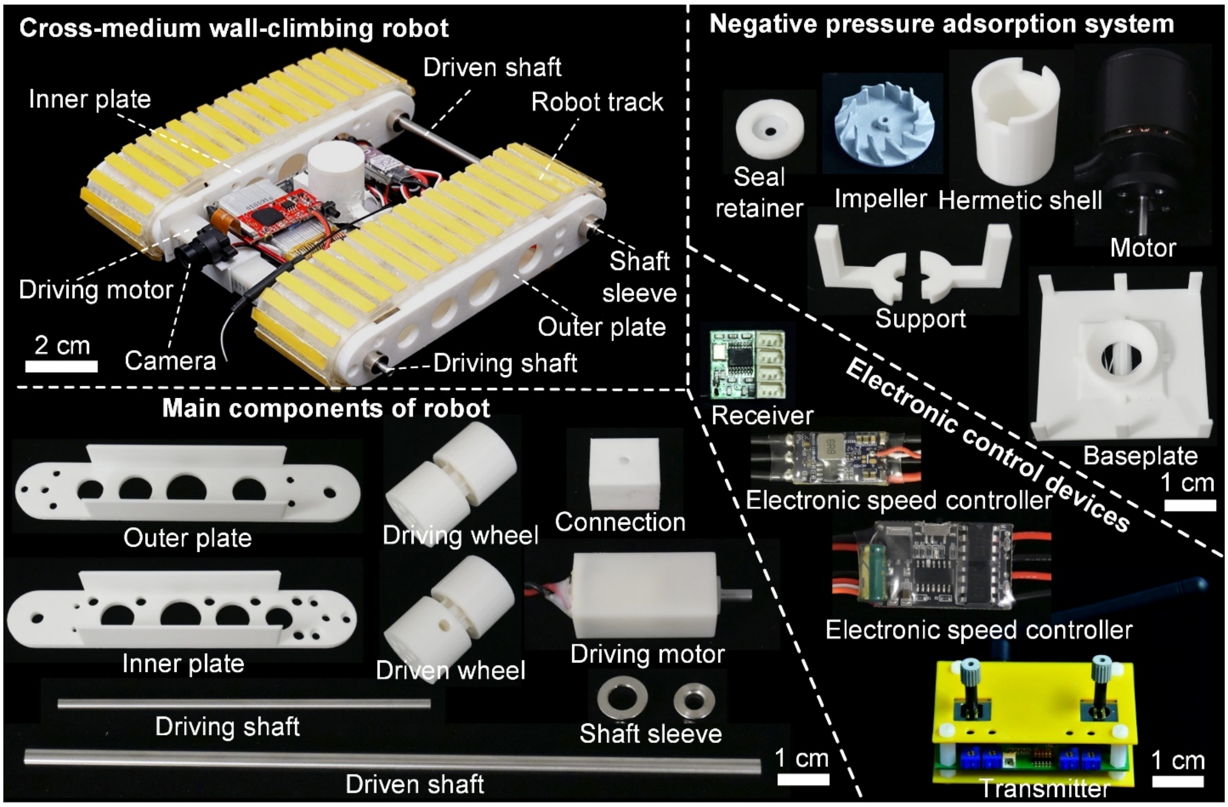

Fig. S19. Main components of the cross-medium WCR. The proposed WCR mainly includes 3D-printing plastic components, metal parts, motors, negative pressure absorption system, and electronic control devices.

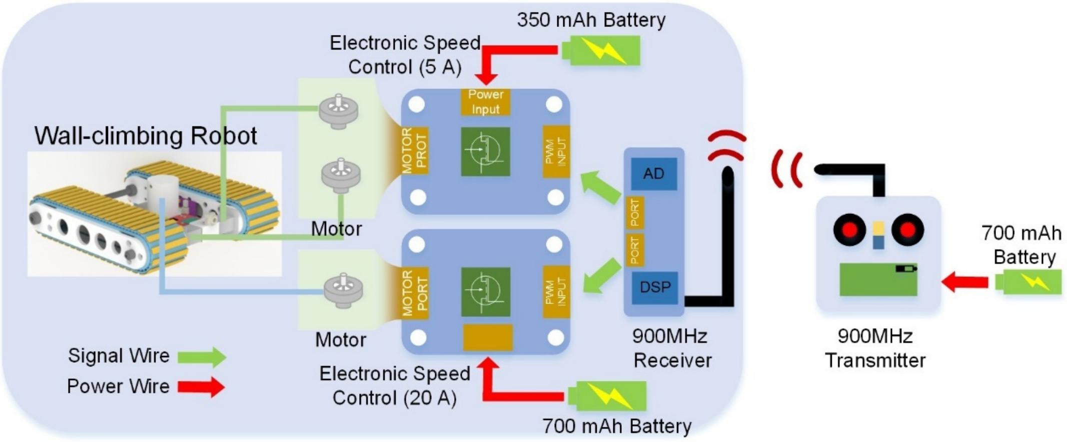

Fig. S20. The system diagram of the onboard electronics and power supply for the cross-medium WCR.

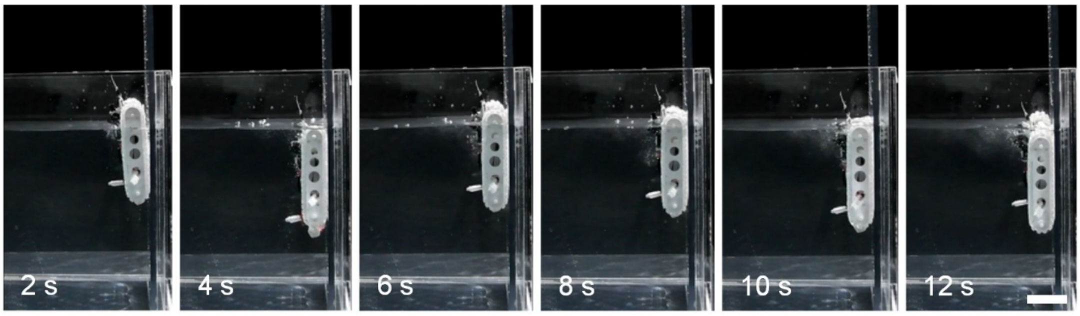

Fig. S21. Water-exit failure of the cross-medium WCR if no HMSAMS exists on the robot tracks. Scale bar: 6 cm.

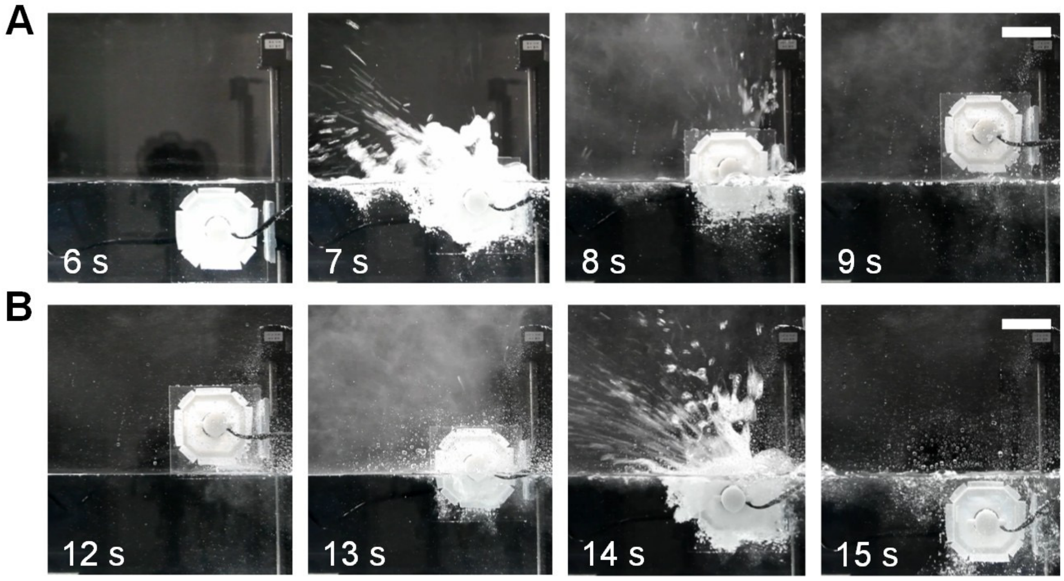

Fig. S22. Water-exit (A) and water-entry (B) images of negative pressure adsorption system. Scale bars: 3 cm.

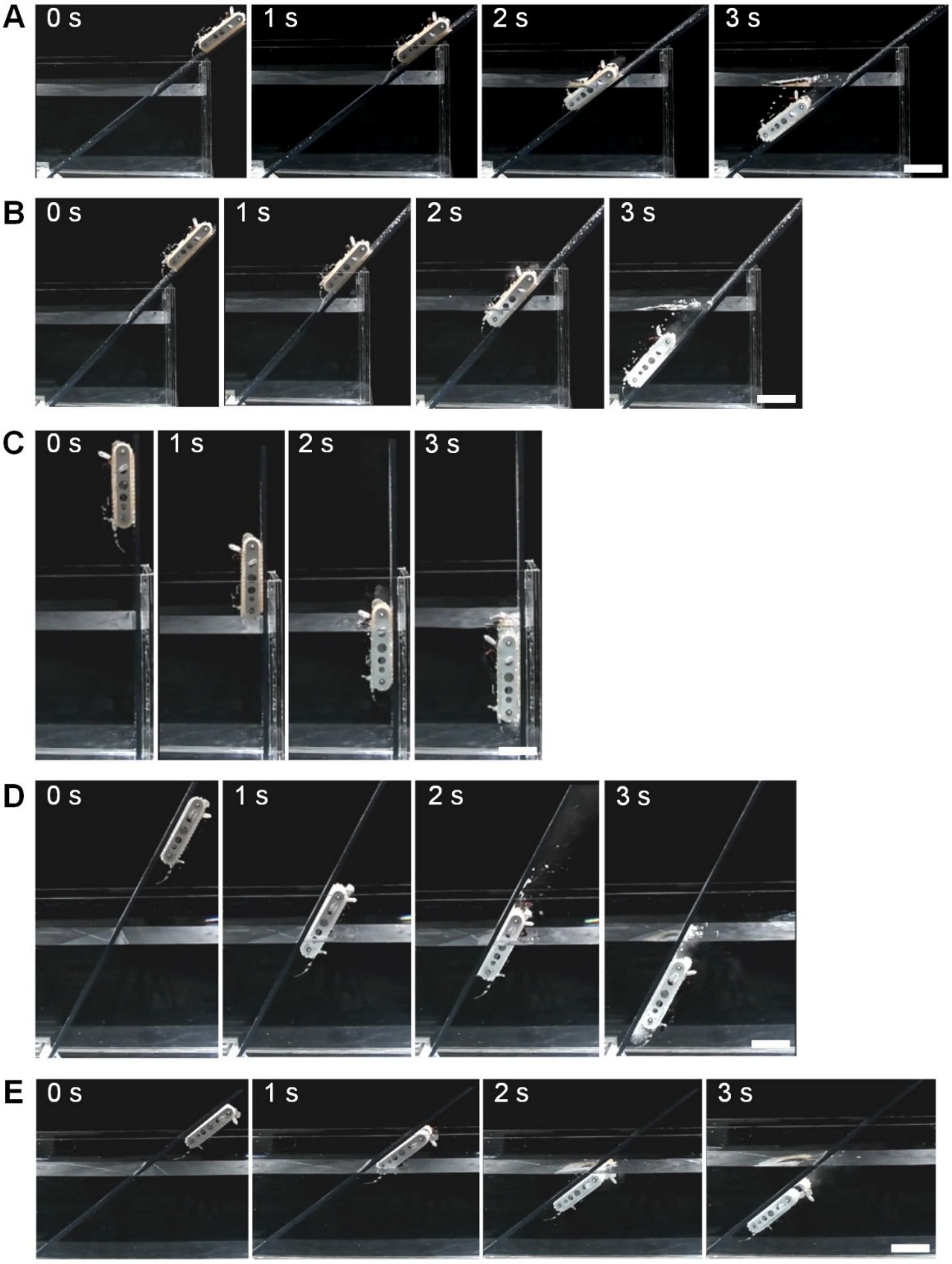

Fig. S23. Water-entry ability of proposed cross-medium WCR on positive and negative surfaces of varying inclination angles: 40° (A), 60° (B), 90° (C), 60° (D) and 40° (E). Scale bars: 8 cm.

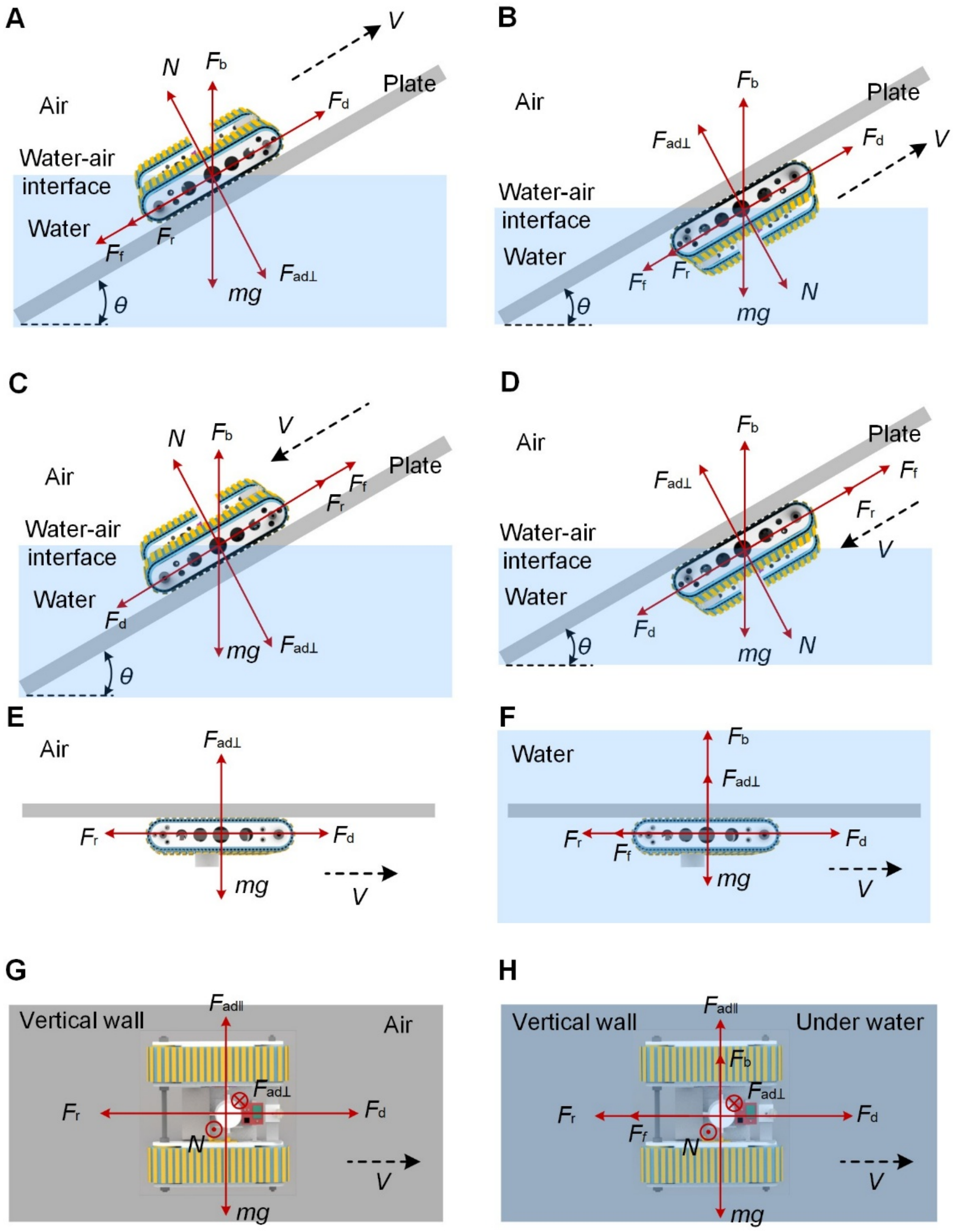

Fig. S24. Force analysis of cross-medium WCR. (A) WCR crawls from water into air on a positive surface (inclination angle: θ). **(B)** WCR crawls from water into air on a negative surface (inclination angle: θ). **(C)** WCR crawls from air into water on a positive surface (inclination angle: θ). **(D)** WCR crawls from air into water on a negative surface (inclination angle: θ). **(E)** WCR crawls on a negative surface in the air. **(F)** WCR crawls on a negative surface underwater. **(G)** WCR horizontally crawls on a vertical wall in the air. **(H)** WCR horizontally crawls on a vertical wall underwater. F_d, F_b, F_f, N, m, g, $F_{ad\perp}$, and F_r represent the driving force, buoyancy force, fluid resistance, support force, mass, gravitational acceleration, normal adhesion forces, and rolling resistance of WCR, respectively.

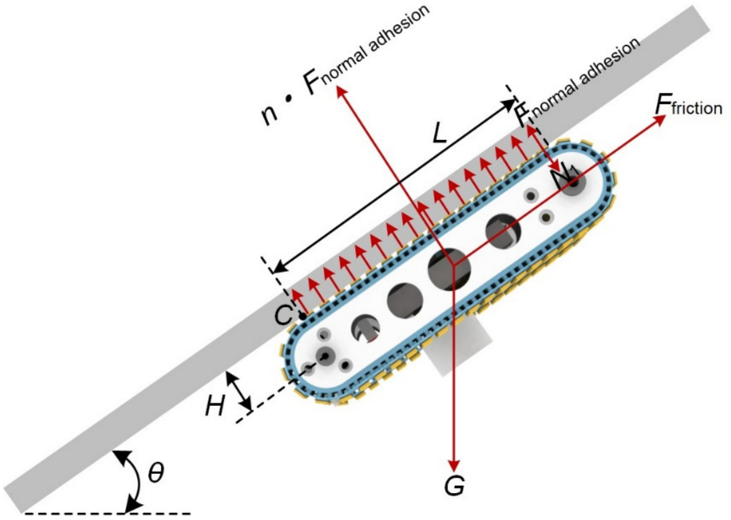

Fig. S25. Static force analysis of cross-medium WCR attaching to an inclined negative surface (inclination angle: *θ*). Ideally, each core-shell HMSAMS patch on the robot track exhibits an equal normal adhesion force to the substrate surface. $F_{whole\ friction}$ and G respectively represent the friction and gravity of WCR. $F_{normal\ adhesion}$ and N_1 respectively represent the normal adhesion and support force of a core-shell HMSAMS patch. H represents the distance between the gravity center of robot and substrate surface. *n* represents the number of core-shell HMSAMS patch on the robot track attaching to the substrate surface.

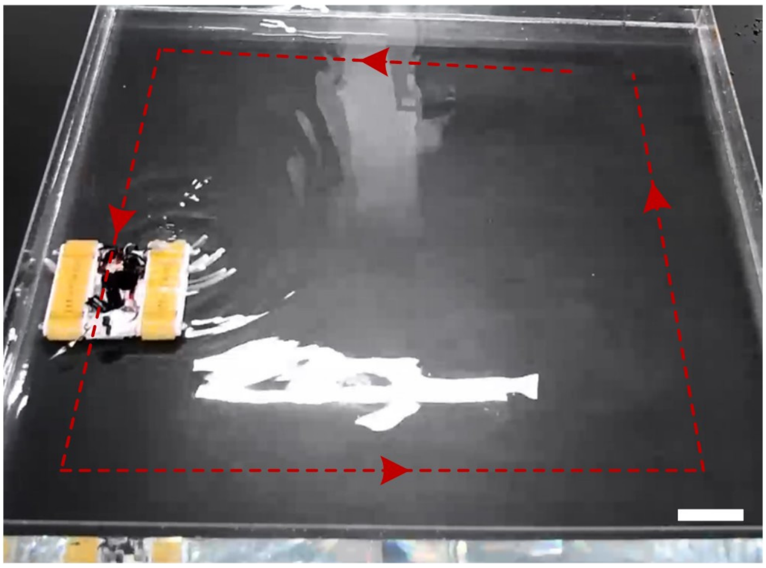

Fig. S26. Steering ability of the cross-medium WCR. The robot crawls along the perimeter of the water tank. Scale bar: 8 cm.

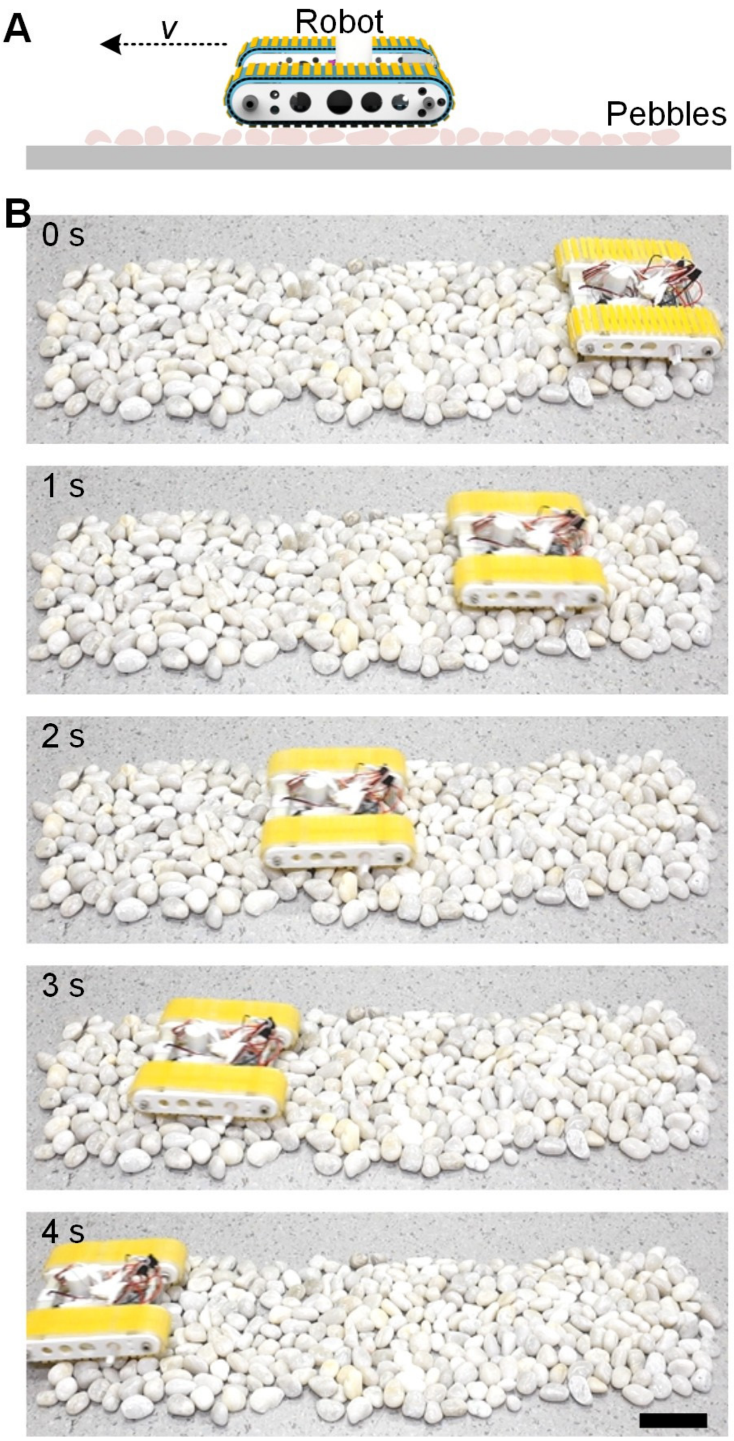

Fig. S27. The cross-medium WCR harnessing discrete robot tracks crawls on the rough surface. (A) Schematic diagram of the proposed robot crawling on a road covered with plenty of pebbles. **(B)** A photographic time series of the proposed robot crawling on pebble-covered rough surfaces (diameter of pebbles: ~2 cm). Scale bar: 8 cm.

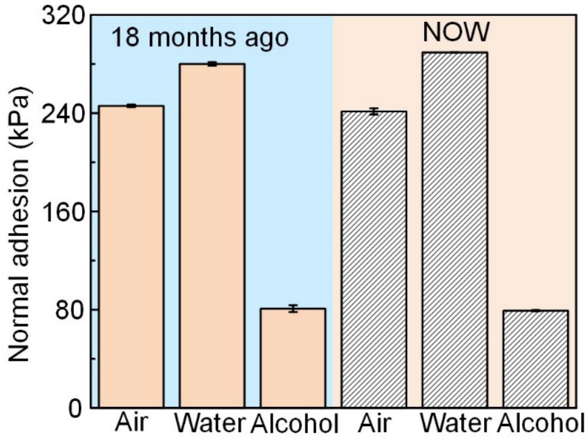

Fig. S28. Long-term (18-month) adhesion performance evaluation of HMSAMS patch in different media.

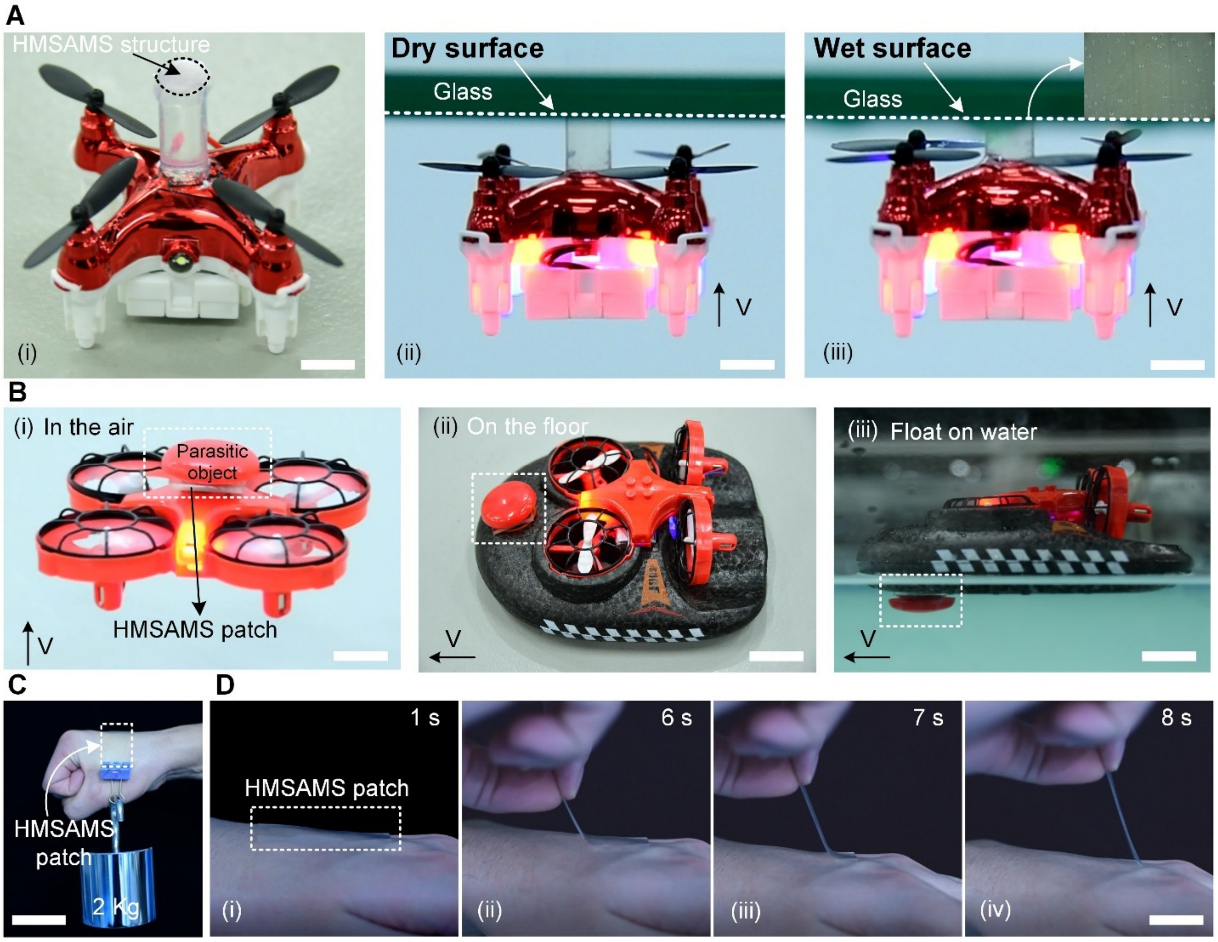

Fig. S29. Potential applications of HMSAMS patch. (A) Demonstration of unmanned aerial vehicle (UAV) equipped with HMSAMS patch attaching to dry and wet surfaces. Scale bars: 1 cm. **(B)** Parasitic object can attach to the surface of UAV and amphibious boat relying on HMSAMS patch. Scale bars: 1 cm. **(C)** The HMSAMS patch (length: 3 cm, width: 1 cm) attaching to the wet back of hand can bear 2-kg weight. Scale bar: 2 cm. **(D)** Demonstration of the HMSAMS patch (length: 5 cm, width: 2 cm) detaching from the back of hand. Scale bars: 2 cm.

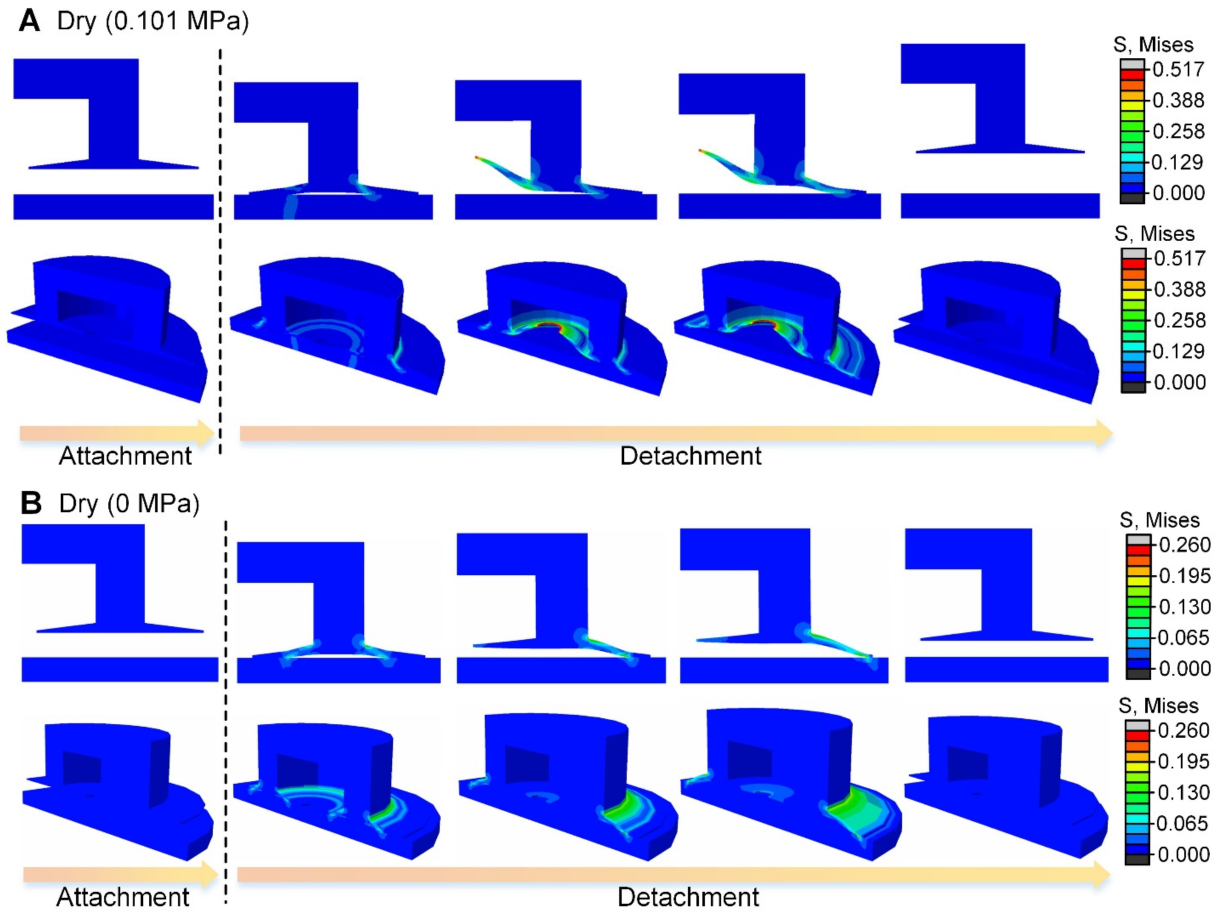

Fig. S30. Stress distribution and deformation of a single HMSAMS during attachment and detachment under different atmospheric pressures (A: 0.101 MPa, and B: 0 MPa) in dry environment.

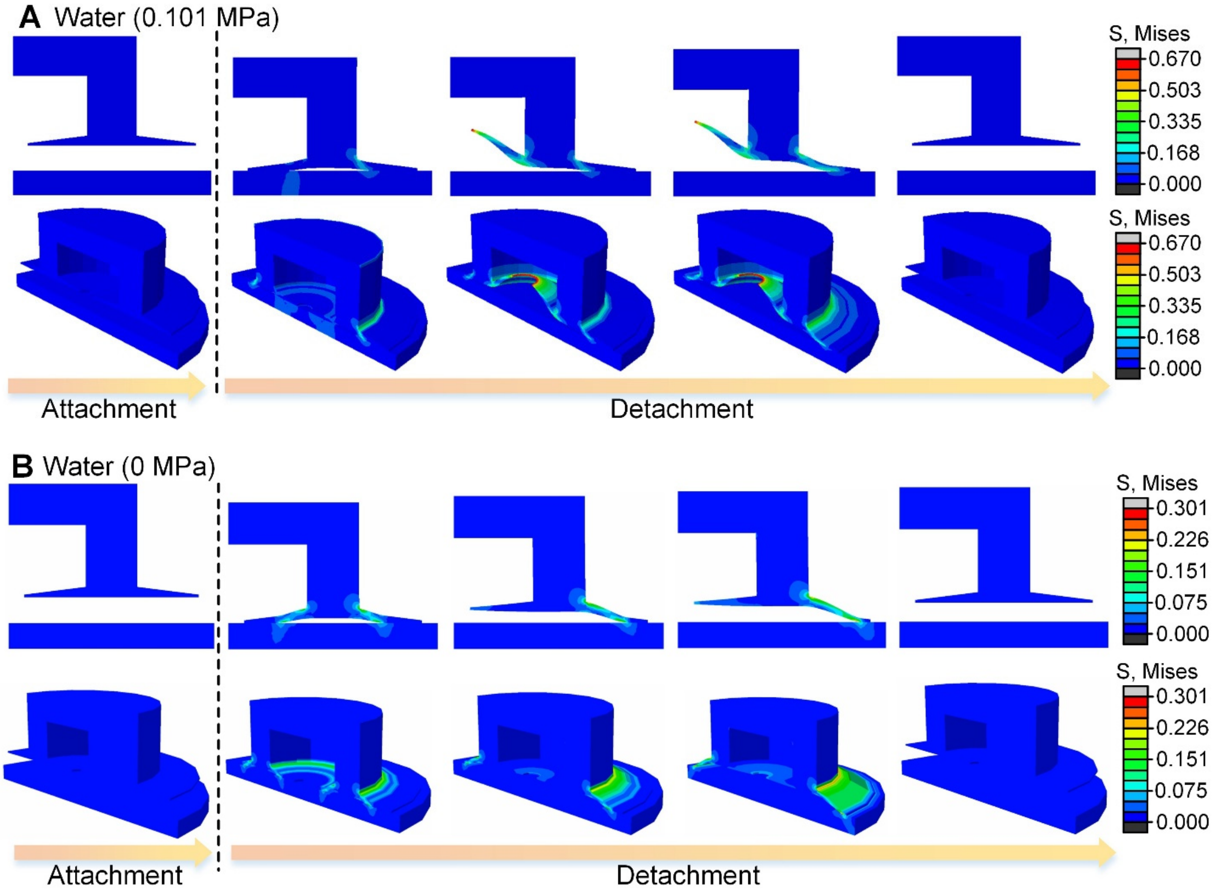

Fig. S31. Stress distribution and deformation of a single HMSAMS during attachment and detachment under different atmospheric pressures (A: 0.101 MPa, and B: 0 MPa) underwater.

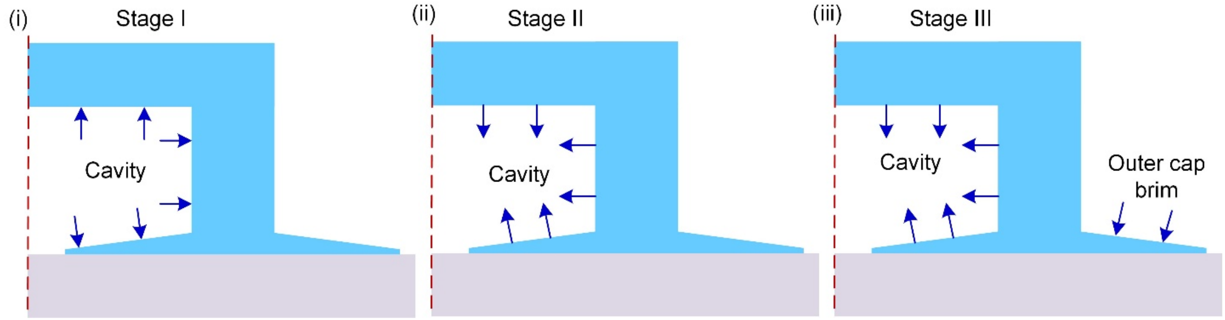

Fig. S32. Pressure variation of the HMSAMS at different stages during the simulation. When a vertical preload is applied to the top of the HMSAMS at stage I, the pressure inside the cavity is larger than the air pressure (i.e., $\Delta p>0$). During the vertical lifting process of the HMSAMS (stage II), the cavity pressure difference transitions from positive to negative (i.e., $\Delta p<0$). As the inner cap brim peels off from the substrate (stage III), a positive pressure is applied to the outer cap brim.

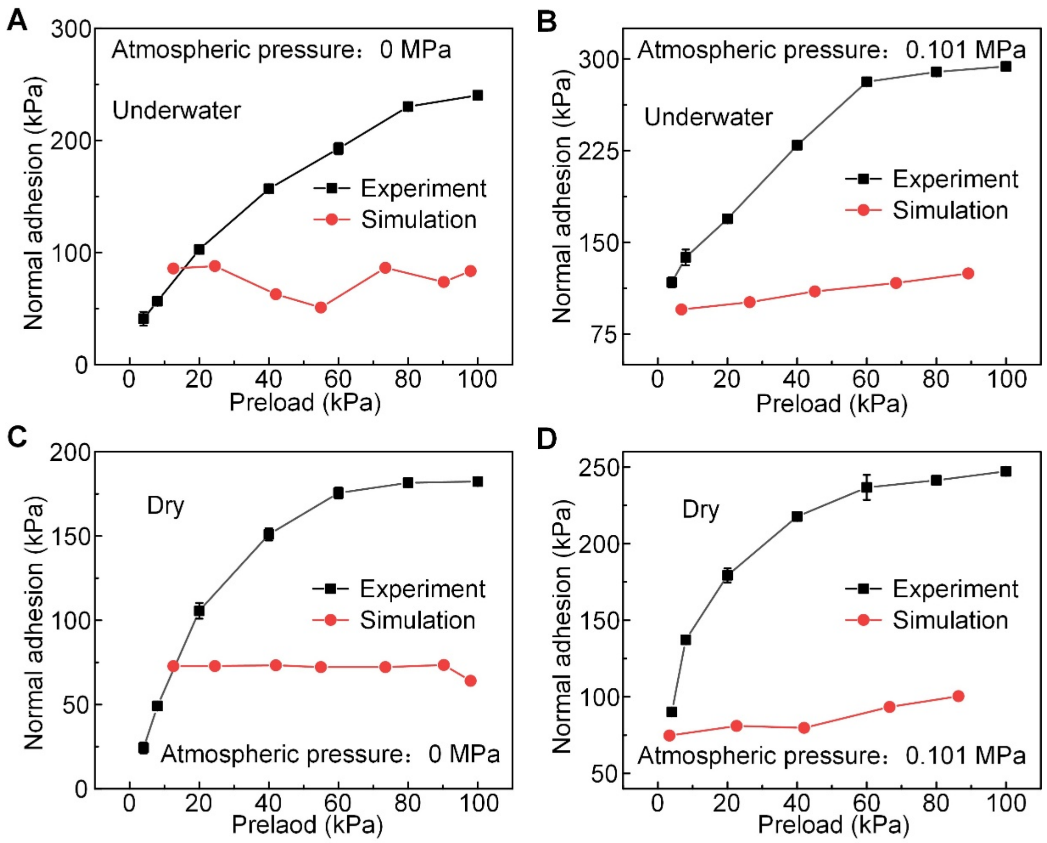

Fig. S33. Normal adhesion of HMSAMS patch in experiment and simulation. (**A**) Normal adhesion of HMSAMS patch underwater (atmospheric pressure: 0 MPa). (**B**) Normal adhesion of HMSAMS patch underwater (atmospheric pressure: 0.101 MPa). (**C**) Normal adhesion of HMSAMS patch in dry environment (atmospheric pressure: 0 MPa). (**D**) Normal adhesion of HMSAMS patch in dry environment (atmospheric pressure: 0.101 MPa).

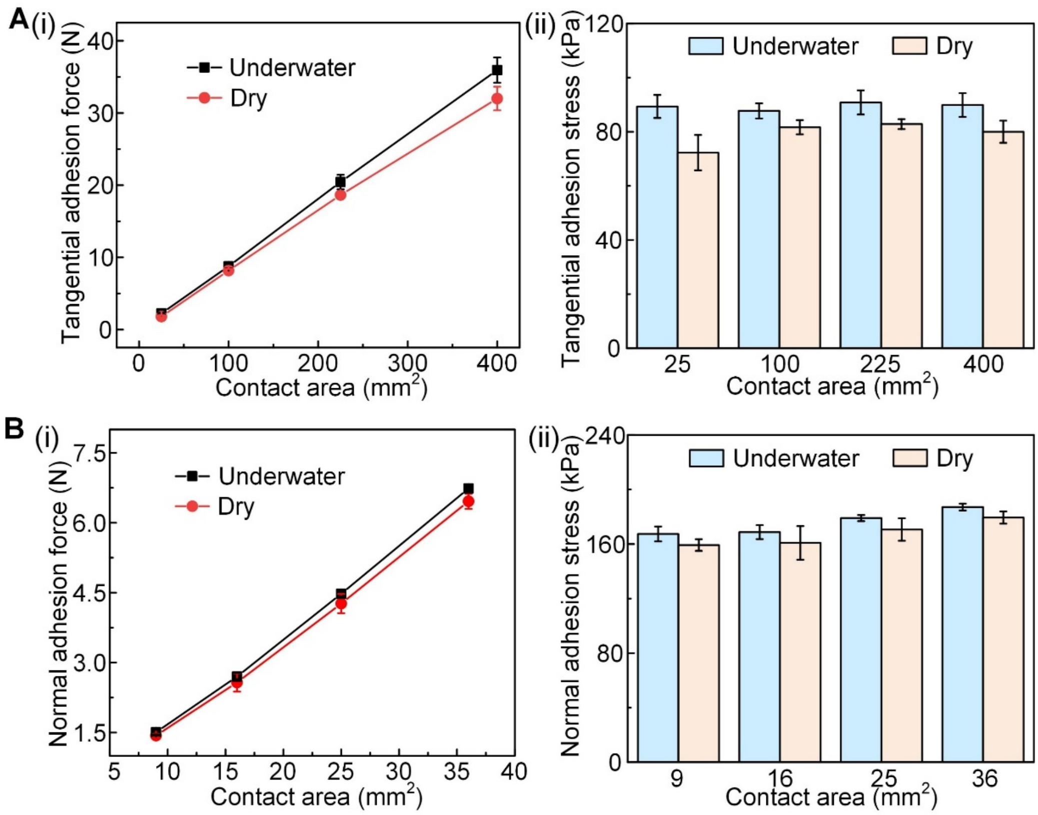

Fig. S34. Influence of contact area on adhesion performance. (**A**) Tangential adhesion force (i) and stress (ii) of HMSAMS patch with different areas in dry and underwater environments. (**B**) Normal adhesion force (i) and stress (ii) of HMSAMS patch with different areas in dry and underwater environments.

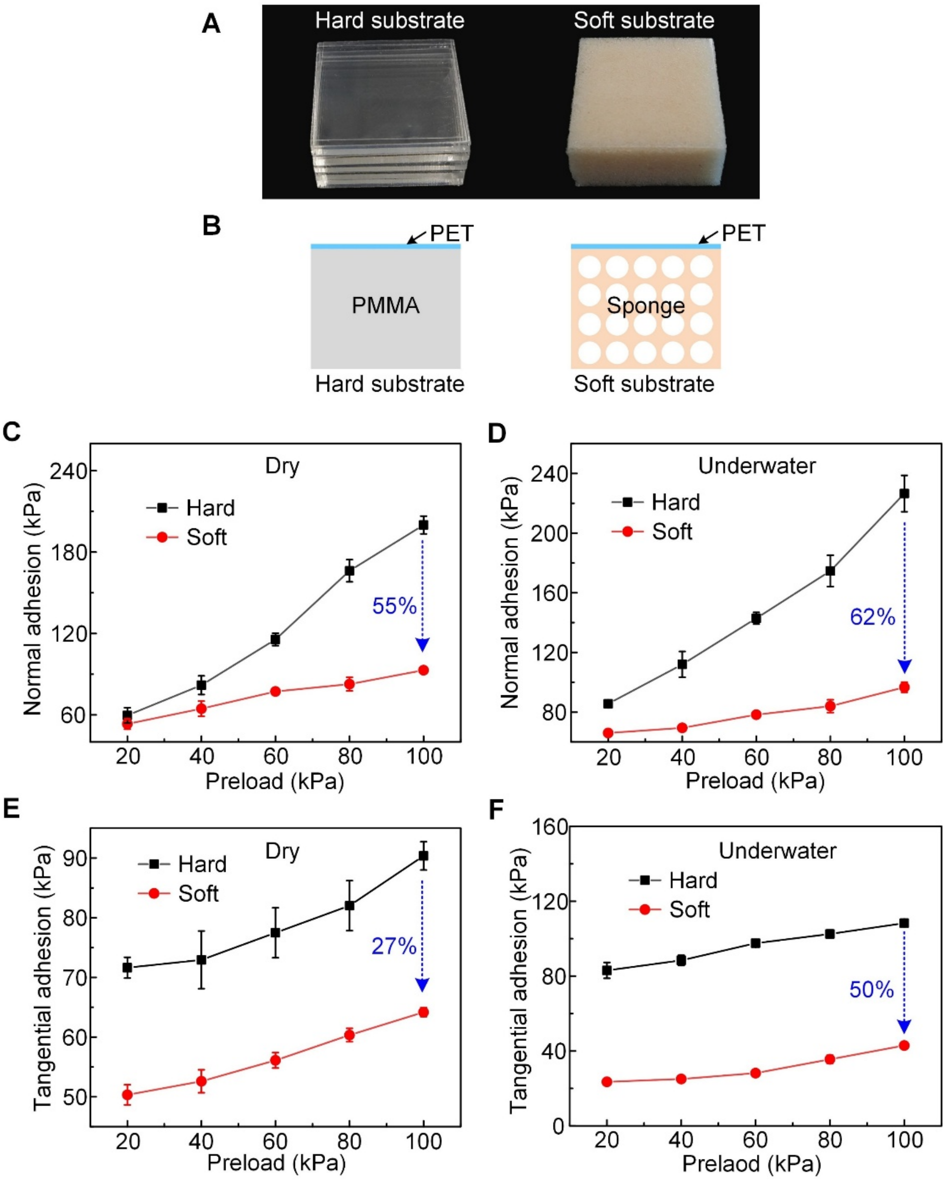

Fig. S35. Influence of soft and hard substrates on adhesion performance of HMSAMS patch. (**A**) Physical images of soft and hard substrates. (**B**) Cross sections of soft and hard substrates. (**C**) Normal adhesion-preload curves of HMSAMS patch measured on the soft and hard substrates in dry environment. (**D**) Normal adhesion-preload curves of HMSAMS patch measured on the soft and hard substrates underwater. (**E**) Tangential adhesion-preload curves of HMSAMS patch measured on the soft and hard substrates in dry environment. (**F**) Tangential adhesion-preload curves of HMSAMS patch measured on the soft and hard substrates underwater. The adhesion strength decreases approximately 55% for A, 62% for B, 27% for C, and 50% for D, respectively.

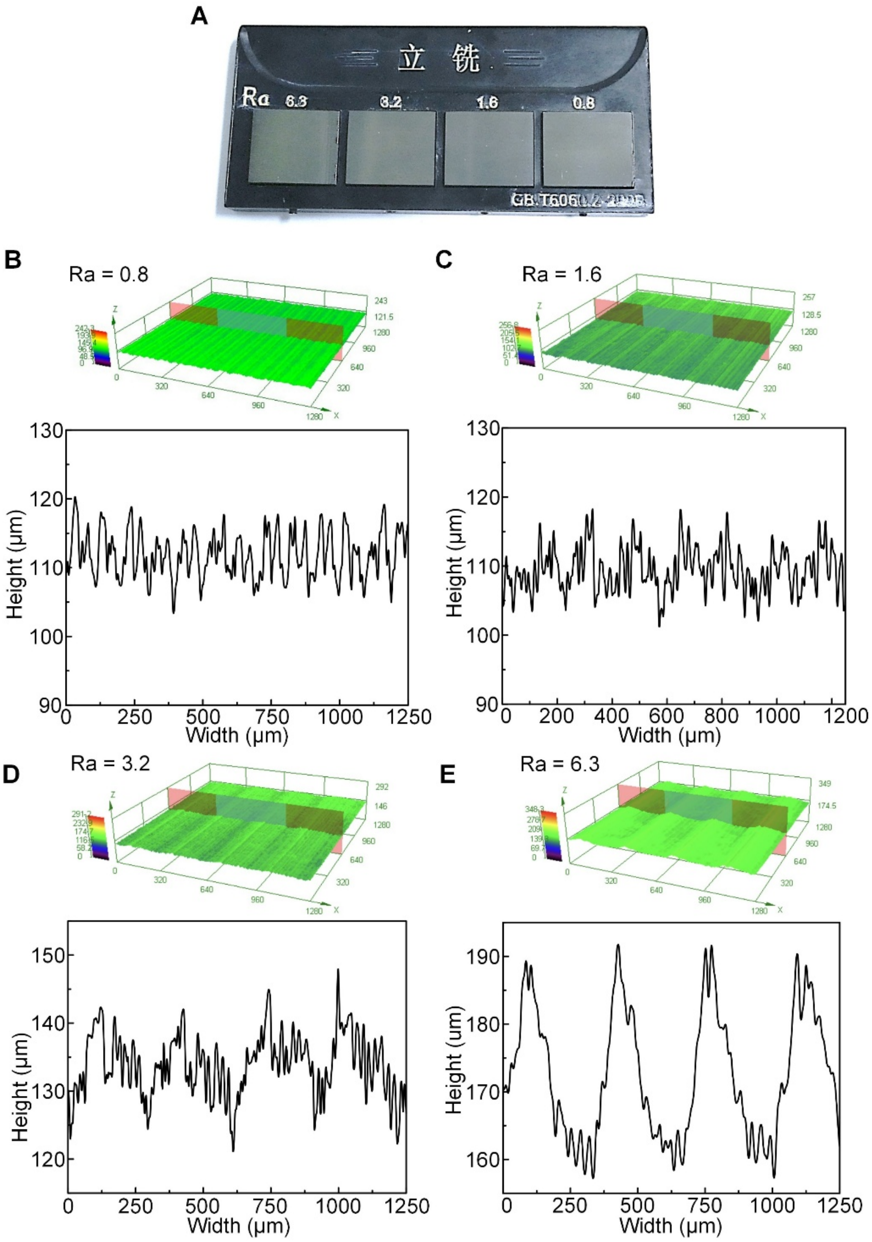

Fig. S36. Surface roughness test sample blocks. (**A**) Physical images of the roughness test sample blocks (Ra = 0.8 µm, 1.6 µm, 3.2 µm, 6.3 µm). (**B-E**) The microscopic morphology of surfaces with different roughness levels.

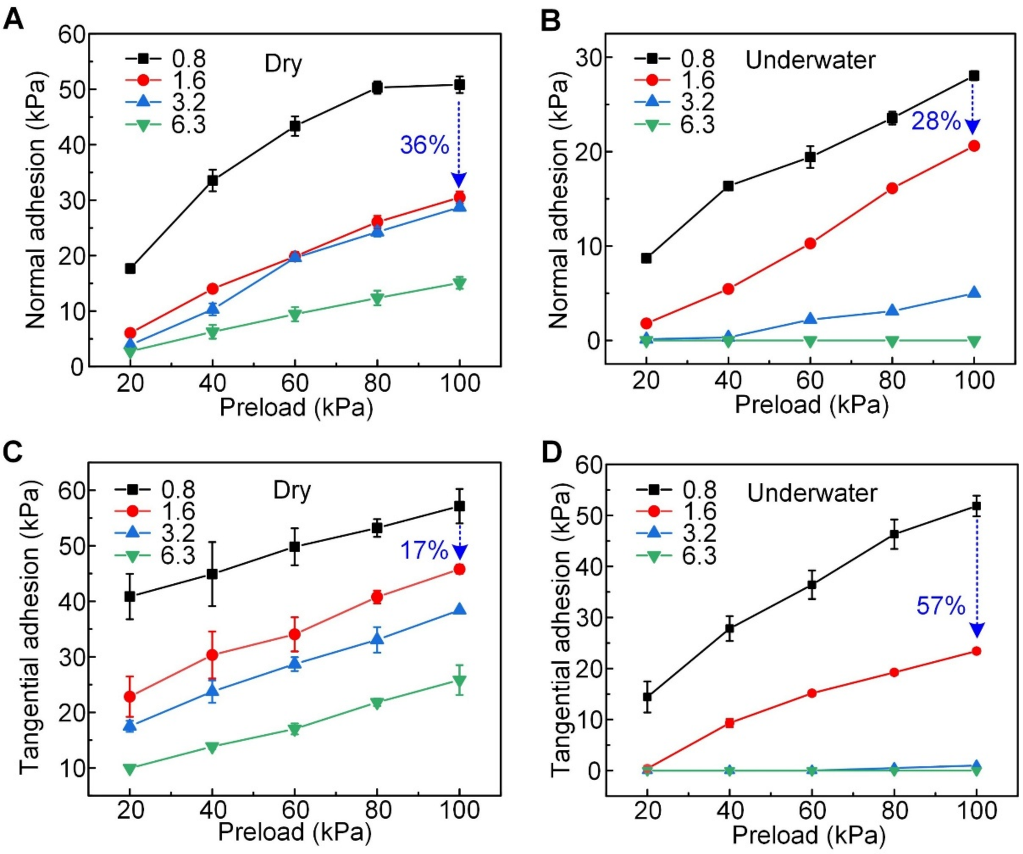

Fig. S37. Influence of surface roughness (Ra = 0.8 µm, 1.6 µm, 3.2 µm, 6.3 µm) on the adhesion performance of HMSAMS patch. (**A**) Normal adhesion-preload curves of the HMSAMS patch measured on surfaces with different roughness scales under dry condition. (**B**) Normal adhesion-preload curves of HMSAMS patch measured on surfaces with different roughness scales underwater. (**C**) Tangential adhesion-preload curves of HMSAMS patch measured on surfaces with different roughness scales under dry condition. (**D**) Tangential adhesion-preload curves of HMSAMS patch measured on surfaces with different roughness scales underwater. The adhesion strength decreases approximately 36% for A, 28% for B, 17% for C, and 57% for D, respectively as the roughness increases from 0.8 µm to 1.6 µm.

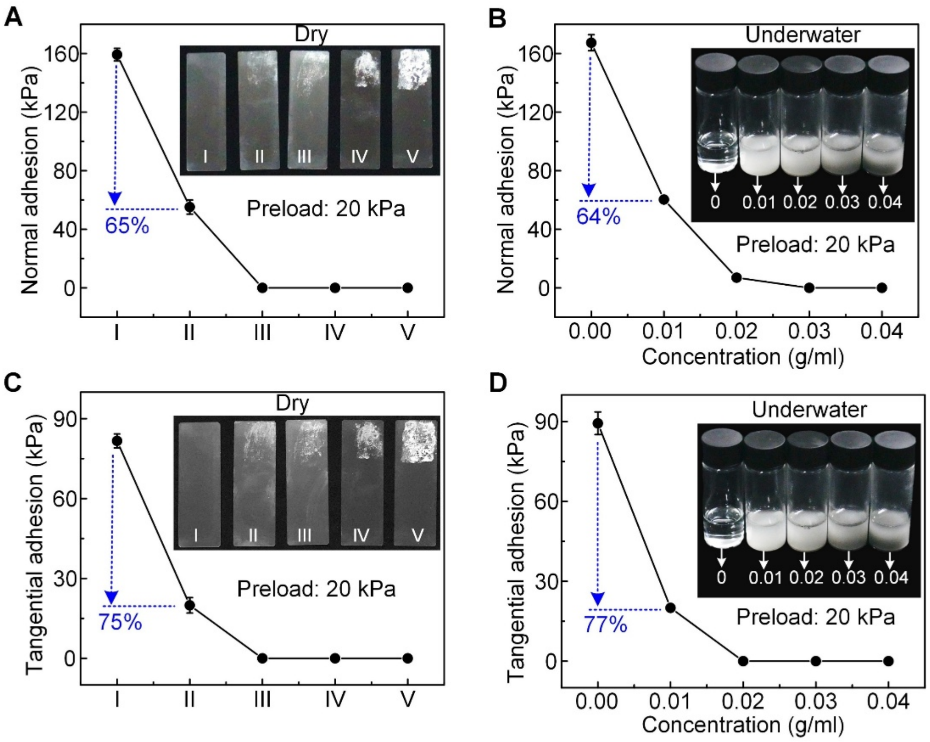

Fig. S38. The influence of dust on adhesion performance of HMSAMS patch. (**A**) Influence of the amount of dust on the normal adhesion of HMSAMS patch in dry environment. (**B**) Influence of dust concentration on the normal adhesion of HMSAMS patch underwater. (**C**) Influence of the amount of dust on the tangential adhesion of HMSAMS patch in dry environment. (**D**) Influence of dust concentration on the tangential adhesion of HMSAMS patch underwater. The adhesion strength decreases approximately 65% for A, 64% for B, 75% for C, and 77% for D, respectively.

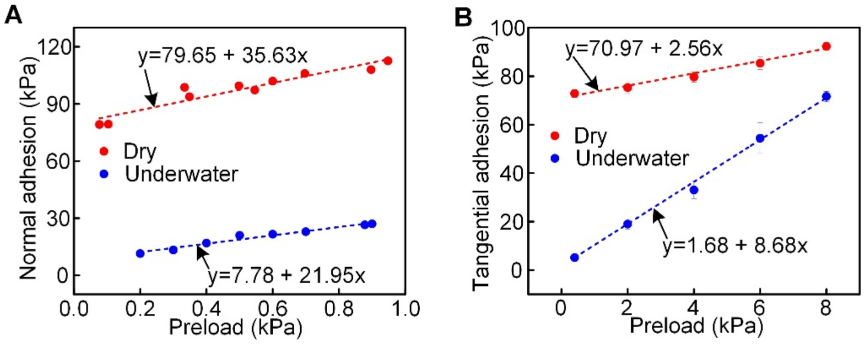

Fig. S39. Normal and tangential adhesion of HMSAMS patch under dry and underwater conditions and corresponding fitted linear equations. (**A**) Normal adhesion of HMSAMS patch in dry and underwater environments under different preloads. (**B**) Tangential adhesion of HMSAMS patch in dry and underwater environments under different preloads.

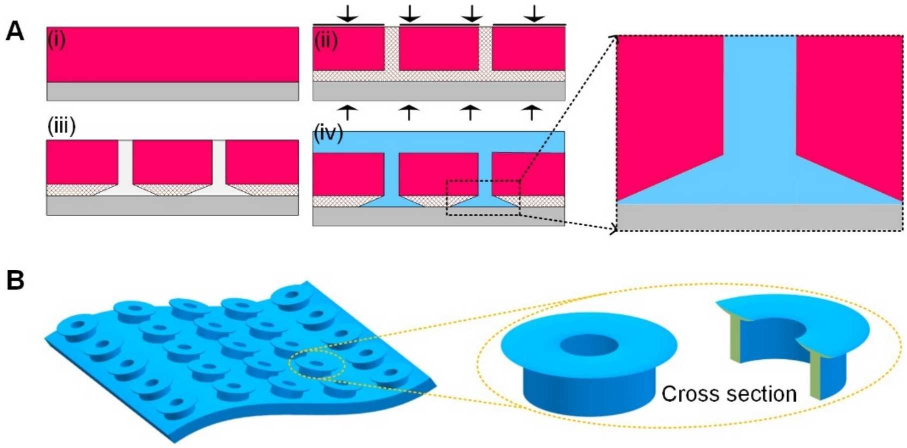

Fig. S40. Fabrication process and morphology of hollow mushroom-shaped adhesive microstructures (HMSAMSs). (A) Fabrication process of HMSAMSs. (i) The photoresist AZ 4620 (AZ Electronic Materials) was spin-coated onto glass (10-nm-thick Cr was evaporated on one side of glass surface) at 1000 rpm for 45 s, followed by soft baking at 95 °C for 20 min. (ii) Then, the photoresist was exposed to G-line ultraviolet light at a power of 10 mJ cm^{-2} for 37 s in the presence of a mask. Further, maskless exposure was performed from the bottom side of glass for 4 s. (iii) The following development was performed in a sodium hydroxide solution (0.5 wt %) for about 240 s, generating a hollow mushroom-shaped negative mold. (iv) The mixed PDMS (Dow Corning, Sylgard 184 kit, 10:1 mass ratio of base to cross-linker) was spin-coated onto the developed photoresist. Finally, the adhesive patch was obtained in a vacuum chamber for 10 min and then cured at 85 °C for 4 h. **(B)** The morphology of HMSAMSs.

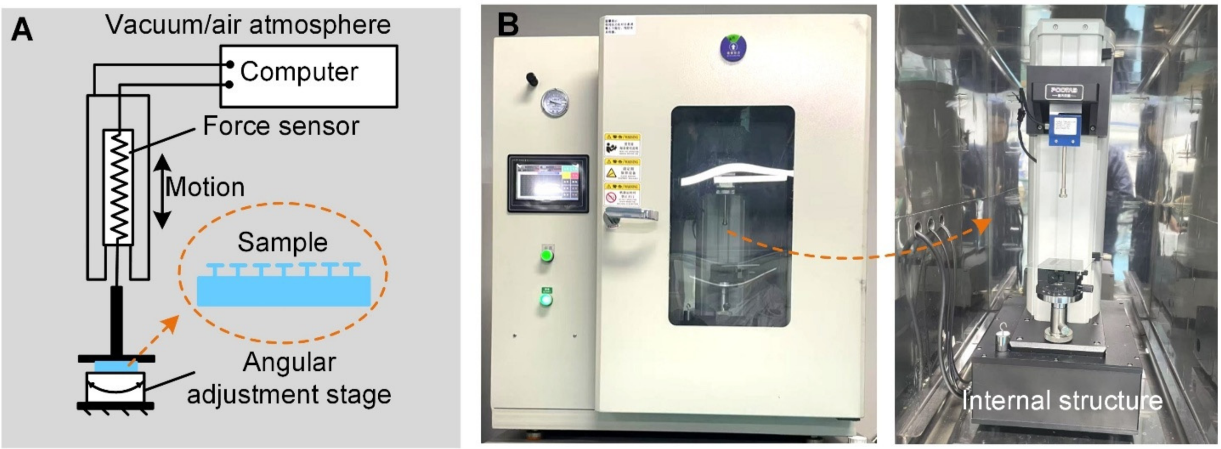

Fig. S41. Testing equipment of adhesion force under different atmospheric pressures. The diagram (A) and photographs (B) of testing equipment.

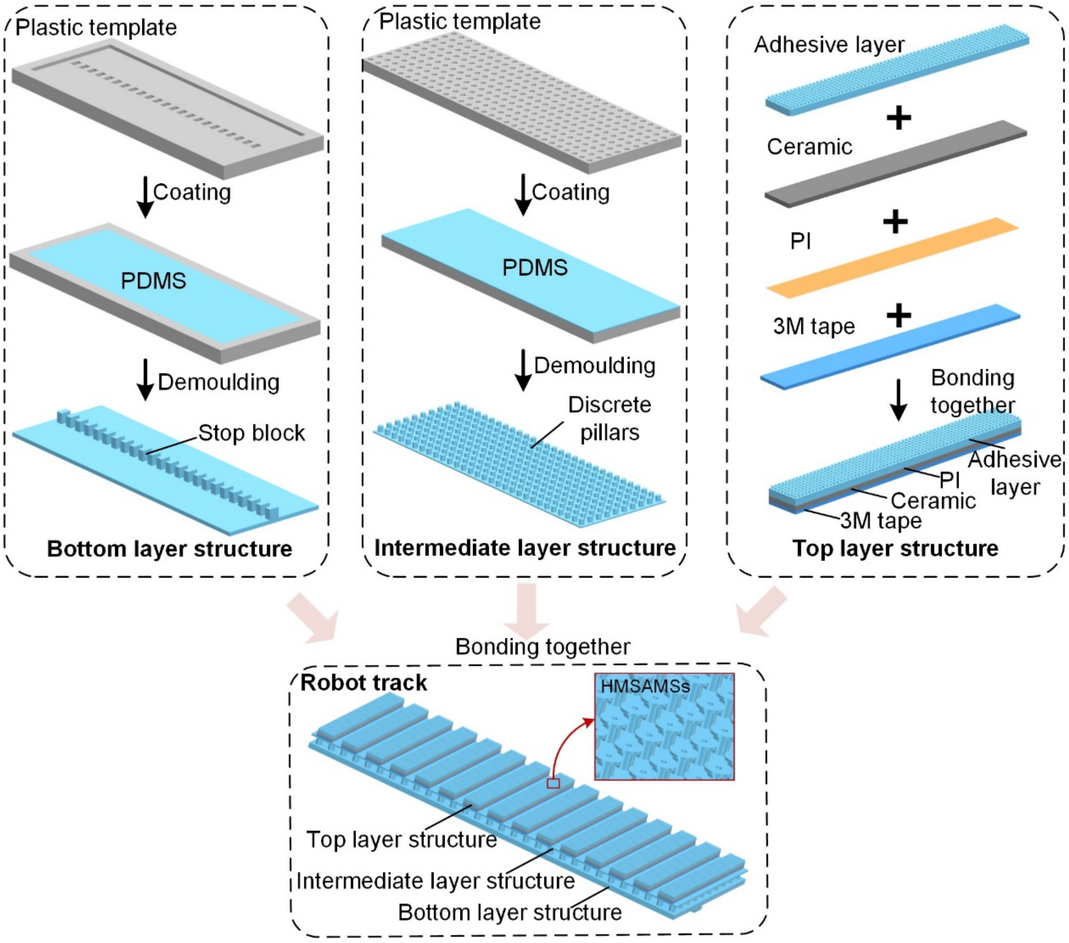

Fig. S42. Fabrication process of the robot track. The bottom layer structure (i.e. PDMS film with stop block) and intermediate layer structure (i.e. PDMS film with discrete pillars) were obtained by plastic templates, and the top layer structure was integrated by adhesive layer, ceramic plate, PI tape and 3M tape. Subsequently, the robot track was fabricated by bonding the top, intermediate and bottom layer structures together.

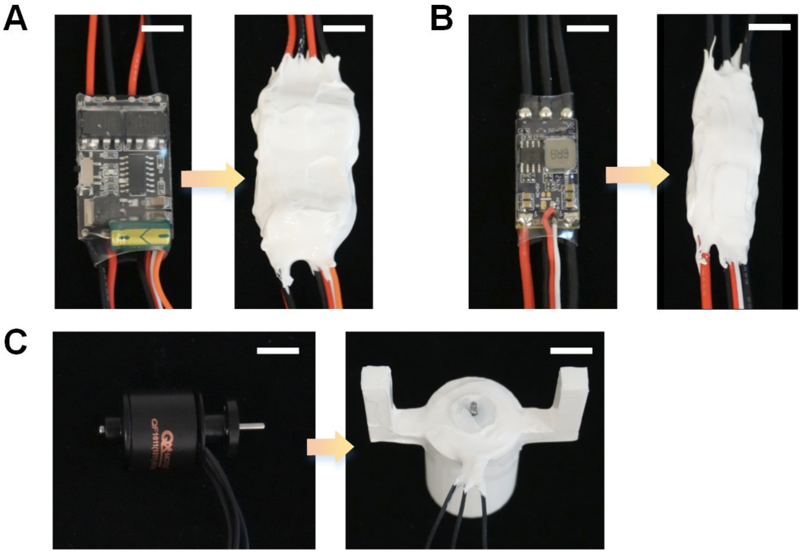

Fig. S43. Waterproof processing of brushed electronic speed controller (ESC) (A), brushless ESC (B) and brushless motor (C) by waterproof glue (Kafuter, k946). Scale bars: 1 cm.

Movie S1. Demonstration of normal adhesion performance of HMSAMS patch in dry condition.
Movie S2. Demonstration of normal adhesion performance of HMSAMS patch underwater.
Movie S3. Demonstration of tangential adhesion performance of HMSAMS patch in dry condition.
Movie S4. Demonstration of tangential adhesion performance of HMSAMS patch underwater.
Movie S5. Demonstration of normal and tangential adhesion performance of robot track underwater.
Movie S6. Demonstration of normal and tangential adhesion performance of robot track in dry condition.
Movie S7. Normal grasping ability of robot track to different materials in dry condition.
Movie S8. Normal grasping ability of robot track to different materials underwater.
Movie S9. Tangential grasping ability of robot track to different materials in dry condition.
Movie S10. Tangential grasping ability of robot track to different materials underwater.
Movie S11. Wall-climbing ability of robot on vertical wall in dry condition.
Movie S12. Wall-climbing ability of robot on vertical surface underwater.
Movie S13. Cross-medium wall-climbing ability of robot on vertical surface.
Movie S14. Water-exit failure of WCR when no HMSAMS exists on robot track.
Movie S15. Water-entry and -exit processes of negative pressure adsorption system.
Movie S16. Water-exit movement of WCR on positive and negative surfaces of different inclination angles.
Movie S17. Water-entry movement of WCR on positive and negative surfaces of different inclination angles.
Movie S18. WCR crawling on negative surfaces in dry and underwater environments.
Movie S19. WCR crawling on sidewall in dry and underwater environments.
Movie S20. WCR crawling against the water current.
Movie S21. Load-bearing capacity of WCR.
Movie S22. Steering ability of WCR.
Movie S23. WCR crawling on uneven surface.
Movie S24. WCR crawling on pebble surfaces.
Movie S25. WCR crawling on different material surfaces.
Movie S26. WCR crawling on outdoor wall with water droplets.
Movie S27. WCR crossing the water-air boundary on water gauge.
Movie S28. Water-entry and -exit movements of WCR from different directions on nonmagnetic stainless steel surfaces.
Movie S29. WCR crawling on an indoor ceiling.
Movie S30. WCR crawling vertically in a narrow gap.
Movie S31. WCR crawling horizontally in a slender tube.
Movie S32. Low-power consumption applications of HMSAMS patch on robots.
